# Sleeping upside-down: Knockdown of a sleep-associated gene induces daytime sleep in the jellyfish *Cassiopea*

**DOI:** 10.1073/pnas.2505074122

**Published:** 2025-07-14

**Authors:** Michael J. Abrams, Aki Ohdera, Diana A. Francis, Owen Donayre, Henry Chen, Kevin Lu, Celeste Y. Hsu, Hannah Zeigler, Richard M. Harland

**Affiliations:** ^a^Department of Molecular and Cell Biology, University of California, Berkeley, CA 94720; ^b^Department of Mathematics, University of Arizona, Tucson, AZ 85721

**Keywords:** sleep, jellyfish, behavior, regulation, evolution

## Abstract

The conservation of sleep among diverse animals provides clear evidence for its physiological importance, but the extent of its regulatory conservation is unknown. The upside-down jellyfish *Cassiopea xamachana* sleeps, and this behavior is controlled by radially spaced marginal ganglia. After defining a sleep–wake threshold, we compared gene expression profiles of ganglia from animals deprived of sleep and found differential expression in many sleep-related genes including GABAergic, melatonergic, and cholinergic receptors. In particular, a nicotinic acetylcholine receptor alpha subunit-like (Chrnal-E) was strongly differentially expressed in sleep deprived animals. Animals deprived of sleep under both mechanical and light perturbations suggested *chrnal-E* as a key gene regulating sleep. We found that chemical cholinergic neuromodulators regulate pacemaker activity. Visualization of *chrnal-E* mRNA revealed it is expressed primarily within the ganglia, and that the area of expression expands after sleep deprivation. In order to confirm the functional role of *chrnal-E*, we developed RNAi for use in *Cassiopea* and determined that Chrnal-E promotes wakefulness. Finally, we field-sampled control and sleep deprived animals and found *chrnal-E* has lowest expression late at night in controls, while in sleep deprived animals, *chrnal-E* peaks at this time, supporting a link to wakefulness. Our finding that *Cassiopea* sleep is regulated by the cholinergic-like system underscores that some components of sleep regulation are deeply conserved even in an animal with a distributed nervous system.

## Significance

Sleep is a fundamental behavioral and physiological process conserved across diverse animals, yet its regulatory mechanisms remain unclear. We investigated the regulation of sleep in an early branching animal lineage with a relatively simple nervous system to gain insight into the evolution of sleep. The upside-down jellyfish *Cassiopea xamachana* exhibits sleep, behaviorally controlled by marginal ganglia. We focused on how sleep deprivation strongly altered ganglionic expression of a nicotinic acetylcholine receptor alpha-like subunit, *chrnal-E*. RNAi-mediated knockdown determined that Chrnal-E promotes wakefulness. Our findings suggest deep evolutionary conservation of cholinergic-like receptors in sleep regulation. Understanding how sleep is controlled in relatively simple organisms provides insight into its fundamental biological importance and may inform broader studies of neurobiology across species.

**Fig. 1.**
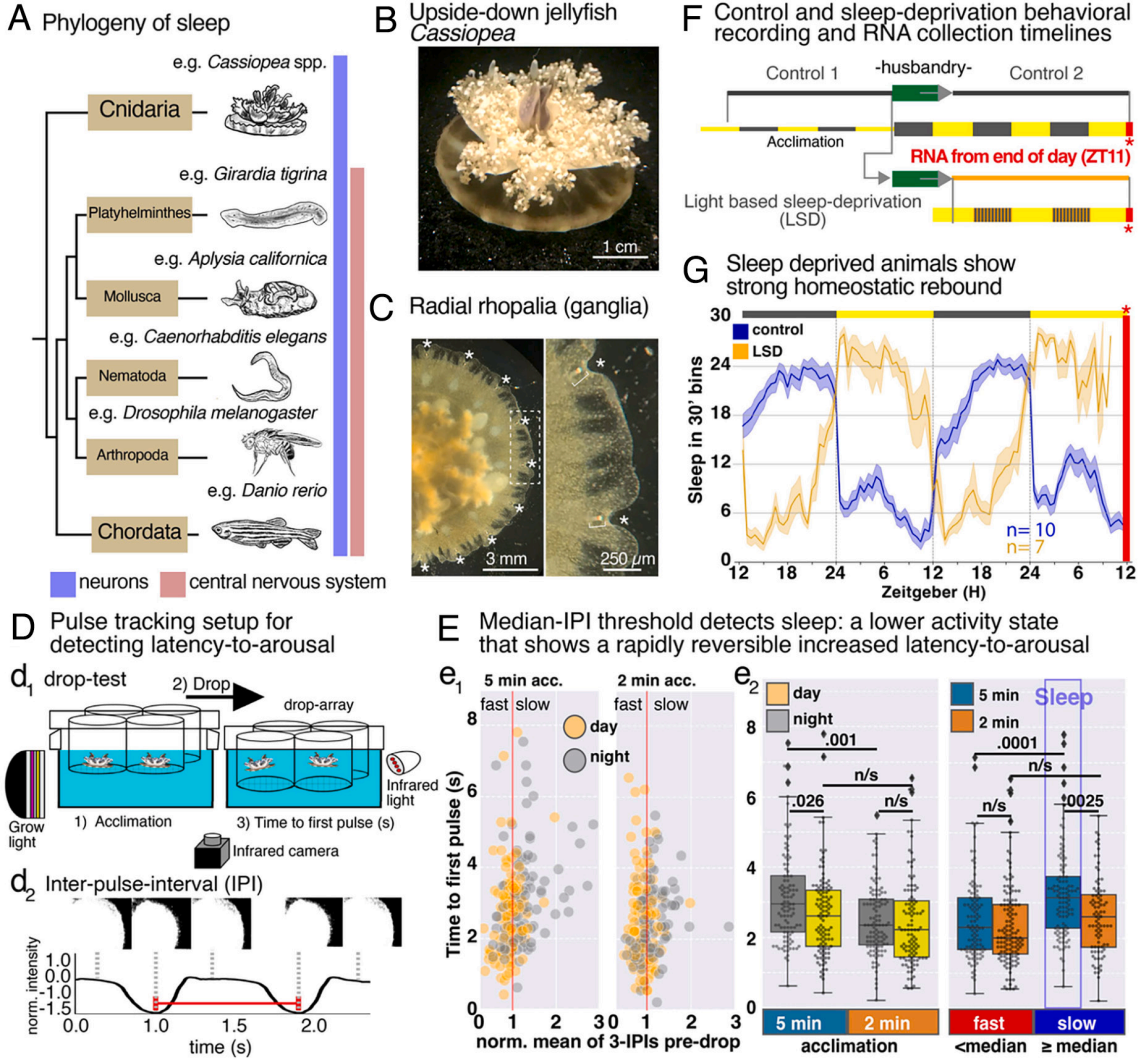
Cassiopea sleep. (*A*) Phylogenetic tree highlighting the six phyla with described sleep states, adapted from (7), and the lack of a CNS in *Cassiopea*. (*B*) *Cassiopea* in its natural upside-down orientation. (*C*) Oral view of *Cassiopea*, asterisks indicate the position of radially spaced rhopalia. (*D*) For drop-test recordings, (d1) jellies are placed in an array of clear screen-bottom cylinders and raised to a set height, then allowed to acclimate for 5 min. The array is then dropped, forcing the animals into the water column, which they eventually respond to by pulsing towards the base (2). Recordings were made in infrared at 15 frames per second (CAM). (d2) The average pixel intensity is calculated every frame within a region of interest (example image series shown above the pulse trace) and normalized to remove background light fluctuations. The frames with the least intensity occur when the jelly is most contracted. An example time between contractions, the Inter-Pulse-Interval (IPI), is shown. (*E*) During the day (yellow) and the night (grey), on 8 jellyfish, across 4 d and nights, we cycled them through 8 to 10 rounds of 5 min for acclimation followed by a “drop,” measured their time to first pulse, and then quickly reset for a second drop to measure reversibility. The vertical red line is the normalized median of 3-IPIs predrop, the threshold between fast- and slow-pulsing animals. (e1) *Left* panel is the result after 5 min of acclimation, and the *Right* panel is the result after 2 min of acclimation. (e2) *Left* panel, box plots show the time to first pulse of day (yellow) or night (grey), and the *Right* panel, box plots show the time to first pulse of fast- vs slow-pulsing animals, considering whether they are 5 min (blue) or 2 min acclimated (orange). *P*-values calculated using one-way ANOVA, followed by Tukey HSD to adjust for multiple comparisons. (*F*) The behavioral recording paradigm is an initial recording (control 1), that includes 2 full nights and 2 full days, followed by a husbandry period for feeding and cleaning. Jellies are then returned to the recording setup for either 2 normal nights and 2 d of (control 2), or 2 nights of light-based sleep deprivation (LSD). LSD achieved using 5 min of light every 25 min, all night long. At the end of the recordings (ZT11, red bar and asterisk), animals have 3/4 of their ganglia amputated for RNAseq. For optimal RNA extraction, larger animals were used, ~6 cm diameter. (*G*) Average sleep in 30-min bins. control (blue), light-based sleep-deprived (LSD, orange), error bars are SEM; control, n = 10 (288 h), LSD, n = 7 (336 h). Red line and asterisk indicate the time of sampling for control and LSD conditions.

Sleep across animals is defined by three behavioral criteria: reversible quiescence, homeostasis, and latency-to-arousal (1). This definition has mostly been applied to animals with centralized nervous systems; however, recently, sleep has been characterized in the marine jellyfish *Cassiopea* (2), and the freshwater polyp, *Hydra* (3), so sleep predates the emergence of centralized nervous systems, and likely serves a function in neural homeostasis. *Cassiopea* naturally orient upside-down on the shallow ocean floor (Fig. 1*B*) and predominantly pulse in place through rhythmic contractions of the bell muscle to collect food, exchange gases, and support their symbiont (4). The pulsing behavior is led by ganglionic pacemakers that reside in the rhopalia (5, 6), which are radially spaced light- and balance-sensing organs (Fig. 1*C*). Cassiopea meet sleep criteria (1, 2, 7): 1) They display quiescence, a slower pulse rate at night, that is rapidly reversible upon stimulation, 2) they respond slower to stimulation during sleep, evidence of increased latency-to-arousal, and 3) if deprived of this nighttime quiescence they experience a rebound, a compensatory low activity period, the following day (homeostasis). Because the pacemakers in the ganglia control the pulsing behavior of the animal (2, 5, 6), the regulation of sleep must be integrated at these sites. Here, we exploit *Cassiopea*, its distributed neural architecture, and its position as an early-branching metazoan, to investigate the emergence of sleep-regulatory mechanisms during evolution.

## Sleep-Bout Characterization in *Cassiopea*

To associate specific gene expression patterns with sleep or wake, we defined a behavioral activity threshold to separate the two states. Previously, *Cassiopea* were shown to increase latency-to-arousal at night, as measured by their response to gently falling from a stationary position in the water (drop-test) (2). Here, we find that low-activity (slow-pulsing) is associated with increased latency-to-arousal (i.e a sleep-like state) and is reversible, meaning the quiescence is sleep rather than a coma-like state. Using a similar approach to that applied in other animals (3, 8–10): 1) we determined a nonaroused activity level (behavioral baseline), and then 2) measured the response time to an arousing stimulus (Fig. 1*D*). We take advantage of how *Cassiopea* briefly freeze pulsing after the drop-test, perhaps akin to the hyperarousal freeze response of other animals (11), to associate the activity predrop with the latency-to-arousal to determine the animal state (wake or sleep).

We took the same pulse-counting approach as was previously published (2), and with modifications, monitored pulsation, quantifying the interpulse interval (IPI) to calculate the average of 3-IPIs predropping in both the light and the dark (Fig. 1 *D*, *d*2 and *E*, *e*1, *Left* panel); this established a nonaroused light and dark behavioral baseline, normalized by the mean activity. Then, for step 2, we measured the latency-to-arousal after the drop-test by quantifying how long it took the animal to begin pulsing (time to first pulse) after the drop. After each 5 min acclimation and drop we repeated the test within 2 min to determine reversibility (Fig. 1 *E*, *e*1). We found that slow pulsing animals (IPI>median), in both the dark and the light, had an increased latency-to-arousal for the initial test (Fig. 1 *E*, *e*2, *Right* panel, blue), but not upon repetition (Fig. 1 *E*, *e*2, *Right* panel, orange), indicating this test is identifying a reversible quiescent state (sleep). After 5 min animals acclimate, but during the 2 min postdrop arousal period, IPI is not indicative of their state, as both slow- and fast-pulsing animals respond quickly to the drop-test (Fig. 1*E*). Together, these experiments show the slow steady-state pulsing activity in *Cassiopea* is indicative of a sleep state, so we moved on to investigate their regulatory response to nighttime sleep-deprivation.

## Homeostatic Response to Sleep-Deprivation in *Cassiopea* Shows Differential Expression of Genes Similar to Those Implicated in Vertebrate Sleep in Addition to Lineage Specific Genes

The molecular basis of sleep homeostasis is not well understood (12–15), nor is there much knowledge of its conservation across animals (7). To target sleep homeostasis, we initially developed a light-based sleep-deprivation (LSD) method, giving the animals 5 min pulses of light every 25 min during the night. We carried out a series of behavioral recordings on six animals, three control and three LSD, and quantified the IPI (Fig. 1 *D*, *d*2 and *F* and *SI Appendix*, Fig. S1*A*). We defined a sleep–wake threshold (normalized median IPI) from each 24-hour recording for each animal following the above method, giving us the amount of sleep in 30-min bins. Control animals slept little during the day, except for a brief mid-day siesta, and sleep ~70% of the night (Fig. 1*G* and *SI Appendix*, Fig. S1*B*). LSD animals showed a strong homeostatic rebound (compensatory low activity) during the day, and slept little at night (Fig. 1*G* and *SI Appendix*, Fig. S1*B*).

**Fig. 2.**
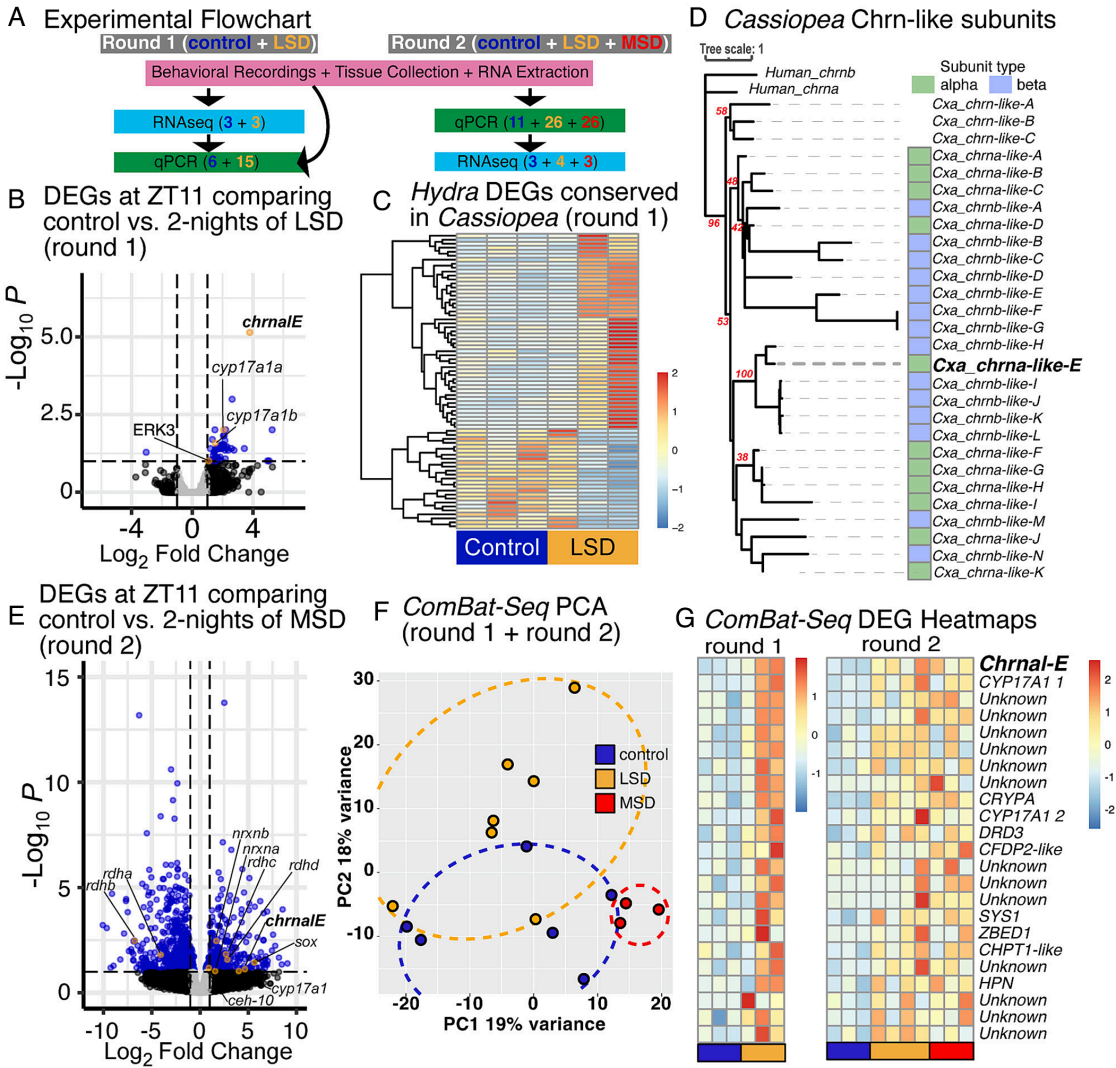
RNAseq reveals sleep-related DEGs. (*A*) Experimental Flowchart depicting Round 1 and Round 2 of RNAseq, of control (blue), light sleep deprived (LSD, gold) and mechanically sleep deprived (MSD, red). In Round 1 and Round 2 we conducted behavioral recordings. Colored numberes are the n-value screened per condition. In Round 1, *chrnalE* was detected as highly differentially expressed, and then qPCR was used to screen more animals. In Round 2, we added the MSD category, and *chrnalE* was used to determine which animals went for RNAseq. (*B*) Volcano plot visualizing log_2_ fold-change (x-axis) and log_10_ *P*-adjusted value (y-axis) of gene expression from a pairwise comparison between LSD treated jellyfish and control jellyfish. Dotted lines show a *P*-adjusted cutoff of 0.1 and a fold-change cutoff of ±1. Genes meeting these thresholds are shown with a blue dot, with key genes highlighted in orange with labels. (*C*) Heatmap of regularized log transformed gene expression of sleep-related genes conserved between *Hydra* and *Cassiopea* (3). (*D*) Phylogenetic analysis of known and putative Chrns. Maximum likelihood phylogeny generated in IQ tree. Bootstrap support in red. (*E*) Volcano plot visualizing gene expression from a pairwise comparison between MSD treated jellyfish and control jellyfish. Dotted lines show a *P*-adjusted cutoff of 0.1 and a fold-change cutoff of ±1. Genes meeting these thresholds are shown with a blue dot, with key genes highlighted in orange with labels. (*F*) Principal component analysis of ComBat-Seq normalized expression data for meta-RNAseq analysis, which combined multiple datasets capturing LSD and MSD treated animals and their associated controls from two separate experiments. Samples largely clustered according to their treatment. Colored ellipses were manually applied to highlight clusters. RNAseq samples in blue are controls, orange are LSD animals, and red are MSD. (*G*) Heatmap of regularized log transformed gene expression for the twenty-four differentially expressed genes determined with DEseq2 using the ComBat-Seq normalized reads analysis in the LSD vs control (round 1) dataset and the MSD vs control (round 2) dataset. Genes are listed according to the *P*-adjusted values calculated with DESeq2.

We compared the gene expression profiles of animals in the late day (ZT11) that had either experienced control conditions or had been exposed to LSD for two consecutive nights, to attempt to measure a regulatory response to sleep deprivation and detect sleep-related genes (Figs. 1*F* and 2*A* and *SI Appendix*, Fig. S1*B*). Importantly, LSD animals at ZT11 had experienced the daytime rebound sleep associated with nighttime sleep deprivation (Fig. 1*G*). Several core neuroactive receptors, and many components of GABAergic, circadian, and cholinergic pathways were represented in the transcriptome (*SI Appendix*, Fig. S1 *A*–*D*). Principal component analysis clustered control animals together with one LSD animal, and the two other LSD animals clustered apart, suggesting variation in individual response to LSD (*SI Appendix*, Fig. S1 *D*, *d*1).

Nevertheless, compared to controls, sleep deprived animals had 62 up-regulated genes and 1 down-regulated gene (Fig. 2*B*) (*SI Appendix*, Table S1). Of the 63 differentially expressed genes, approximately half were unknown in function, potentially with lineage specific roles in regulating sleep. Using sleep-associated *Hydra* genes (3) to prioritize *Cassiopea* orthologs, we found clear differences between LSD samples and controls, although one LSD treated sample more closely resembled control patterns of gene expression (Fig. 2*C* and *SI Appendix*, Fig. S1 *D*, *d*1). Differentially expressed genes included those associated with sleep deprivation from other organisms, including ERK3, cAMP-responsive element-binding protein (CREB), EGF-like protein, steroid-metabolizing enzymes, and a neuronal acetylcholine receptor (Fig. 2*A*). Additionally, functionally annotated genes that were not previously associated with sleep included epithelial splicing regulatory protein, matrix metalloproteinases, as well as a hemicentin, although their functional role, if any, in sleep will require further investigation. A gene most closely resembling a neuronal acetylcholine receptor was one of the most up-regulated genes in response to LSD. We also analyzed KEGG enrichment and found seventeen enriched pathways, including sleep-related pathways “cholinergic synapse” and “NOD-like receptor signaling pathway,” as well as immune response pathways “TNF signaling pathway,” “NF-kappa B signaling pathway,” and “IL-17 signaling pathway” (*SI Appendix*, Table S2).

Given the general importance of cholinergic systems in neural function and sleep homeostasis in other animals (12, 14–20), we chose to explore the cholinergic pathway further. *Cassiopea* has 25 orthologs of acetylcholine receptor-like genes (Fig. 2*D*), which can be categorized by the presence of a well-characterized Cys–Cys pair ligand binding site, as well as the presence of a C-loop (21). We phylogenetically identified 11 alpha subunits (we named chrnal-A-K) and 14 beta subunits (we named chrnbl-A-N) of cholinergic receptors in the *Cassiopea* genome, named so as not to confuse them with the established subunits (Fig. 2*D*). The differentially expressed ortholog (*chrnal-E*) was identified as an alpha subunit (*SI Appendix*, Fig. S1*F*).

## Mechanical Sleep Deprivation Also Induces *chrnal*-E Expression

To determine whether mechanical stimuli result in gene expression responses in *Cassiopea* similar to light stimuli, we repeated the RNAseq experiment using mechanical sleep deprivation (MSD). After optimizing flow, containment, and imaging for this condition, we found animals mechanically sleep deprived using pulses of water during the night, 1 min every 4 min, exhibited significant rebound sleep during the day (Fig. 1*G* and *SI Appendix*, Fig. S1*B*). Given the variable gene expression response to LSD, we repeated the light sleep deprivation experiment, and selected animals from LSD and MSD based on their *chrnal-E* expression (Fig. 2*A* and *SI Appendix*, Fig. S1 *E*, *e*1). MSD animals exhibited a greater number of differentially expressed genes (353 up-regulated, 600 down-regulated; *P*-adjusted = 0.1) (Fig. 2*E* and *SI Appendix*, Table S3). As with the first experiment, individual response to MSD varied, despite careful sample collection (*SI Appendix*, Fig. S1 *E*, *e*1). As expected, *chrnal-E* was up-regulated (fourfold Log2) in sleep deprived animals (Fig. 2*E*), as well as a *neurexin*, a second acetylcholine receptor (chrnbl-J), steroid-metabolizing enzymes, a series of development genes (*homeobox*, *craniofacial development protein*, *sox*), and genes involved in retinoid metabolism (*retinol dehydrogenases*). Among the genes that were down-regulated in MSD animals, we found a glutamate receptor and melatonin receptor, both genes strongly implicated in sleep regulation. Enrichment analysis identified pathways involved in neural regulation (“Axon guidance,” “Axon regeneration,” and “Synaptic vesicle cycle”) (*SI Appendix*, Table S4). In analyzing the top 100 most differentially up- and down-regulated genes in the MSD condition, we found only ~1/3 were annotated with a known function in either case. Of the functionally annotated genes, most have not been previously implicated in sleep regulation, excluding *chrnal-E*. For example, a putative hemicentin and a tumor necrosis factor alpha-induced protein are up-regulated 10-fold, but like many genes, further functional characterization is necessary to determine whether they play a central role in *Cassiopea* sleep or homeostasis.

In order to analyze the multiple RNAseq datasets, we conducted a meta-analysis using Combat-Seq, which adjusts raw RNAseq counts by modeling batch effects between multiple datasets using a negative binomial regression (22). We assessed both Round 1 and Round 2, which included a second set of LSD animals. Correcting for batch effect with *ComBat-Seq,* samples from control, LSD, and MSD animals each clustered closely within a PCA space (Fig. 2*F* and *SI Appendix*, Fig. S1 *E*, *e*2). Using conservative cutoffs, we recovered 23 genes that showed consistent expression differences between control and sleep deprived animals.

In sleep deprived animals (LSD and MSD treated), *Chrnal-E* emerged as the top-most differentially expressed gene. Among the list of genes were steroid alpha-hydroxylase/lyase-like which has been associated with circadian regulation (23), a craniofacial development protein, as well as a dopamine receptor (*SI Appendix*, Table S5). Although many of the genes were not differentially expressed when the two experiments were analyzed separately, the 23 genes from Round 1 and Round 2 reveal clear patterns of up-regulation in SD animals (Fig. 2*G*). All reported sequencing data has been made publicly available on NCBI (24). While all of these genes warrant further investigation, these data strongly suggest *chrnal-E* plays a key role in regulating sleep or homeostasis in *Cassiopea*.

## Conserved Receptor Structure and Response to Cholinergic Modulators in *Cassiopea*

**Fig. 3.**
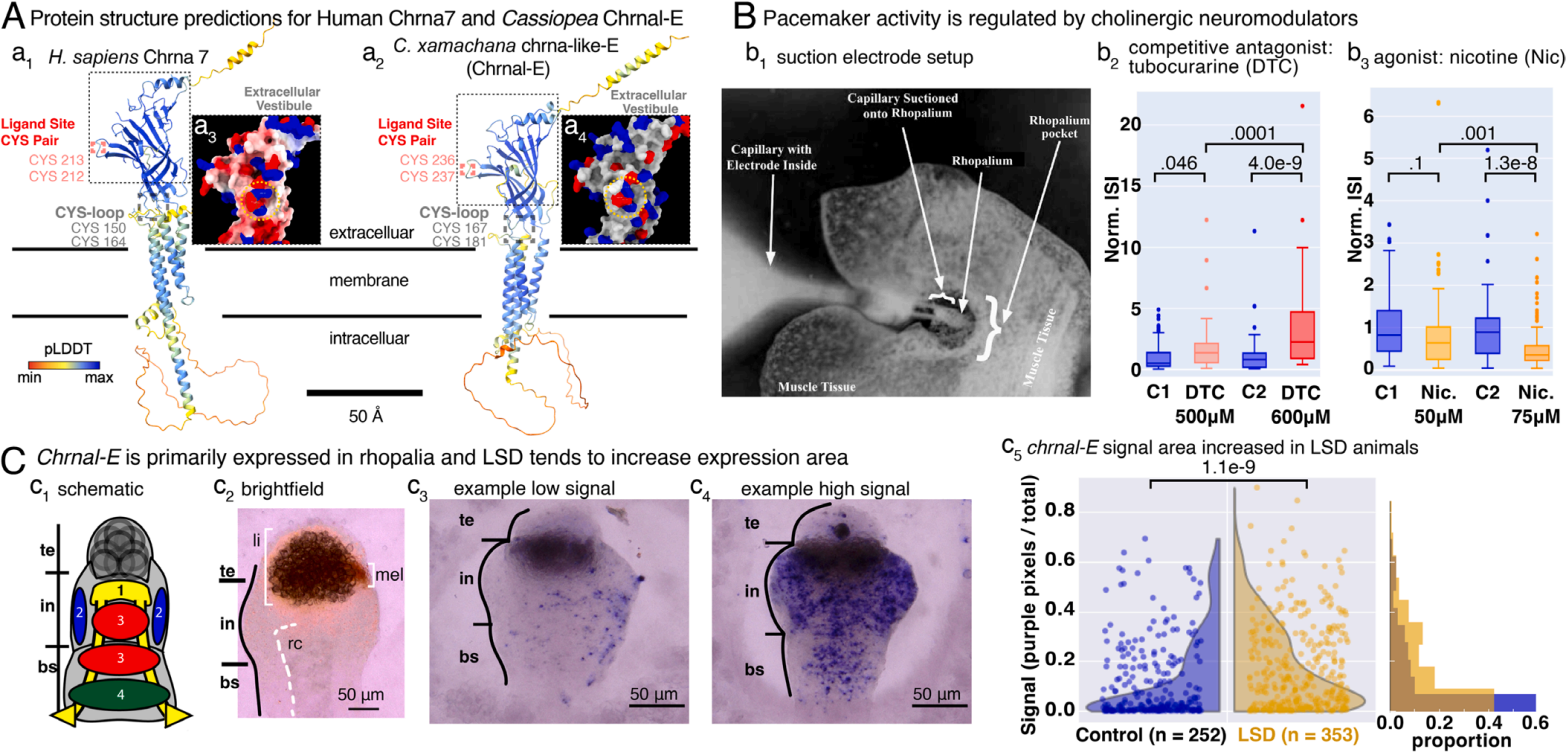
Evidence of cholinergic regulation in the nervous system of C*assiopea* (*A*) AlphaFold protein structure predictions for (a_1_) Human Chrna-7, and (a_2_) *C. xamachana* chrma-like-E (Chrnal-E), with labeled ligand site and CYS-loop indicating alpha-subunit similarity. pLDDT color legend indicates degree of structural confidence. The charged residues in the extracellular vestibule that regulate calcium permeation (25) for (a_3_) Human Chrna-7, and (a_4_) Chrnal-E. Color is charge, red (negative), and blue (positive). (*B*) Electrophysiology: rhopalia were suctioned into glass capillary electrodes (b_1_) and action potentials (APs) were recorded, and the inter-spike interval (ISI) was calculated. Explants recorded in artificial seawater (control, blue) versus artificial seawater with dissolved (b_2_) tubocurarine at 500 μM or 600 μM (salmon), a competitive antagonist against nicotinic acetylcholine receptors or dissolved nicotine at (b_3_) 50 μM or 75 μM (orange), an antagonist for nicotinic acetylcholine receptors. *P*-value calculated using One-way ANOVA followed by Tukey HSD to adjust for multiple comparisons. (*C*) *chrnal-E* expression shifts towards greater ganglionic expression when sleep deprived. (c_1_) Schematic of scyphozoan rhopalium ganglionic neural groups adapted from *Aurelia* (26). te, terminal, in, intermediate, and bs, basal segments. (c_2_) Brightfield image of a rhopalium, oral view, with the three labeled segments. The side half of the radial canal, rc, is marked by a white dotted line, which brings circulation to the rhopalium. The dark brown lithocyst, li, is in the terminal segment, and the light brown melanin, mel, of the spot ocelli on the aboral side is situated between the intermediate segment and terminal segment. Expression pattern of *chrnal-E*, with examples of low (c_3_), and high- (c_4_) expression, and their respective signal quantification. (c_5_) Quantification of ganglionic staining; ganglia are selected from images, and the ratio of purple pixels to total pixels in the ganglia was found. *Left* panel: violin plot of the distribution; *Right* panel: normalized histogram. control, n = 252, SD, n = 353. *P*-value calculated using Kolmogorov–Smirnov test.

Protein sequence analysis with BLASTp indicated *Cassiopea chrnal-E* as having high similarity to Chrna7, 9, and 10-like belonging to other cnidarian species (e.g., *Rhopilema esculentum, Clytia hemisphaerica, Hydractinia symbiolongicarpus*) across multiple classes. Cnidarian Chrna-like genes show striking structural and domain similarity with Human Chrnas (Fig. 3*A*). In particular, the structure gives insight into potential functional similarity, as the charged residues in the extracellular vestibule that regulate calcium permeation in Human Chrna7 (25) are present in only Chrnal-E (Fig. 3 *A*, *a*1 and *a*2) out of all *Cassiopea* Chrna-like subunits.

The cholinergic system is critical to sleep–wake regulation in worms, flies, fish, mice, and humans (16–20). Human Chrna7 is associated with memory and Alzheimer’s disease, which are both strongly linked to sleep (21, 27), and Chrns tightly regulate sleep in *Drosophila* (16, 20). Recently, cholinergic receptors were found to have neural and nonneural roles in the cnidarian anemone *Nematostella* (28). Additionally, cholinergic pacemaker neurons in a freshwater cnidarian *Hydra* were characterized (29) as similar to the pacemaker neurons in the vertebrate gut (30). However, the cholinergic system has yet to be implicated in sleep regulation of prebilaterian lineages (3), so we were driven to investigate the role of *chrnal-E* in regulating sleep in *Cassiopea*.

While cholinergic receptors are present, based on RNAseq and mRNA localization (see below), they may not control neurological activity, or may use an alternative neurotransmitter from acetylcholine. While acetylcholinesterase, used to break down acetylcholine, has been detected in *Actinia equina, C. hemisphaerica, Nematostella* (31–33), and in *Cassiopea* (*SI Appendix*, Fig. S1*D*), neither acetylcholine nor choline acetyltransferase, which synthesizes acetylcholine, have been documented in cnidarians. However, isolated nematocytes of *Nematostella vectensis* show acetylcholine-evoked outward currents that were abolished by nicotinic acetylcholine receptor antagonists and recapitulated by nicotine (33). Similarly, we used suction electrode electrophysiology on amputated ganglia (Fig. 3 *B*, *b*1 and *SI Appendix*, Fig. S1 *G* and *H*, *Materials and Methods*) to measure the time between action potentials (interspike interval) in the presence of nicotinic acetylcholine receptor (nAChR) effectors. We bathed the amputated ganglia with tubocurarine (DTC), a competitive antagonist, or nicotine (Nic), a highly membrane permeable agonist. In a dose-dependent manner, tubocurarine increased the interspike interval (Fig. 3 *B*, *b*2; 500 μM, *P*-value 0.046; 600 μM *P*-value, 4.0e−9, DTC 500 μM < DTC 600 μM, *P*-value 0.0001) while nicotine decreased the interspike interval (Fig. 3 *B*, *b*3; 50 μM, *P*-value 0.1; 75 μM *P*-value, 1.3e−8, Nic. 50 μM > Nic. 75 μM, *P*-value 0.001). These chemicals act on nAChRs at the site of the ligand binding, so we consider these data strong evidence of acetylcholine (or something like it) acting as a neurotransmitter in *Cassiopea*.

## *Chrnal-E* Is Primarily Expressed in Ganglia and Transcript Localization Area Increased due to LSD

We also confirmed that *chrnal-E* is expressed in the rhopalia. Rhopalia are divided into three segments (26): terminal, intermediate, and basal (Fig. 3 *C*, *c*1). The terminal segment includes the lithocyst for balance, and the indentation between the terminal and intermediate segment contains the pigmented-spot ocelli on the aboral side, with its brown melanin pigment. The basal segment connects the rhopalium to the body of the animal. We validated our in situ hybridization protocol by testing probes against RFamide and minicollagen (*SI Appendix*, Fig. S2 *A* and *B*). *Chrnal-E* localizes to the oral side of ganglia (Fig. 3 *C*, *c*3 and *c*4), predominantly in the intermediate and basal segments, but without a singular discrete pattern. There is considerable variability in mRNA expression patterns, both in levels and in ganglionic regions, even between the ganglia of a single animal (*SI Appendix*, Fig. S2*D*). Subcellular localization of *chrnal-E* mRNA appears nonnuclear and asymmetrical within the cytosol (*SI Appendix*, Fig. S2*E*).

Using in situ hybridization allowed us to localize *chrnal-E* expression, and though the intensity of the color is not necessarily indicative of expression level, we did strive to understand whether LSD caused a difference in *chrnal-E* pattern. We selected the rhopalium from each in situ image, pooled the images, and measured the ratio of the purple pixels to the total number of pixels in each rhopalium (*SI Appendix*, Fig. S2*C*). We found a significant shift toward broader *chrnal-E* expression in LSD ganglia (Fig. 3 *C*, *c*5, *P*-value 1.1e−9). Because of its association with ganglia and its change in response to LSD, we were motivated to ask whether Chrnal-E is functionally involved in regulating sleep.

## RNAi Knockdown Reveals a Functional Role for Chrnal-E in Promoting Wake in *Cassiopea*

**Fig. 4.**
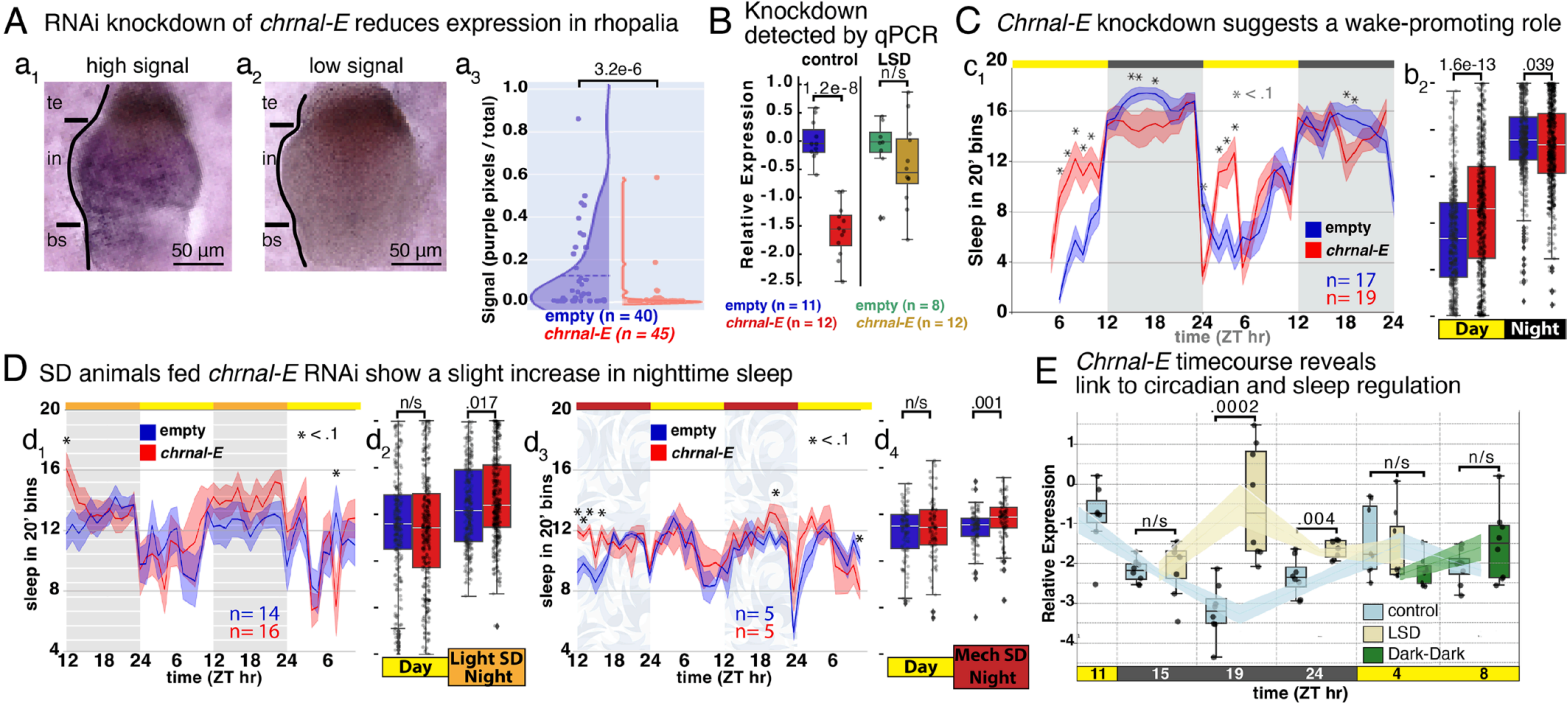
RNAi knockdown and nighttime expression supports a wake-promoting role for chrnal-E. (*A*) In situ hybridization characterization of *chrnal-E* in animals fed RNAi empty-vector (5 animals) or *chrnal-E* (5 animals) for 2 wk; (a_1_) high and (a_2_) low, *chrnal-E* expression area. (a_3_) Purple-pixel quantification of ganglionic staining. *P*-value calculated by One-way ANOVA. (*B*) Knockdown detected by qPCR, normalized to B-tubulin and B-actin. (*C* and *D*) Sleep in 20’ bins, showing the average sleep of animals fed empty-vector (blue) or *chrnal-E* (red), for control (b_1,2_), LSD (d_1,2_), MSD (d_3,4_). Sleep per hour and (b_1_, d_1_, and d_3_) sleep day vs. night (b_2_, d_2_, and d_4_). (*E*) Expression of *chrnal-E* comparing controls, LSD, and Dark-Dark (animals kept in the dark) animals from dockside field samples. *P*-values generated by One-way ANOVA, and Tukey’s HSD was applied in the case of multiple comparisons. All line plots include SEM.

We optimized RNAi conditions based on previous work (34–38) (*SI Appendix*, Figs. S3 *A*–*F* and S4) and tested whether the *chrnal-E* knockdown affects sleep behavior (*SI Appendix*, Fig. S3 *G* and *H*). We inserted 387 bp of *chrnal-E* into the L4440 vector. As a control, we used empty vector, or 427 bp of *minicollagen* (*mcol*), which we considered unlikely to affect sleep behavior. For characterization of the knockdown, five animals were fed the *chrnal-E* vector, and five animals were fed the empty vector, for 2 wk. Staining of the *chrnal-E* knockdown rhopalia revealed reduction in expression area of the *chrnal-E* mRNA (Fig. 4*A*, *P*-value 3.3e–6).

Next, we recorded pulsing behavior for 3 wk, during which the animals were fed RNAi vectors, and finally, at ZT 11, tissue was extracted and the expression level of *chrnal-E* quantified. We detected a strong reduction in *chrnal-E* expression in animals fed *chrnal-E*-RNAi (*SI Appendix*, Fig. S3 *G* and *H*). Behaviorally, by the 2 wk, animals fed *chrnal-E* vector began displaying increased daytime sleep and decreased nighttime sleep compared to empty-vector RNAi (Fig. 4*B*) or *mcol*-vector (*SI Appendix*, Fig. S3 *G* and *H*). If it were sleep promoting, we would see less sleep at night in the knockdown, but if it were wake promoting, we would see more sleep during the day in the knockdown. Although we see some decrease in sleep at night, the strong increase in daytime sleep supports a wake promoting role for chrnal-E. This pattern resembles that in mouse wake-promoting histaminergic neurons in the tuberomammillary nucleus; when *Bmal1* is deleted, mice spend more time awake at night than their littermate control mice, and less time awake during the day (39). When we quantified *chrnal-E* expression in LSD animals at ZT11, we did not find a significant knockdown (Fig. 4*C*); however, animals fed *chrnal-E* RNAi vector did show a slight increase in nighttime sleep compared to controls (Fig. 4*D*), indicating *chrnal-E* may promote stimuli-induced exogenous arousal. Together, these findings indicate Chrnal-E plays a key role in promoting wakefulness.

## Expression of *chrnal-E* Appears Circadian and Increases in Response to Sleep Pressure

Because of the RNAi results, coupled with the previously noted variability in *chrnal-E* expression detected at ZT11, we hypothesized that *chrnal-E* expression is dynamic. To address this, we sampled many wild *Cassiopea* at circadian timepoints starting at the end of the day (ZT11) through to the morning (ZT8) and measured *chrnal-E* expression using qPCR (Fig. 4*E*). Expression of *chrnal-E* in control animals decreased at night and increased again in the morning (ZT4) (Fig. 4*E*), relative to *b-tubulin* and *b-actin,* indicating it may be regulated by light, may be connected to the circadian cycle, or may be linked to the sleep homeostat. Strikingly, animals that experienced 7 h of LSD showed a threefold increase in *chrnal-E* expression in the middle of the night (ZT19) compared to controls (Fig. 4*E*, *P*-value 0.0002), showing that prolonged nighttime wakefulness causes *chrnal-E* expression to spike. Levels of *chrnal-E* also remain elevated relative to controls at the end of the night (Fig. 4*E*, ZT24, *P*-value 0.004), but return to control levels by morning. Therefore, we propose that *chrnal-E* homeostatically responds to prolonged stimulus-induced exogenous arousal to support or promote wake. This homeostatic mechanism occurs over a few hours, presumably to ensure that the change in activity is prolonged before the system compensates and may be an opportunity to study homeostatic mechanisms on multiple timescales (40, 41). If *chrnal-E* were instead a light-response gene, we would expect expression to quickly change and then stabilize in each light condition, similar to the photoinducible and rhythmic clock genes *Period 1 and 2* (42).

To begin to address whether *chrnal-E* is light or circadian regulated, we placed several animals in a dark tank, so that in the morning we could sample from animals that did not experience the night-day transition. Previously, *Cassiopea* were shown to display circadian pulsing behavior (2). We found *chrnal-E* expression to be similar among all three conditions during the morning (Fig. 4*E*, ZT4-ZT8, *P*-values n/s), rather than maintaining the lower nighttime level of expression, indicating that *chrnal-E* may be under circadian regulation. Also, because the sleep deprived animals were rebounding in the morning, but *chrnal-E* is not distinguishable from controls (Fig. 4*E*, ZT4, *P*-values n/s), we predict other members of the sleep regulatory system drive the rebound behavior.

## Discussion: Regulation of Sleep in *Cassiopea*

The molecular mechanism of sleep and homeostasis remains a subject of intense research in the sleep field (43, 44). We endeavored to uncover components of the sleep homeostat in *Cassiopea.* Cnidarians are one of the earliest branching animal lineages, and with sleep being intertwined with numerous processes, we expected sleep deprivation to cause changes in expression to both novel and conserved genes. We found that sleep deprived *Cassiopea* tend to change expression of a nAChR alpha subunit-like gene (Fig. 2), *chrnal-E,* similar to those expressed in the centralized nervous systems of Bilateria. Together, the knockdown data and circadian time course suggest that chrnal-E promotes wakefulness under both circadian and homeostatic regulation. Evidence for circadian regulation comes from the control and dark–dark conditions (Fig. 4*E*), while homeostatic regulation is supported by the observation that *chrnal-E* expression increases only after several hours of nighttime arousal (Fig. 4*E*). However, *chrnal-E* is not itself a member of the homeostat, as it does not appear to regulate rebound sleep (Fig. 4 *D* and *E*), rather it is strongly expressed in response to increased sleep drive.

We recognize, however, that the differential expression observed does not appear in every animal, or every ganglion (Fig. 3 and *SI Appendix*, Fig. S2*D*). While there may be technical explanations, cnidarians also exhibit variability in response to environmental stimuli at the individual animal level for metamorphosis (45), self-repair (46), regeneration (47) and the behavioral response to sleep deprivation (2) (Fig. 1*G* and *SI Appendix*, Fig. S1*B*). Importantly, *chrnal-E* is dynamically expressed (Fig. 4*E*), and the variability at ZT11 may be due to sampling time and differences in recovery rates. Also, the pacemaker activity is variable between the ganglia and perhaps this influences gene expression. While the prevalent changes provide insight into sleep regulation, the animals that do not respond as anticipated may yet be compensating in an unknown way. Additionally, though our evidence suggests Chrnal-E promotes wakefulness, its strong knockdown does not lead to constant sleep, indicating that Chrnal-E is just one component of the sleep regulatory system. It is important to also remember this is still early days for RNAi in *Cassiopea*. Time and technical advances may lead to improved knockdown and greater effect.

Phylogenetic analysis of cnidarian Chrns showed they grouped together, separate from those in Bilateria, and the addition of *Cassiopea* subunits did not particularly alter previous phylogenetic structure (27), supporting the notion that Chrns independently radiated in cnidarian and bilaterian lineages. However, there is some debate as to whether the presence of Chrns is evidence of a true cholinergic system. Our readout for their behavioral state is their pulsing activity, which is controlled by pacemaker cells in the ganglia, and because *chrnal-E* mRNA is also present in the ganglia, we hypothesized a neural regulatory function for *chrnal-E*. Indeed, tracking pacemaker generated action potentials (APs) in the presence of cholinergic modulators supported our finding, as tubocurarine decreased AP frequency and nicotine increased AP frequency, a competitive antagonist and agonist, respectively (Fig. 3*B*). However, we do not yet know whether acetylcholine is the neurotransmitter being used in *Cassiopea*, so more work is required to fully define the circuit. Together with previous reports (28, 29, 31–33), this work supports a behavior regulating role for nAChR-like genes in Cnidaria that hints to the presence of a cholinergic system with a role in sleep regulation.

To know where *chrnal-E* lies in the sleep regulatory pathway will require more investigation. As a point of reference, all 13 AChRs in *Drosophila* have been systematically investigated using CRISPR, and sleep and arousal are regulated by distinct acetylcholine receptors in different neuronal types (20). While two nicotinic AChRs promote endogenous sleep, through octopaminergic neurons, another promotes stimulus-induced exogenous arousal, through dopaminergic neurons, suggesting a delicate regulation of sleep by AChRs. Chrnal-E could act through dopaminergic neurons to promote wake, or interact with the circadian clock, among other scenarios (20, 39, 42, 44). It is also possible that *chrnal-E*, though not acutely induced by light, is instead phase shifted by light, rather than prolonged wakefulness, so additional work is needed to determine the exact relationship between *chrnal-E*, light, and the clock. There is also evidence for cell-autonomous sleep-regulatory mechanisms of a nonneuronal nature in mammals and *Drosophila* (7): tissue metabolism, growth, or oxidative stress could drive sleep, so perhaps *chrnal-E* acts downstream of a stress sensor.

Finally, the mechanism by which *chrnal-E* influences activity is unknown, but its structural similarity to nAChRs with high calcium permeability offer potential insight (25). Neurons detect changes in their own firing rates through a set of calcium-dependent sensors that then regulate receptor trafficking (40). More work is required to determine whether Chrnal-E influences neural activity and behavioral state by changing calcium permeability. Either way, we are excited by how RNAi opens the door to further studies that should resolve sleep-regulatory mechanisms and the extent to which there is conservation or lineage specific novelty in *Cassiopea*. To date, there are numerous examples of conserved regulation of sleep, and with evidence indicating some conserved functions of sleep, our findings support there being a conserved form of sleep in the Cnidarian–Bilaterian ancestor and further implies a deep importance of the cholinergic system in the evolution of discrete sleep and wake states.

## Materials and Methods

### Cassiopea Husbandry.

Animals were maintained in a circulating system with 28 to 34 ppt artificial seawater. They were fed brine shrimp eggs (E-Z Egg, brineshrimpdirect) using an eye dropper, daily, directly to their oral arms. Ephyrae were maintained in isolated boxes floating in the husbandry system, to keep them safe. Quarter sized or larger animals were kept in retrofitted 10-gallon *Xenopus* tanks with standpipes that drain into the sump. Inside the sump were two 4-lbs bags of ceramic bioballs (JIH), a UV sterilizer (COODIA), and two heaters (Cobalt Aquatics), to maintain the system temperature at 84 °F. An auto top-off system was used to maintain salinity (Useek), which pumps water from a distilled water storage tank into the sump. To maintain ~150 PAR throughout the tanks, we used three 165 W LED aquarium lights (VIPARSPECTRA) to cover 6 tanks. Polyps were maintained at room temperature in a dark cabinet and fed live hatched brine shrimp once every other day.

### Pulse Tracker.

#### Recording setup and approach.

Animals ~2 inch in diameter were used for behavioral recordings prior to **RNA sequencing**. The recording setup is also a circulating system of repurposed 10-gallon tanks, however, the bottom was removed and replaced with a 6-inch x 12-inch × ¼-inch piece of acrylic, kept in place with RTV Silicone Sealant (CRC). Animals were placed in watch glasses (Eisco™ Watch Glasses), which were affixed to the acrylic using silicone grease. The edge of the watch glasses was ringed with ¼ inch clear silicone tubing pieces ~1 inch long, to act as a bumper. A standpipe cut to ~ 1-inch sets the water height so that it flows into the watch glasses, but the jellies cannot escape. One 165 W LED aquarium light (VIPARSPECTRA) was placed laterally, and four infrared lights were placed angled from above to illuminate the jellyfish. Two to four watch glasses were placed in each tank depending on their size, which is calibrated to the animal. For **RNAi behavioral experiments,** square watch glasses of 1 and ⅝ inch (Carolina Biological Supply) were placed in an acrylic tray with holes for water exchange, with each watch glass holding one jellyfish. White netting was placed above and to the sides of the jellyfish to prevent mixing animals during the recording, and to ensure that the jellyfish remained within their watch glass. ACA1300-200UM Basler cameras with 10X Computer macro lens with BP850 IR filter were used. Cameras were placed about 1.5 feet beneath the ganglia tracker recording setup. MATLAB and a custom script were used to run the cameras, which were set to record for 60 min,15 frames per second mp4 videos, one per hour.

#### Light and mechanical based sleep deprivation.

Using an Arduino microcontroller, running a “Iot Relay Enclosed High-power Power Relay for Arduino, Raspberry Pi” (Digital Loggers), we programmatically ran bright LEDs for light-based sleep deprivation, or the water pumps that jet water. For light-based sleep deprivation, the lights turn on for 5 min every 25 min, so a total of 10 min per hour. For mechanical sleep deprivation, the water turns on for 1 min every 4 min, so a total of 12 min per hour. Depending on the size of the animal, whether it is for RNAseq or RNAi, animals are placed on smaller or larger watch glasses (Eisco™ Watch Glasses). For mechanical sleep deprivation, we found it necessary to switch off the infrared lights, so nothing is filmed, for the 1 min of water pulsing, because the video compression would break when the pixels were changing due to bubbles and light scattering. However, the moment the pumps turn off, the infrared lights turn back on, this is easily achieved by plugging the infrared lights into the “normally on” position of the lot relay and plugging the pumps in the “normally off” position. For RNAi experiments, mechanical sleep deprivation was achieved using water flowing from directly above the animals, using an array of waterspouts. Sleep deprivation of smaller animals appeared less consistent, perhaps stronger light, higher flow, or alternative means would be required to reach a similar level as what can be achieved with larger jellies.

#### Drop-test video and analysis.

Drop-tests require more space, and 3x2 array of clear cylinders with a taut brine-shrimp screen were placed in a pulse tracker recording setup, but with the standpipe level set high to allow space for the cylinders to rest and drop. The array had arms that rested on small boxes that keep the cylinder array about 2 inches from the top of the water. To drop the array, the small boxes that rest on the lip of the plastic tank were quickly pulled out, and the array falls and stops when the array arms land on the plastic tank rim. The IPI-post drop, in both the light and dark, was assessed by eye from the video recordings, because as the cylinders dropped, they disturbed the water and made computer analysis complicated, though by eye it was still very easy to see. The IPIs of all predrop test were run-through normal video analysis, generating a normalization factor by which all IPIs are then divided. A median threshold (on normalized predrop IPIs in both light and dark), sorts wake-like from sleep-like normalized postdrop IPIs (Fig. 1).

#### Video analysis.

To analyze pulse rate of jellyfish, recorded videos were processed with the program FFmpeg (48), using custom parallelized version, ffmpeg_parallel.sh. This converts multiple videos into image stacks simultaneously at a rate of 15 fps. Pulse Tracker Analysis Software (2) can be used on any length recording period, for control vs SD RNAseq experiments we analyzed the full recording and used larger animals with ~6 cm diameter. For RNAi experiments there were more animals, and they had ~3 cm diameter. For RNAi, we analyzed 20 min of every hour, equivalent to 18,000 images, throughout the experiment as was done previously (2). There are two main steps in the Pulse Tracker Analysis Software. First, Pixel Intensity Extractor takes the first image of the first time period and allows the user to select a rectangular region of interest surrounding the jellyfish. Then, inside of this region, the software measures average pixel intensity of each frame of that period. Second, Peak Finder, normalizes the output from step one, and then detects local minima that correspond with muscle contractions. When the intensity passes a chosen threshold value a pulse is demarcated, indicated with a red dot (*SI Appendix*, Fig. S1 *A*, *a*2 and *a*3). The output to this program has the time at which a contraction occurred and the IPI between the current and previous pulse. Ideal Peak Finder (*SI Appendix*, Fig. S1 *A*, *a*2) vs problematic Peak Finder (*SI Appendix*, Fig. S1 *A*, *a*3), where the average pixel intensity change was not significant enough to allow for clear pulse detection. The low-quality graphs can occur due to insufficient pixel change difference when the jellyfish pulsed, which often was caused by lighting fluctuations surrounding the jellyfish independent of pulse behavior, or the animal managed to turn sideways. We did not have issues with the larger animals in the RNAseq experiment but did have some issues with the smaller animals for RNAi. We attempted to choose 20-minute periods with good pulse quality and dropped time points from individuals if no satisfactory 20 min period could be found.

### Confocal Imaging.

Images were taken with a Zeiss LSM 880 with a confocal microscope at 10×, 20×, and 40× magnification, or using a Zeiss LSM 700. Image processing (Z projection, color display) and quantifications were done with Fiji software.

### In Situ Hybridization.

#### Probe Synthesis.

Plasmids were extracted from colonies containing PCR-derived sequences of interest. 3 to 5 μg of plasmid DNA were digested using select unique cutting restriction enzymes; NcoI, NotI, or SphI were used with SP6 polymerase while SpeI was used for T7 polymerase to synthesize DIG-labeled RNA probes.

#### In situ hybridization.

Our rhopalia specimens were stained by in situ hybridization using a protocol adapted from *Nematostella* (49). Briefly, animals were anesthetized in 800 μM menthol. Animals were first fixed in 4% PFA, 0.02% Tween-20, 0.3% glutaraldehyde, in PBS, pH 7.2. Fixation times varied by probe. For *rfamide, chrna,* and *mitf* we used 30 min, *minicollagen* required 40 min. Fixations were gently rocked at room temperature. After the first fixation, specimens were washed in PBS, and then permeabilized and depigmented through 25, 50, 75, and 100% ethanol and back to PBS, with 5 min per stage, with gentle rocking. Secondary fixation used 4% PFA in PBS, pH 7.2, bringing the total fixation time to 1 h per probe. Specimens were washed several times, and then treated with Proteinase K (50 μg/mL) in 0.1% Tween-20 PBS (PTw), for 23 min. After stopping the reaction with glycine, specimens were washed then treated with 1% triethanolamine (TEA), 1% TEA + 3 μ/mL acetic anhydride, 1% TEA + 6 μ/mL acetic anhydride, and then washed in PTw. Specimens were refixed in 4% PFA for 1 h, and washed in PTw. Samples were then transferred to hybridization buffer, first washing at room temperature, then brought to 65 °C, for 24 h, for prehybridization. Probes (at 1 μg/mL) were denatured at 80 to 90 °C for 5 min in hyb buffer, and samples in the hybridization mix were returned to 65 °C for 48 h. After retrieving probes, which can be reused, samples were washed in descending concentrations of hyb. buffer mixed with 2x SSC pH 7.0, down to 25% hyb. buffer and 75% 2X SSC, in 30 min steps, and returning samples to 65 °C. After 100% 2X SSC washes, samples were washed in descending concentrations of 0.02X SSC down to 25% 0.02X SSC, 75% PTw, and finally 100% PTw, initially at 65 °C, then at room temperature. Samples were then transferred to Roche Blocking Buffer, and placed on a rocker at 4 °C overnight. Anti-Digoxigenin was diluted in Roche Blocking Buffer 1:5,000, and returned to 4 °C overnight. After several washes in 1% Triton-X100 in PBS, samples were transferred to AP buffer with MgCl_2_. Then samples were transferred to AP buffer without MgCl_2_, and then finally into the color reaction, 3.3 μL/mL NBT (50 mg/mL) and 3.3 μL/mL BCIP (50 mg/mL), or Fastred. Reactions were kept in the dark, but monitored, and generally took 3 to 5 h at room temperature to complete. Samples were then washed in PTw and fixed for 1 h in 4% PFA in PBS at room temperature and stored in PBS at 4 °C for several months without issue.

#### In situ hybridization controls.

*Cassiopea* rfamide mRNA staining was consistent with the antibody staining with localization to the ganglia and sensory nerve net (50), in medusae, and in the tentacles and mouth of the polyp (*SI Appendix*, Fig. S2 *B*, *b*1 and *b*2). In a contrasting control, nematocyst-specific gene *minicollagen* (*mcol*) mRNA stain showed the classic salt-and-pepper pattern in the oral side of the bell, and the tentacles and body of the polyp (*SI Appendix*, Fig. S2 *B*, *b*3 and *b*4), similar to *Nematostella* (51).

#### Imaging and quantification.

Specimens were mounted on glass slides, or in agarose-mold dishes, and imaged on a Keyence VHX-5000 digital microscope. As described in *SI Appendix*, Fig. S2*C*, images were cropped such that only the rhopalia remained, and then everything but the purple pixels were masked, and the area of the purple pixels relative to the size of the whole rhopalium was calculated and reported as *signal*. Special attention was paid to optimizing the purple pixel thresholds to avoid including brown pixels associated with melanin pigmentation, and white balancing was done on those rhopalia that had nonspecific purple background from the color reaction.

### Immunohistochemistry.

Medusae or amputated ganglia were fixed in 4% paraformaldehyde for 2 h at room temperature (20 to 25 °C), and washed in PBS. To remove pigment from *Symbiodinium microadriaticum*, a dinoflagellate algal symbiont of Cassiopea, samples were dehydrated and rehydrated in an ethanol series (50, 70, 90, 100, 100, 90, 70, 50) for 5-minute intervals followed by a final wash in PBS. For samples stained with 488-Phalloidin, isopropanol substituted for ethanol for 30 s intervals. Samples were blocked in PBS containing 10% CAS-Block for 2 h. Primary antibodies FMRFamide (EMD Millipore AB15348; rabbit, 1:1,500), or acetylated Tubulin (Sigma-Aldrich MFCD00164512; mouse, 1:200), were diluted in 10% CAS-Block, and samples were incubated at 4 °C with gentle rotation overnight. The sample was washed with PBS, then placed in secondary antibody mixtures, depending on the primary antibody, Alexa Fluor 488 (mouse, 1:200), Alexa Fluor 555 (rabbit, 1:200), dilutes in 10% CAS-Block, and incubated in the dark at 4 °C for 12 h. The sample was washed PBS, and nuclei stained using 1ug/ml Hoechst 33258 (ThermoFisher 62249) for 2 h.

### RNA Extraction and Quantification.

#### RNA extraction.

For RNAseq, 3/4 of the rhopalia from each animal (approximately 12 per animal) were pooled for each sequencing sample. For qPCR, 6 rhopalia were amputated, and 2 rhopalia were pooled per animal in 3 separate extractions. After amputation, specimens were transferred into Stellar Scientific Deadbolt 1.5 mL tubes, and were immediately flash frozen in liquid nitrogen and stored in a –80 °C freezer. For extraction, tubes were seated on a liquid-nitrogen-cooled mortar and were homogenized physically with a cooled pestle (Thomas Scientific). The Plant Spectrum Total RNA kit (Sigma) was used to isolate the RNA from the homogenized specimens. Ground tissues were immediately vortexed in 1 mL of Lysis buffer, double the amount specified by the kit. After material from the binding solution was applied to the columns, they were treated with a DNase digestion (Sigma). When needed, samples were additionally cleaned and concentrated using the Zymo Research RNA Clean and Concentration Kit and eluted into 13 μL. Once concentrated, samples of RNA were analyzed with an Agilent Bioanalyzer system to assess yield and integrity.

### RNA Sequencing and Differential Gene Analysis.

Paired-end 150 bp RNA sequencing at the UC Berkeley sequencing core facility used the Illumina NovaSeq 6000. Reads were trimmed and quality filtered using Trimmomatic (version 0.39) with default settings. Trimmed reads were aligned to the gene model predictions of the *Cassiopea* genome version 0.2 (52), accessed through the Joint Genome Institute genome resource portal (https://mycocosm.jgi.doe.gov/Casxa1/Casxa1.home.html) using STAR (version 2.5.3a) with the following settings: --limitOutSJcollapsed 10,00,000 --limitSjdbInsertNsj 10,00,000 --outFilterMultimapNmax 100 --outFilterMismatchNmax 33 --outFilterMismatchNoverLmax 0.3 --seedSearchStartLmax 12 --alignSJoverhangMin 15 --alignEndsType Local --outFilterMatchNminOverLread 0 --outFilterScoreMinOverLread 0.3 --winAnchorMultimapNmax 50 --alignSJDBoverhangMin 3. DESeq2 was used to analyze differential gene expression (53). The Wald test was used for hypothesis testing, with FDR cut-off of 0.05. The data were regularized log transformed for visualization of the differential expression analysis results. To identify a universal sleep deprivation response in gene expression of LSD and MSD animals, raw reads from experiment 1 and experiment 2 were batch corrected using ComBat-Seq function in the Bioconductor package sva version 3.42.0 (22). Batch corrected counts were used as input for differential expression analysis using DEseq2 as described above.

### Gene Identification.

Primarily using *Nematostella* and *Clytia*, genes of interest were BLASTed against the *Cassiopea* transcriptome, and hits then reciprocally BLAST for secondary confirmation. Domain analysis used HMMER (54). Phylogenetic analysis used phylemon2 (55), and trees were constructed using maximum likelihood analysis (MLA) from protein sequences.

### Phylogenetic Analysis.

38 putative nAChR genes were predicted in the Cassiopea genome. A maximum-likelihood protein alignment of putative nAChRs used MAFFT (version 7.429) and manually filtered following previously described criteria (28). Sequences were checked for the C-loop domain, with cysteine residues separated by 13 amino acid residues. Sequences lacking the motif were excluded from further analysis. Sequencing with over 75% of the alignment absent from the TM domain were also excluded. After filtering, 24 protein coding sequences were retained. Sequences with a cysteine–cysteine pair prior to the transmembrane domains were classified as alpha subunits. Phylogenetic reconstruction of putative Cassiopea AChR used iqtree (v2.0.3) with 2,000 ultrafast bootstrap replications.

Protein sequence alignment of nAChR subunits used MAFFT (version 7.429) with default settings. Sequences were retrieved from NCBI, except for *Nemopilema nomurai* (56) and *Clytia hemisphaerica* (57), which are available through the respective data repositories. Downloaded sequences shorter than 390 bp were removed prior to alignment. Alignments were trimmed using BMGE (version 1.2) with default settings. Reconstruction of a maximum likelihood phylogenetic tree used IQ-TREE (version 2.2) (58), and model selection used ModelFinder (59). Phylogenetic reconstruction used ultrafast bootstrap approximation with 2,000 replicates.

### Cloning.

#### cDNA synthesis.

cDNA was prepared using Superscript IV Reverse transcriptase and RNA samples of at least 100 ng.

#### qPCR primer validation and analysis.

qPCR used a Bio-Rad CFX Real-Time Machine. We used cDNA samples made from >200 ng of RNA to assess primer efficiency. Serial cDNA dilutions of 40, 20, 10, 5, 2.5, and 1.25% were prepared. cDNA and primers for the genes of interest were added to reactions with SsoAdvanced Universal SYBR Green supermixes and were prepared in duplicates for Standard Curve and Relative Quantification experiments. After validation, for every sample, *bactin*, *btubulin*, *mcol*, and *chrnal-E* were amplified. A minimum of three animals were used in each comparison, with three biological samples taken from each animal, and three technical replicates were used in every qPCR run. qPCR results were analyzed following standard qPCR calculations and error propagation (60).

### RNAi Procedure in Cassiopea.

#### RNAi vector assembly and induction.

The RNAi construct synthesis and induction is based on techniques employed in the planarian *Schmidtea mediterranea* (34), and the nematode *C. elegans* (35), the freshwater polyp *Hydra Vulgaris* (36). As shown in *SI Appendix*, Fig. S4, to generate RNAi constructs, sequences (~300 to 900 bp) from the genes of interest were inserted between the T7 promoters of the ampicillin-resistant, double T7 L4440 vector, and these recombinant vectors were transformed into HT115(DE3) *Escherichia coli* (tetracycline-resistant, rnc14::ΔTn10, containing lacUV5 promoter-T7 polymerase). An overnight culture of L4440 in HT115(DE3) (grown in LB with 100 ug/mL ampicillin and 12.5 ug/mL tetracycline) was diluted 1:100 in 2xYT (100 ug/mL ampicillin) and incubated at 37 °C until the OD_600nm_ = 0.4. To induce the culture, isopropyl β-D-1-thiogalactopyranoside (IPTG) was added to a final concentration of 1 mM before a 5-h incubation at 37 °C. 2 ml of the culture was pelleted, the supernatant removed, and then stored at –80 °C.

#### RNAi cocktail prep.

Decapsulated brine shrimp eggs (Brine Shrimp Direct) were pipetted into a 1.5 mL tubes, and using a tabletop centrifuge briefly spun down, and the excess water was removed leaving approximately 300 μL of brine shrimp eggs. Eggs were crushed with a pestle using an electric tissue homogenizer, or a ceramic mortar and pestle, until the eggs no longer made a cracking sound. Following complete emulsification, we added 1 μL drop of blue food coloring, and 50uL of 2% PEG with dissolved blue chalk, in order to visualize the food inside of the jellyfish. We then added 400 μL of 0.8% low melting point agarose dissolved in Sterile Filtered Artificial Sea Water (SFASW) to the emulsified eggs. We transferred this egg mixture to its respective bacterial pellet; bacteria pellets are set to 125 μL (if pellet is too large or small it is adjusted). We then transferred this to a respective extrusion syringe, which correlated with each different treatment, with brine shrimp netting securely fastened to the tip. The filled apparatus was left to solidify on ice. We rapidly compressed the syringe to extrude the mixture into a 1.5 mL tube, then the mixture was diluted 1:1 in SFASW. RNAi cocktail was stored in 4 °C for up to 1 wk. We found the blue dye is not necessary once people are comfortable with the feeding, and it is best to not use it.

#### RNAi feeding schedule.

Every day at approximately 1PM (Zeitgeber hour 6) the jellyfish received 50 to 100 μL of the RNAi cocktail. For the first time we ran this experiment, the jellies were moved between the husbandry system in an isolated container, and the recording setup. In this case, if it was on a recording day, we removed the jellyfish from the recording setup prior to feeding and placed them in their respective weekday cubbies in a husbandry setup, and if not on a recording day we fed them directly in their weekday cubbies. For all the data in Fig. 4, animals were consistently in the circulating husbandry tank receiving RNAi until the end of the 2 wk, when they were transferred to the recording tank system, where again they were fed every day. We used a P200 pipette to slowly extrude the RNAi cocktail onto the oral arms of the jellyfish, and to verify eating we could visualize the blue food inside of the mouths of the jellyfish. The jellyfish were allowed 1 h to eat, and then the excess food and mucus was removed.

### Electrophysiology.

#### Ganglia dissection and mounting.

Jellyfish (approximately 3 to 5 cm in diameter with similar average pulse rates) were chosen and placed oral-side up in a sterile petri dish containing sterile filtered artificial seawater (SFASW). Dissections were performed on the perimeter of the bell to isolate two adjacent ganglia from the whole animal. Anesthetic agents such as menthol were not used during the ganglia dissections since previous experiments showed an inconsistent activity post treatment with administering of other treatments. After dissection, the tissue was placed under a light microscope at 50% illumination. Simultaneously, 5 mm glass electrodes were pulled using a Sutter® Model P-87 Flaming/Brown Micropipette Puller and cut with tweezers to fit the approximate size of the ganglion’s circumference under the microscope.

#### Extracellular recordings.

Extracellular recordings were performed using an A-M SystemsTM Model 1800 2-Channel Microelectrode AC amplifier and an A-M SystemsTM Bipolar Suction Electrode, connected by an A-M SystemsTM Model 1800 Headstage. The amplifier settings were set to 1,000× amplification, 10 Hz low cut-off filter, 5 kHz high cut-off filter, no capacitance, and recording mode. Recordings were performed in a Faraday Cage covered by an electroneutral shield which was grounded with the amplifier. A reference wire in the SFASW and recording wire in the glass capillary were used to filter noise and record electrical activity. Under a light microscope, ganglia from the tissue preparation were suctioned in SFASW under 28 °C perfusion into the glass capillary with the recording wire. Ganglia were checked for intactness and complete suction under the microscope lens. Voltage recordings were conducted on the Windows Whole Cell Program (WinWCP) for 4 min 26 s with a sampling interval of 0.08 ms.

#### Nicotine and DTC treatments.

Control recordings were performed for 4 min 26 s under 50% illumination in SFASW. Immediately following the control recording, all SFASW was removed from the petri dish and 3 mL of treatment solution preheated to 28 °C was added with a micropipette. Treatment recordings were recorded immediately following the control recordings and for the same duration. Each treatment solution was diluted from a stock concentration in SFASW and stored at 4 °C. DTC concentrations of 0.6 mM and 0.775 mM and Nicotine concentrations of 50 μM and 75 μM were used.

#### Spike identification.

WinWCP recording files were converted to txt files to obtain voltages measured at all timepoints. Using linear modeling and principal component analysis in Python programming, action potential spikes were identified and extracted. Action potentials with a minimum voltage change of 40 mV, within 3 SD of the mean voltage, and a continuous waveform between 20 to 50 ms were used in analysis. All spikes with a depolarizing waveform or positive waveform were analyzed. Each spike was verified by eye during and after recordings before extraction was performed. Interspike intervals (ISI) were calculated using the timepoints at the end resting potential and beginning rest potential between two spikes.

## Supplementary Material

Appendix 01 (PDF)

## Data Availability

All sequences have been uploaded to NCBI under accession number PRJNA1250492 (24). All other data are included in the article and/or *SI Appendix*.
