# Supplementary material for: Sleeping upside-down: Knockdown of a sleep-associated gene induces daytime sleep in the jellyfish Cassiopea
Source: Proc Natl Acad Sci U S A. 2025 Jul 14;122(29):e2505074122. doi: 10.1073/pnas.2505074122 (PMC12305049; doi:10.1073/pnas.2505074122)
Supplement: Supplementary file 1 — Appendix 01 (PDF) [file pnas.2505074122.sapp.pdf]

`

PNAS

Supporting Information for:

Sleeping Upside-Down: Knockdown of a Sleep-Associated Gene Induces Daytime Sleep in the Jellyfish *Cassiopea*

Michael J. Abrams, Aki Ohdera, Diana A. Francis, Owen Donayre, Henry Chen, Kevin Lu, Celeste Y. Hsu, Hannah Zeigler, Richard M. Harland

Michael J. Abrams
Email: mjabrams@berkeley.edu

This PDF file includes:

Supplemental Figure 1
Supplemental Figure 2
Supplemental Figure 3
Supplemental Figure 4
Figure S1
Tables S1 to S5
SI References

`

Supplemental Figures

Supplemental Figure 1. Pulse Tracker recording and analysis, rhopalia amputation, and RNA quality control. (A) Pulse Tracker recording setup, **(a_1)** with full spectrum (FS) and infrared (IR) laterally angled lights and camera recording at 15 frames per second, fitted with an IR filter. **(a_2)** Mechanical sleep deprivation is achieved by pulsing water ~perpendicular to the jellyfish for 1 minute every 4 minutes, all night. The flow is angled slightly at a tangent, so the flow becomes a vortex, spinning the animal in circles rather than pushing it up against the edge. The flow rate is adjusted by a valve in light with the pump. **(a_3)** Code then finds the troughs demarcating contractions, and labels them with red dots if the peak is over a set threshold, in this case -0.5 of the normalized intensity, and exports files with the time of each contraction and the inter-pulse-interval (IPI). **(a_4)** Example of unusable pulse tracker data where there is not sufficient change in average pixel to clearly label contractions compared to noise from background. **(B)** Average sleep / hour of the day (yellow) and night (dark grey), for light based sleep deprivation (LSD, golden), and mechanical based sleep deprivation (MSD, red), for round1 and round 2 of the RNAseq experiments. p-values generated by One-way ANOVA. **(C)** Amputation of rhopalia and surround tissue, while avoiding thicker mesoglea-rich tissue. **(D)** Principle component analysis of RNAseq, samples plotted in PCA space for round 1 **(d_1)** and round 2 **(d_2)** of the RNA sequencing . **(E)** qPCR to confirm differential expression of *chrnal-E* at ZT11 in control, LSD, and MSD conditions. Numbers samples in **(e_1)** are the samples represted on the PCA in **(e_2).** Control (blue), LSD (orange) and MSD (red). p-values generated by One-way ANOVA. **(F)** Phylogenetic analysis of known and putative Chrns. Maximum likelihood phylogeny generated in IQ tree. Human seratonin receptor is used as out-group for our analysis. *C. xamachana* sequences are in blue text on tips of tree branches.**(G)** Reference wire and electrode suctioned onto rhopalium in a petri dish with temperature regulated sterile filtered seawater on a light microscope state. **(H)** Depolarizing extracellular waveform identified as spike, after amplification. **(I)** Longer electrophysiology recording showing a series of spikes.

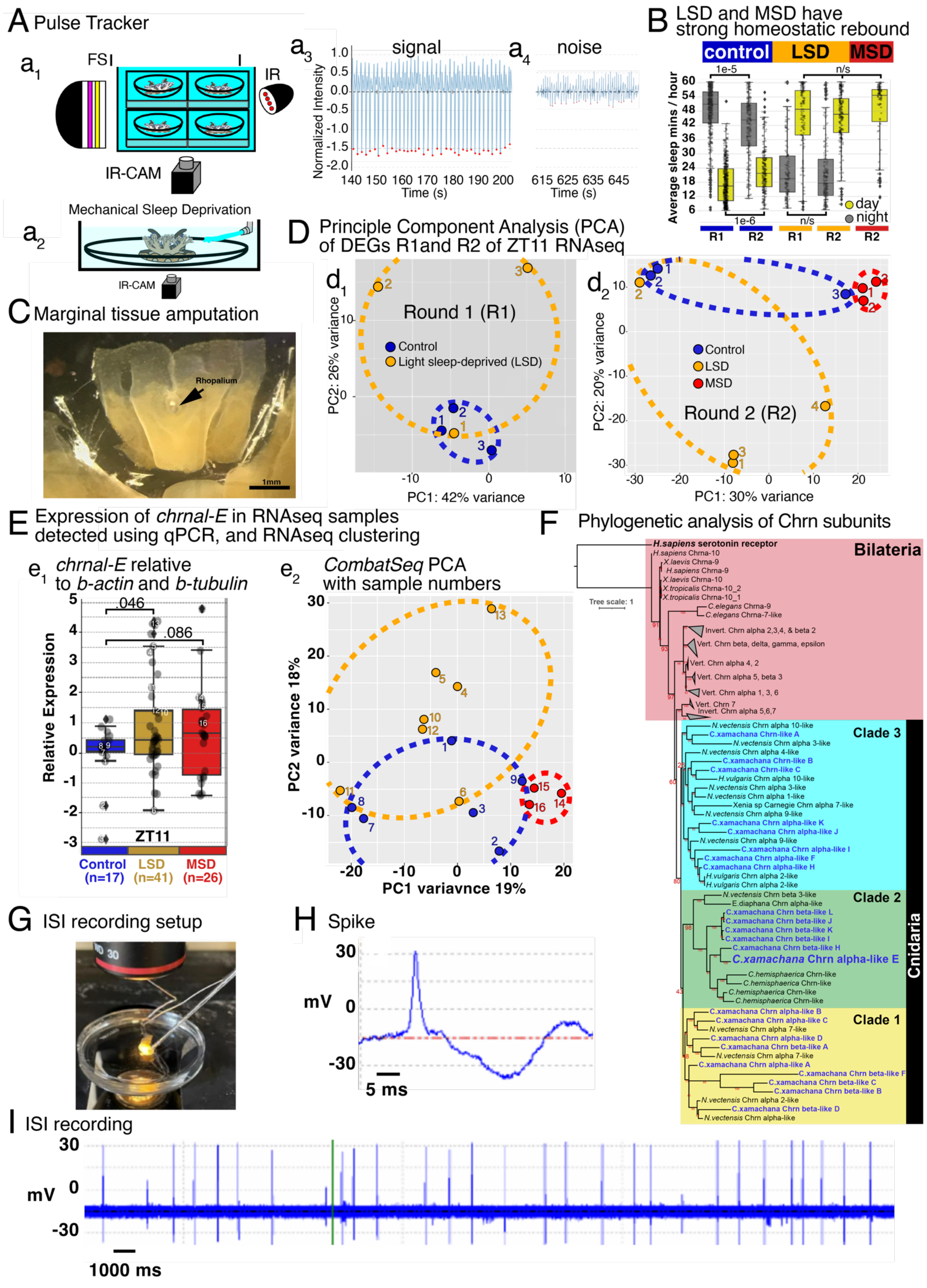

A Pulse Tracker
a1
FSI
IR
IR-CAM
Mechanical Sleep Deprivation
a2
IR-CAM
a3 signal
Normalized Intensity
Time (s)
a4 noise
Time (s)
B LSD and MSD have strong homeostatic rebound
control
LSD
MSD
Average sleep mins / hour
1e-5
n/s
1e-6
n/s
day
night
R1
R2
R1
R2
R2
C Marginal tissue amputation
Rhopalium
1mm
D Principle Component Analysis (PCA) of DEGs R1and R2 of ZT11 RNAseq
d1
Round 1 (R1)
Control
Light sleep-deprived (LSD)
PC2: 26% variance
PC1: 42% variance
d2
Control
LSD
MSD
Round 2 (R2)
PC2: 20% variance
PC1: 30% variance
E Expression of chrnal-E in RNAseq samples detected using qPCR, and RNAseq clustering
e1 chrnal-E relative to b-actin and b-tubulin
Relative Expression
.046
.086
ZT11
Control (n=17)
LSD (n=41)
MSD (n=26)
e2 CombatSeq PCA with sample numbers
PC2 variance 18%
PC1 variavnce 19%
F Phylogenetic analysis of Chrn subunits
Tree scale: 1
Bilateria
H.sapiens serotonin receptor
H.sapiens Chrna-10
X.laevis Chrna-9
H.sapiens Chrna-9
X.laevis Chrna-10
X.tropicalis Chrna-10_2
X.tropicalis Chrna-10_1
C.elegans Chrna-9
C.elegans Chrna-7-like
Invert. Chrn alpha 2,3,4, & beta 2
Vert. Chrn beta, delta, gamma, epsilon
Vert. Chrn alpha 4, 2
Vert. Chrn alpha 5, beta 3
Vert. Chrn alpha 1, 3, 6
Vert. Chrn 7
Invert. Chrn alpha 5,6,7
Clade 3
N.vectensis Chrn alpha 10-like
C.xamachana Chrn-like A
N.vectensis Chrn alpha 3-like
N.vectensis Chrn alpha 4-like
C.xamachana Chrn-like B
C.xamachana Chrn-like C
H.vulgaris Chrn alpha 10-like
N.vectensis Chrn alpha 3-like
N.vectensis Chrn alpha 1-like
Xenia sp Carnegie Chrn alpha 7-like
N.vectensis Chrn alpha 9-like
C.xamachana Chrn alpha-like K
C.xamachana Chrn alpha-like J
N.vectensis Chrn alpha 9-like
C.xamachana Chrn alpha-like I
C.xamachana Chrn alpha-like F
C.xamachana Chrn alpha-like H
H.vulgaris Chrn alpha 2-like
H.vulgaris Chrn alpha 2-like
Clade 2
N.vectensis Chrn beta 3-like
E.diaphana Chrn alpha-like
C.xamachana Chrn beta-like L
C.xamachana Chrn beta-like J
C.xamachana Chrn beta-like K
C.xamachana Chrn beta-like I
C.xamachana Chrn beta-like H
C.xamachana Chrn alpha-like E
C.hemisphaerica Chrn-like
C.hemisphaerica Chrn-like
C.hemisphaerica Chrn-like
C.hemisphaerica Chrn-like
Clade 1
C.xamachana Chrn alpha-like B
C.xamachana Chrn alpha-like C
N.vectensis Chrn alpha 7-like
C.xamachana Chrn alpha-like D
C.xamachana Chrn beta-like A
N.vectensis Chrn alpha 7-like
C.xamachana Chrn alpha-like A
C.xamachana Chrn beta-like F
C.xamachana Chrn beta-like C
C.xamachana Chrn beta-like B
N.vectensis Chrn alpha 2-like
C.xamachana Chrn beta-like D
N.vectensis Chrn alpha-like
Cnidaria
G ISI recording setup
H Spike
mV
5 ms
I ISI recording
mV
1000 ms

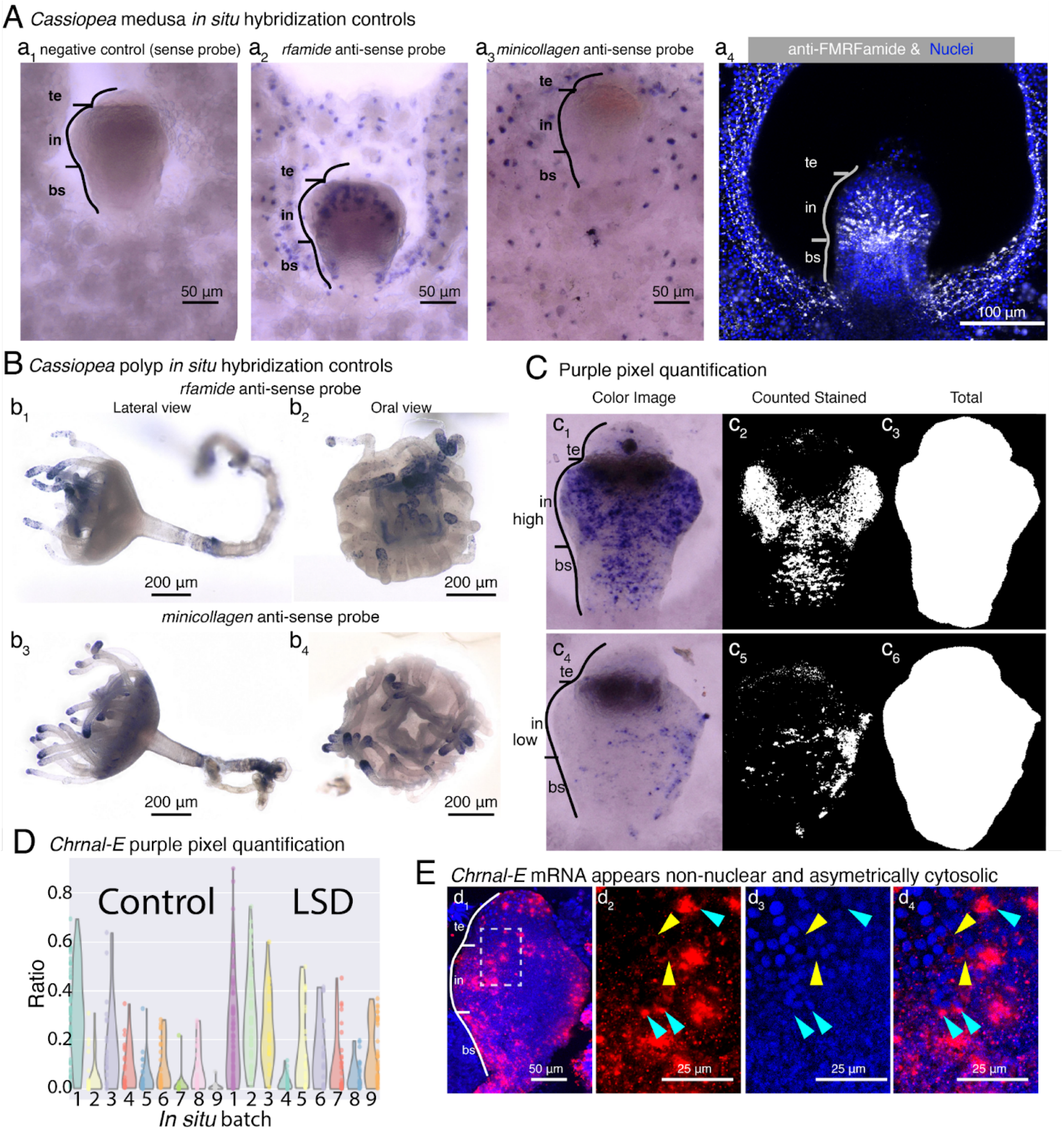

Supplemental Figure 2. *in situ* hybridization of *rfamide* and *minicollagen* in *Cassiopea* polyp and rhopalia, *chrnal-E* expression quantification and localization. (A) *in situ* validation in *Cassiopea.* **(a_1)** *Cassiopea* rhopalia show very low background staining with sense probes. **(a_2)** *rfamide* is highly expressed within the rhopalia and in the pocket that surrounds it, consistent with sensory nerve localization [31,46]. **(a_3)** *Minicollagen (mcol)* is not expressed in the rhoplalia, instead it has a salt-and-pepper pattern consistent with nematocyst localization. **(a_4)** FMRFamide antibody labels neurons in the diffuse nerve net and the ganglia, with a similar pattern to the *rfamide* anti-sense probe, and previous reports [31,46]. **(B)** *rfamide* and *mcol* expression patterns in polyps. **(b_1)** *rfamide*, and **(b_2)** *mcol*, localizing to the tentacles and around the mouth. However, *rfamide* is more dispersed in the tentacle and is less expressed in the body than *mcol.* **(C)** Purple pixel quantification of *chrnal-E* expression. **($c_{1,4}$)** Color images of chromogenic *in situ* hybridization. **($c_{2,5}$)** A mask for blue-purple pixels is generated. **($c_{3,6}$)** A mask for all pixels within the rhopalium is generated. Signal is the ratio of blue-purple pixels in rhopalium/ total pixels within the rhopalium. **D)** Quantification of staining per ganglion per ganglion per *in situ* batch, with the ratio of purple pixels to total pixels in the ganglion. **(E)** Fastred *in situ* hybridization of *chrnal-E* to visualize subcellular localization. **(e_1)** Fastred *chrnal-E* expression pattern on the oral side of a rhopalium. **(e_2)** Fastred *chrnal-E*, **(e_3)** Hoeschst nuclear stain, **(e_4)** merged. Expression of *chrnal-E* appears non-nuclear, and asymmetrically cytosolic. Yellow arrows indicate low levels and cyan arrows show high levels.

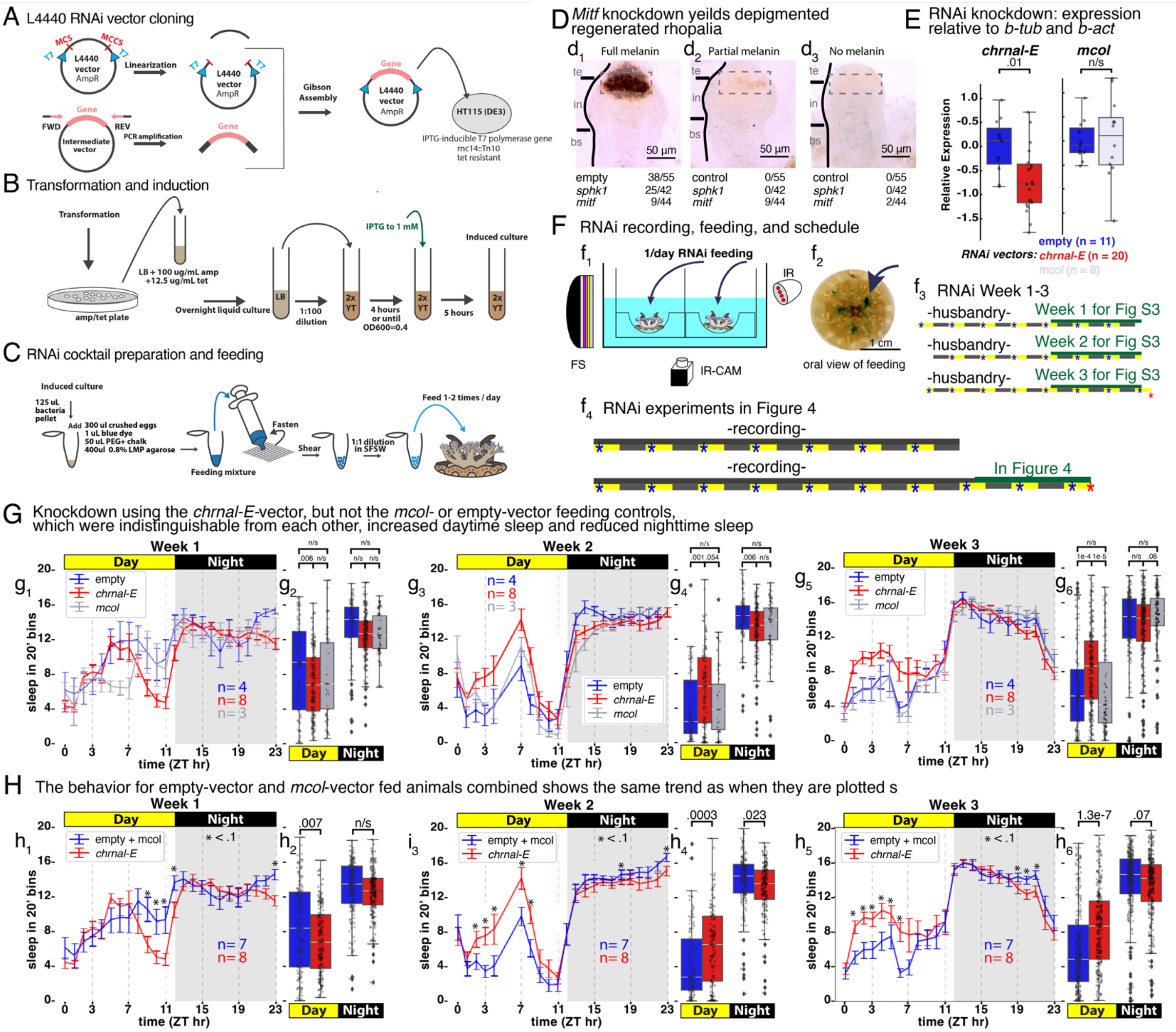

Supplemental Figure 3. RNAi vector cloning, induction, and preparation, and RNAi behavioral recording setup and group and RNAi knockdown of *mitf* and *chrnal-E*. (A) ~250-1000bp of target gene are amplified from cDNA and cloned into the L4440 vector, transformed into high efficiency DH5alpha, sequenced, then re-transformed into HT115 *E.coli.* **(B)** Induction using 1 mM IPTG in 2xYT media is accomplished in a series of grow-out steps. **(C)** Induced cultures are pelleted and stored at -80C. When needed, pellets are thawed, and mixed with 0.8% LMP, crushed brine shrimp eggs, PEG, and chalk, and extruded through a brine shrimp filter, and then diluted in sterile filtered artificial seawater. RNAi food can be kept at 4C for up to 1 week, feedings are once or twice a day, depending on the size of the animal and its tolerance of the cocktail. **(D)** RNAi vector containing 915bp of *mitf* was fed daily to three animals each condition for 21 days. All ganglia were removed after one week. After two weeks of recovery regeneration and melanin pigmentation was screened. There is a significant reduction in regeneration in animals fed *mitf* compared to empty (chi-square, p-value .017). Of the regenerated ganglia **(d_1)** only *mitf* animals had two additional phenotypes, **(d_2)** partial melanin, and **(d_3)** no melanin. **E)** qPCR of *mcol* and *chrnal-E*, relative to *beta-actin* and beta-*tubulin*, comparing expression in animals fed empty vector versus those fed *mcol* or *chrnal-E* vector. p-value calculated by One-way ANOVA. **(F)** RNAi is fed to animals in the Pulse-Tracker recording setup when doing behavioral recordings. Asterisk is approximate time of RNAi feeding. **(f_1)** RNAi is fed directly to the animals in the recording setup, **(f_2)** by placing RNAi-food directly on the tentacles, once or twice per day, where the animals have oral groves that bring the food to their manubrium. The recording timeline for data shown in this figure **(f_3)** and Figure 4 **(f_4). (G)** % sleep, day and night, for week1-3. Animals fed empty-vector (blue), mCOL-vector (orange), and ACHRa-vector (grey). Empty- and mCOL-vectors do not show significant differences in sleep at any time point in week 2 or week 3. Error bars are SEM. **(H)** Behavior from empty- and mcol-vectors are combined to generate a combined control behavior, to compare against the *chrnal-E* knockdown.

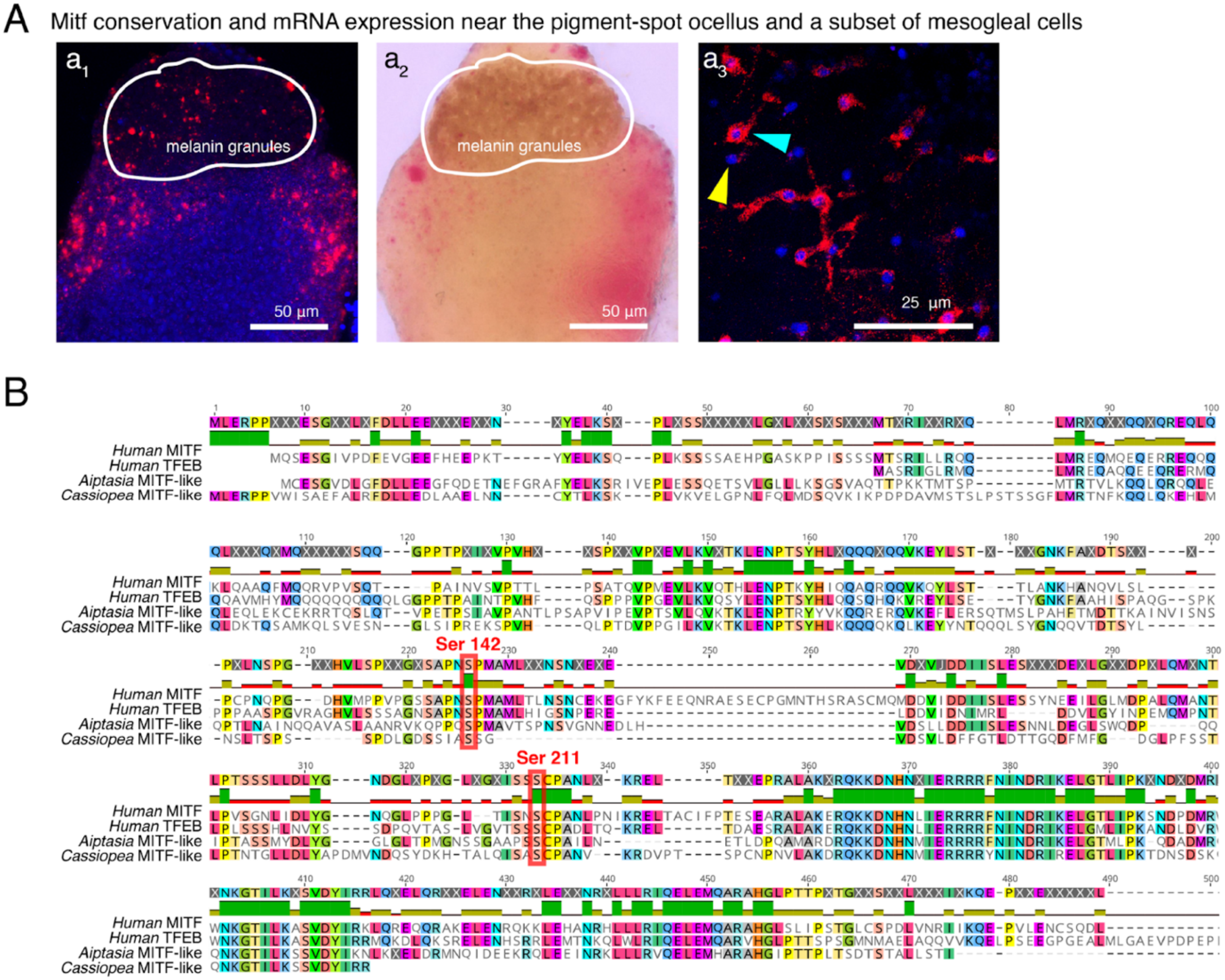

Supplemental Figure 4. *Mitf* expression localization and sequence conservation. (A) *Mitf* expression pattern in *Cassiopea.* **(a_1)** *Mitf* mRNA (red), is expressed flanking, and internal to, the region of melanin granules of the pigmented-spot ocellus **(a_2)**. **(a_3)** *mitf* mRNA also localizes to the cytoplasm of some cells inside the mesoglea, at high (cyan) and low (yellow) levels, compared to other mesogleal cells that appear just a nuclei (blue). **(B)** *Mitf* shares conservation of mTOR phosphorylation sites, Ser142 and Ser211, red boxes) and is therefore likely to be controlled by mTOR, as is the case with Human *mitf* [1,2].

Figure S1. KEGG pathway presence, absence. (A-D) KEGG pathways showing presence (tan-box)/absence (white-box) of (A) neuroactive ligand-receptor interactions, (B) GABAergic synapse, (C) circadian rhythm, and (D) cholinergic synapse.

A KEGG pathway neuroactive ligand-receptor interaction presence/absense

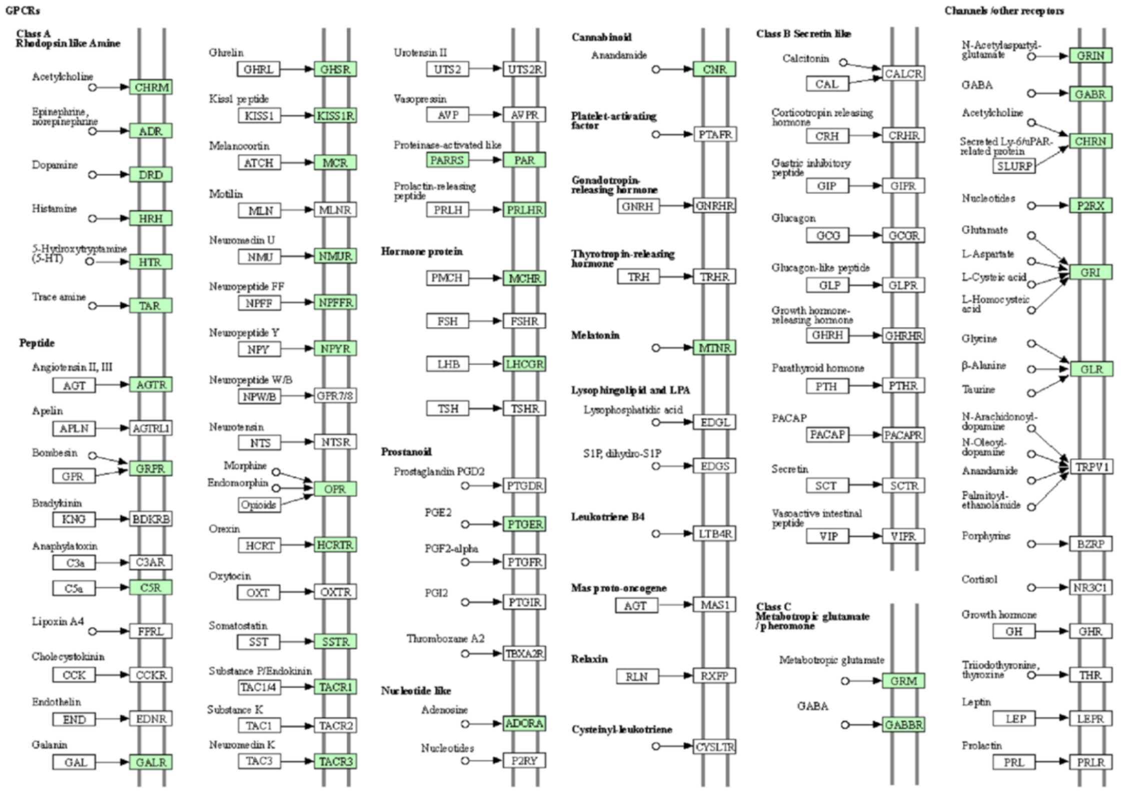

B KEGG Gabaergic synapse presence/absense

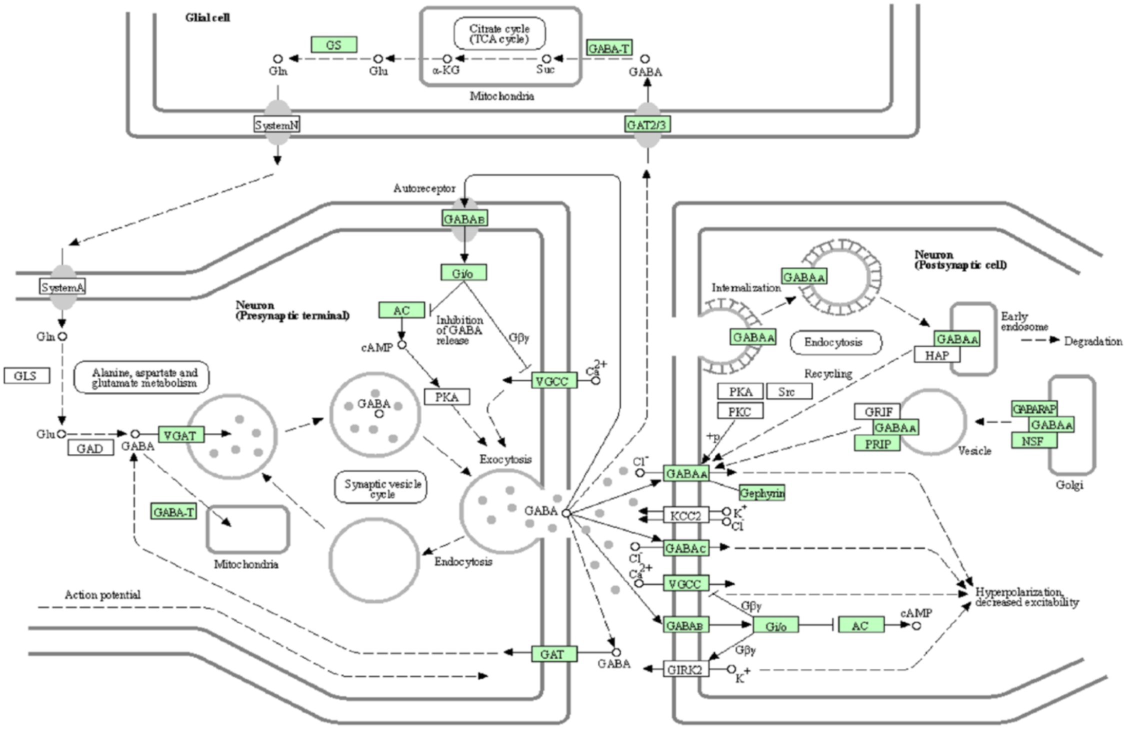

C KEGG circadian rhythm gene presence/absense

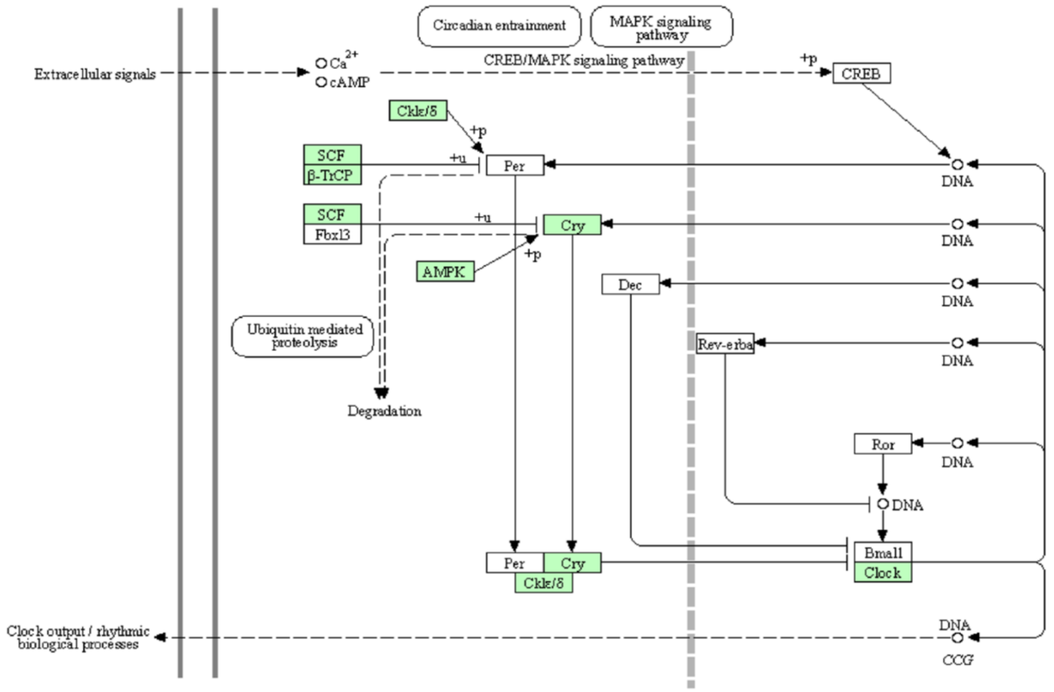

D KEGG cholinergic synapse presence/absense

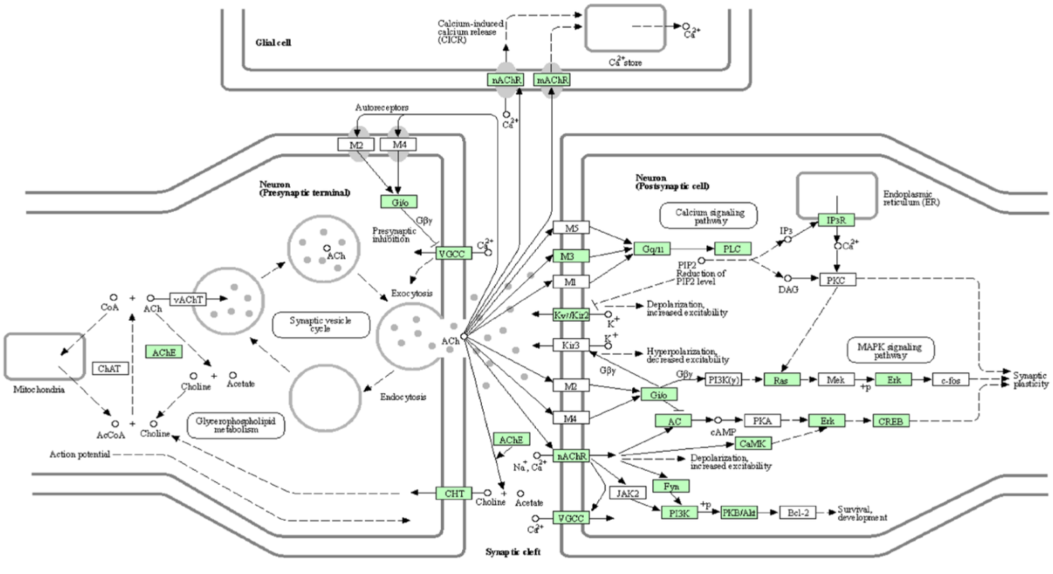

`

Supplemental Information References

1. Voss, P.A., Gornik, S.G., Jacobovitz, M.R., Rupp, S., Dörr, M., Maegele, I. and Guse, A., 2023. Host nutrient sensing is mediated by mTOR signaling in cnidarian-dinoflagellate symbiosis. *Current Biology*, *33*(17), pp.3634-3647.
2. Kozmik, Z., Ruzickova, J., Jonasova, K., Matsumoto, Y., Vopalensky, P., Kozmikova, I., Strnad, H., Kawamura, S., Piatigorsky, J., Paces, V. and Vlcek, C., 2008. Assembly of the cnidarian camera-type eye from vertebrate-like components. *Proceedings of the National Academy of Sciences*, *105*(26), pp.8989-8993.

Table S1. Differentially expression genes of animals in the late day (ZT11) that had either experienced control conditions or had been exposed to LSD for two consecutive nights

	baseMean	log2FoldChange	lfcSE	stat	pvalue	padj	
evm.TU.Scaffold_168_HRSCAF-201.174	75.91524744	3.788221741	0.604401558	6.267723321	3.66E-10	7.20E-06	neuronal acetylcholine receptor subunit alpha-10-like [Stylophora pistillata]
evm.TU.Scaffold_11_HRSCAF-12.9	218.3704191	2.626002804	0.493507073	5.321104698	1.03E-07	0.001013341	PREDICTED- uncharacterized protein LOC107344442 [Acropora digitifera]
evm.TU.Scaffold_11_HRSCAF-12.791	453.7115506	1.491543407	0.319263151	4.671830752	2.99E-06	0.009776756	uncharacterized protein LOC114973330 [Acropora millepora]
evm.TU.Scaffold_2593_HRSCAF-2754.948	18.82548135	5.269095626	1.123730516	4.688931689	2.75E-06	0.009776756	L-tyrosine/L-tryptophan isonitrile synthase family protein [Pseudomonas antarctica]
evm.TU.Scaffold_40164_HRSCAF-40545.655	153.1647499	2.199370825	0.466132232	4.718341008	2.38E-06	0.009776756	dual specificity protein phosphatase 1-A-like, partial [Orbicella faveolata]
evm.TU.Scaffold_40196_HRSCAF-40652.1137	795.4201773	2.059223688	0.437624033	4.705462986	2.53E-06	0.009776756	steroid 17-alpha-hydroxylase/17,20 lyase-like isoform X4 [Acropora millepora]
evm.TU.Scaffold_168_HRSCAF-201.49	101.3461996	2.106731648	0.462264142	4.557419573	5.18E-06	0.014537045	hypothetical protein SODALDRAFT_280356 [Sodiomyces alkalinus F11]
evm.TU.Scaffold_40135_HRSCAF-40458.746	468.6762831	2.048482278	0.453398943	4.518057018	6.24E-06	0.015329378	CD151 antigen [Exaiptasia pallida]
evm.TU.Scaffold_40161_HRSCAF-40537.1182	507.0760062	1.322783777	0.298190927	4.436029588	9.16E-06	0.020006595	PREDICTED- uncharacterized protein LOC107351686 [Acropora digitifera]
evm.TU.Scaffold_40161_HRSCAF-40537.261	51.74429477	2.151070035	0.489317682	4.396060294	1.10E-05	0.021660849	uncharacterized protein LOC110058701 [Orbicella faveolata]
evm.TU.Scaffold_117_HRSCAF-135.225	163.246779	1.511704092	0.348812032	4.333864521	1.47E-05	0.026172793	NA
evm.TU.Scaffold_40122_HRSCAF-40411.823	100.2199584	2.022296104	0.476275134	4.246066951	2.18E-05	0.029630207	PREDICTED- uncharacterized protein LOC107341959 [Acropora digitifera]
evm.TU.Scaffold_40135_HRSCAF-40458.55	152.0238205	2.046181585	0.47840279	4.277110475	1.89E-05	0.029630207	PREDICTED- uncharacterized protein LOC105313269 [Amphimedon queenslandica]
evm.TU.Scaffold_40196_HRSCAF-40652.1138	1759.118692	1.879632817	0.441334042	4.258979902	2.05E-05	0.029630207	steroid 17-alpha-hydroxylase/17,20 lyase-like isoform X6 [Acropora millepora]
evm.TU.Scaffold_40197_HRSCAF-40653.1452	111.4018809	2.047200303	0.483133505	4.237338714	2.26E-05	0.029630207	NA
evm.TU.Scaffold_40109_HRSCAF-40345.844	225.2938222	1.886779143	0.449239232	4.199942942	2.67E-05	0.03225302	PREDICTED- matrilysin-like [Hydra vulgaris]
evm.TU.Scaffold_40161_HRSCAF-40537.262	52.52283225	1.919725722	0.458175483	4.189935497	2.79E-05	0.03225302	uncharacterized protein LOC111345137 [Stylophora pistillata]
evm.TU.Scaffold_40197_HRSCAF-40653.1453	96.67539751	2.283152619	0.549852746	4.152298295	3.29E-05	0.034041332	NA
evm.TU.Scaffold_743_HRSCAF-861.676	122.9439473	1.80685813	0.435061673	4.153108033	3.28E-05	0.034041332	PREDICTED- uncharacterized protein LOC107341959 [Acropora digitifera]
evm.TU.Scaffold_40138_HRSCAF-40465.70	296.0859602	1.855370111	0.451146377	4.112567907	3.91E-05	0.037578491	predicted protein [Nematostella vectensis]
evm.TU.Scaffold_743_HRSCAF-861.1831	54.85309787	2.233438397	0.543871413	4.106555964	4.02E-05	0.037578491	PREDICTED- hemicentin-2-like [Hydra vulgaris]
evm.TU.Scaffold_11_HRSCAF-12.929	44.77036466	2.579099154	0.63355105	4.070862407	4.68E-05	0.038942457	PREDICTED- uncharacterized protein LOC107340932 [Acropora digitifera]
evm.TU.Scaffold_40141_HRSCAF-40491.833	148.9237094	1.947048035	0.478825648	4.066298546	4.78E-05	0.038942457	NA
evm.TU.Scaffold_40161_HRSCAF-40537.336	22709.50178	1.608314368	0.39555201	4.065999741	4.78E-05	0.038942457	PREDICTED- probable basic-leucine zipper transcription factor G [Hydra vulgaris]
evm.TU.Scaffold_743_HRSCAF-861.455	38.52307389	1.881686439	0.463725127	4.057762517	4.95E-05	0.038942457	NA
evm.TU.Scaffold_40114_HRSCAF-40359.703	503.4301742	1.810370288	0.448647923	4.035169219	5.46E-05	0.039121706	PREDICTED- TNF receptor-associated factor 5-like [Branchiostoma belcheri]
evm.TU.Scaffold_40135_HRSCAF-40458.251	16.90306063	3.433805858	0.854013302	4.020787321	5.80E-05	0.039121706	PREDICTED- uncharacterized protein LOC107351358 [Acropora digitifera]
evm.TU.Scaffold_40135_HRSCAF-40458.563	201.071056	1.758858499	0.439040548	4.006141357	6.17E-05	0.039121706	NA
evm.TU.Scaffold_40138_HRSCAF-40465.253	4562.176725	1.542661291	0.383281509	4.024877938	5.70E-05	0.039121706	PREDICTED- interferon regulatory factor 2-binding protein-like [Hydra vulgaris]
evm.TU.Scaffold_40141_HRSCAF-40491.78	748.9881713	1.672499035	0.413225045	4.04742901	5.18E-05	0.039121706	TNF receptor-associated factor 3-like [Stylophora pistillata]
evm.TU.Scaffold_40152_HRSCAF-40518.1298	4550.51591	1.184912275	0.295559051	4.009054269	6.10E-05	0.039121706	PREDICTED- matrilysin-like [Hydra vulgaris]
evm.TU.Scaffold_352_HRSCAF-431.1	38.68805925	2.369977848	0.60037354	3.947505492	7.90E-05	0.04433585	predicted protein [Nematostella vectensis]
evm.TU.Scaffold_40114_HRSCAF-40359.1484	132.4049159	1.512761902	0.382167609	3.958372891	7.55E-05	0.04433585	PREDICTED- sterol regulatory element-binding protein cleavage-activating protein [Corvus brachyrhynchos]
evm.TU.Scaffold_40170_HRSCAF-40571.161	21.97655218	2.750761536	0.69394816	3.963929435	7.37E-05	0.04433585	putative RNA-directed DNA polymerase from transposon BS [Stylophora pistillata]
evm.TU.Scaffold_40196_HRSCAF-40652.143	56.10095444	2.162026022	0.547280878	3.950487049	7.80E-05	0.04433585	NA
evm.TU.Scaffold_743_HRSCAF-861.492	402.2711334	1.488737729	0.380001007	3.917720482	8.94E-05	0.048792191	diacylglycerol kinase delta-like [Centruroides sculpturatus]
evm.TU.Scaffold_40164_HRSCAF-40545.1070	558.9202232	1.425363323	0.366355002	3.890661555	1.00E-04	0.05169568	uncharacterized protein LOC114527664 [Dendronephthya gigantea]
evm.TU.Scaffold_40196_HRSCAF-40652.14	19.58036338	-3.043394528	0.781830918	-3.89265052	9.92E-05	0.05169568	uncharacterized protein LOC114520952 [Dendronephthya gigantea]
evm.TU.Scaffold_40135_HRSCAF-40458.516	332.1343471	1.382068025	0.361154219	3.826808473	0.000129815	0.065407011	hypothetical protein pdam_00014866 [Pocillopora damicornis]
evm.TU.Scaffold_743_HRSCAF-861.1539	91.03784902	1.829274206	0.482466242	3.791507147	0.000149736	0.073557731	uncharacterized protein LOC110246070 [Exaiptasia pallida]
evm.TU.Scaffold_40122_HRSCAF-40411.1384	1608.512208	1.604477236	0.424586948	3.778913228	0.000157514	0.075491607	MAC/Perforin domain containing protein [Rhopilema esculentum]
evm.TU.Scaffold_743_HRSCAF-861.236	310.395469	1.676428464	0.444969464	3.767513506	0.000164882	0.077141045	NA
evm.TU.Scaffold_73_HRSCAF-87.667	1156.254868	1.47070094	0.392127098	3.750572061	0.000176432	0.080625134	galactoside 3(4)-L-fucosyltransferase-like [Acropora millepora]
evm.TU.Scaffold_40140_HRSCAF-40479.1113	272.7710951	1.545483191	0.412949675	3.742546085	0.000182165	0.081353283	NA
split_gene_evm.TU.Scaffold_40164_HRSCAF-4054	1191.514533	1.430777401	0.384145643	3.724570165	0.000195648	0.083966427	#N/A
evm.TU.Scaffold_40197_HRSCAF-40653.1527	44.27978595	2.17924949	0.585285805	3.723393718	0.000196563	0.083966427	NA
evm.TU.Scaffold_40135_HRSCAF-40458.58	80.36094535	1.594209862	0.428916425	3.716830996	0.000201737	0.084343358	uncharacterized protein LOC110253781, partial [Exaiptasia pallida]
evm.TU.Scaffold_40140_HRSCAF-40479.1109	124.5594146	1.417820736	0.383001409	3.701868202	0.000214018	0.086475133	NA
evm.TU.Scaffold_40170_HRSCAF-40571.69	398.4436268	1.44600851	0.390817868	3.699954959	0.000215638	0.086475133	predicted protein [Nematostella vectensis]
evm.TU.Scaffold_40141_HRSCAF-40491.199	447.4791474	1.563194103	0.424527743	3.682195401	0.000231234	0.090874988	NA
evm.TU.Scaffold_117_HRSCAF-135.145	159.6510058	1.829568938	0.500296584	3.656968682	0.000255216	0.092457291	PREDICTED- epithelial splicing regulatory protein 1-like [Hydra vulgaris]
evm.TU.Scaffold_11_HRSCAF-12.928	85.93613384	1.87943246	0.513163996	3.66244022	0.000249824	0.092457291	PREDICTED- uncharacterized protein LOC107341959 [Acropora digitifera]
evm.TU.Scaffold_40140_HRSCAF-40479.152	671.0221297	1.466188436	0.400279621	3.662910519	0.000249366	0.092457291	NA
evm.TU.Scaffold_40140_HRSCAF-40479.783	1390.097843	1.355894047	0.371131677	3.653404257	0.000258786	0.092457291	NA
evm.TU.Scaffold_73_HRSCAF-87.205	613.8130467	1.182308874	0.32230734	3.668265436	0.000244202	0.092457291	LOW QUALITY PROTEIN- zinc transporter 1-like [Stylophora pistillata]

evm.TU.Scaffold_2593_HRSCAF-2754.947	10.98717772	5.074476865	1.405740944	3.60982362	0.000306405	0.097674365	hypothetical protein SpCBS45565_g07402 [Spizellomyces sp. palustris]
evm.TU.Scaffold_40161_HRSCAF-40537.152	2904.258157	1.085035327	0.300261852	3.613630306	0.00030194	0.097674365	ERK3 [Aurelia aurita]
evm.TU.Scaffold_40161_HRSCAF-40537.174	3901.374956	1.438379694	0.39723596	3.620970507	0.0002935	0.097674365	NA
evm.TU.Scaffold_40161_HRSCAF-40537.928	409.0311108	1.622793429	0.447323913	3.627781527	0.000285867	0.097674365	uncharacterized protein LOC114796163 isoform X5 [Denticeps clupeoides]
evm.TU.Scaffold_40196_HRSCAF-40652.205	10.0444348	4.959586929	1.365257962	3.632710497	0.00028046	0.097674365	hypothetical protein NEMVEDRAFT_v1g146542 [Nematostella vectensis]
evm.TU.Scaffold_73_HRSCAF-87.571	2209.754243	1.28867747	0.357115577	3.608572552	0.000307886	0.097674365	matrix metalloproteinase-14-like precursor [Hydra vulgaris]
evm.TU.Scaffold_743_HRSCAF-861.1522	68.97485052	1.481237489	0.410505896	3.608322084	0.000308184	0.097674365	uncharacterized protein LOC114965363 [Acropora millepora]
evm.TU.Scaffold_73_HRSCAF-87.91	352.0659984	1.291984204	0.358708695	3.601764389	0.000316065	0.098582103	PREDICTED- uncharacterized protein LOC100206519 [Hydra vulgaris]

Table S2. KEGG enrichment of animals in the late day (ZT11) that had either experienced control conditions or had been exposed to LSD for two consecutive nights

Cluster	KEGG pathway ID	KEGG pathway name	# of DE genes in the pahtway	BgRatio	pvalue	p.adjusted	q-value (cut-off < 0.05)	Gene names	KEGG Ontology ID	
1	Up	map04657	IL-17 signaling pathway	45728	80/14171	3.67E-05	0.002321837	0.001376924	K03174/K09849/K06855	3
2	Up	map04668	TNF signaling pathway	45728	97/14171	6.54E-05	0.002321837	0.001376924	K03174/K09849/K09048	3
3	Up	map05203	Viral carcinogenesis	45728	174/14171	0.000368955	0.008731942	0.00517832	K03174/K09849/K09048	3
4	Up	map04927	Cortisol synthesis and secretion	45700	54/14171	0.000917945	0.016293525	0.00966258	K09048/K00512	2
5	Up	map05222	Small cell lung cancer	45700	74/14171	0.001716388	0.02437271	0.014453794	K03174/K09849	2
6	Up	map04928	Parathyroid hormone synthesis, secretion and action	45700	89/14171	0.002470981	0.025620358	0.015193689	K08003/K09048	2
7	Up	map04725	Cholinergic synapse	45700	90/14171	0.002525951	0.025620358	0.015193689	K04805/K09048	2
8	Up	map04064	NF-kappa B signaling pathway	45700	98/14171	0.002986456	0.026504794	0.015718187	K03174/K09849	2
9	Up	map04934	Cushing syndrome	45700	122/14171	0.004585658	0.034508431	0.0204646	K09048/K00512	2
10	Up	map05207	Chemical carcinogenesis - receptor activation	45700	129/14171	0.005112421	0.034508431	0.0204646	K04805/K09048	2
11	Up	map05161	Hepatitis B	45700	132/14171	0.005346377	0.034508431	0.0204646	K03174/K09048	2
12	Up	map04621	NOD-like receptor signaling pathway	45700	145/14171	0.006416383	0.036940777	0.021907058	K03174/K09849	2
13	Up	map05168	Herpes simplex virus 1 infection	45700	149/14171	0.006763804	0.036940777	0.021907058	K03174/K09849	2
14	Up	map05169	Epstein-Barr virus infection	45700	172/14171	0.008924254	0.045258717	0.026839862	K03174/K09849	2
15	Up	map05166	Human T-cell leukemia virus 1 infection	45700	191/14171	0.010913297	0.048914529	0.029007875	K09048/K01397	2
16	Up	map05163	Human cytomegalovirus infection	45700	192/14171	0.011022992	0.048914529	0.029007875	K09849/K09048	2
17	Up	map05165	Human papillomavirus infection	45700	231/14171	0.015679998	0.065487052	0.038835909	K03174/K09048	2

Table S3. Differentially expression genes of animals in the late day (ZT11) that had either experienced control conditions or had been exposed to MSD for two consecutive nights

Name	baseMean	log2FoldCha	lfcSE	stat	pvalue	padj	log2padj	annot	
evm.TU.Scaffold_40140_HRSCAF-40479.1106	301.3375344	-3.2560948	0.27047233	-12.038551	2.23E-33	4.64E-29	28.333482	NA	NA
evm.TU.Scaffold_743_HRSCAF-861.241	988.0433733	-2.933376	0.25829197	-11.356822	6.86E-30	7.14E-26	25.1463018	NA	NA
evm.TU.Scaffold_11_HRSCAF-12.1294	2424.923592	-4.676108	0.48673442	-9.6071036	7.46E-22	5.18E-18	17.2856702	uncharacterized protein LOC111334028 [Stylophora pistillata]	NA
evm.TU.Scaffold_40170_HRSCAF-40571.865	170.1187222	-6.3052239	0.74007605	-8.5196973	1.60E-17	6.66E-14	13.1765258	Rh-related protein [Branchiostoma floridae]	NA
evm.TU.Scaffold_743_HRSCAF-861.270	1877.161631	-2.9994974	0.38546859	-7.7814313	7.17E-15	2.49E-11	10.6038007	allorecognition 1 [Hydractinia symbiolongicarpus]	NA
evm.TU.Scaffold_40109_HRSCAF-40345.732	426.5535701	-2.3459418	0.30999926	-7.5675724	3.80E-14	1.13E-10	9.94692156	PREDICTED- E3 ubiquitin-protein ligase MIB1 isoform X3 [Crassostrea gigas]	K10645
evm.TU.Scaffold_40168_HRSCAF-40561.919	1874.896501	-2.7975611	0.38376144	-7.2898443	3.10E-13	7.18E-10	9.14387556	[Exaiptasia pallida]	NA
evm.TU.Scaffold_743_HRSCAF-861.1712	305.7897433	-4.0740532	0.57910942	-7.0350318	1.99E-12	4.15E-09	8.3819519	faveolata]	NA
evm.TU.Scaffold_743_HRSCAF-861.1705	462.8297048	-2.6400996	0.37796911	-6.9849612	2.85E-12	5.39E-09	8.26841124	putative leucine-rich repeat-containing protein [Stylophora pistillata]	NA
evm.TU.Scaffold_40141_HRSCAF-40491.824	243.0988209	-5.5051675	0.81614942	-6.7452936	1.53E-11	2.65E-08	7.57675413	NA	K04976
evm.TU.Scaffold_743_HRSCAF-861.1331	372.9205292	-2.367379	0.38050097	-6.2217424	4.92E-10	6.82E-07	6.16621563	uncharacterized protein LOC114525836 [Dendronephthya gigantea]	NA
evm.TU.Scaffold_11_HRSCAF-12.1278	369.849353	-3.5374864	0.57242512	-6.1798239	6.42E-10	8.35E-07	6.07831353	PREDICTED- apolipoprotein L3-like [Latimeria chalumnae]	NA
evm.TU.Scaffold_40197_HRSCAF-40653.1383	721.4741735	-2.9782113	0.48293814	-6.1668588	6.97E-10	8.53E-07	6.06905097	NA	NA
evm.TU.Scaffold_40140_HRSCAF-40479.866	6732.790761	-2.319845	0.3806391	-6.0946051	1.10E-09	1.27E-06	5.89619628	NA	K18668
evm.TU.Scaffold_40141_HRSCAF-40491.270	3074.835158	-5.2570475	0.86894084	-6.0499487	1.45E-09	1.44E-06	5.84163751	myosin essential light chain [Aurelia sp. 2017-HT]	NA
evm.TU.Scaffold_168_HRSCAF-201.1719	638.5540093	-2.2911045	0.37839176	-6.054848	1.41E-09	1.44E-06	5.84163751	Eph5, partial [Hydra vulgaris]	K05110
evm.TU.Scaffold_40161_HRSCAF-40537.288	6460.16154	-1.5422565	0.25954823	-5.9420804	2.81E-09	2.55E-06	5.59345982	uncharacterized protein LOC110042142 isoform X1 [Orbicella faveolata]	NA
evm.TU.Scaffold_40170_HRSCAF-40571.911	1391.551685	-3.2002502	0.53933272	-5.9337215	2.96E-09	2.57E-06	5.59006688	PREDICTED- inhibin beta A chain [Dinoponera quadriceps]	K22687
evm.TU.Scaffold_743_HRSCAF-861.544	646.3797089	-2.3708926	0.40187301	-5.8996064	3.64E-09	3.03E-06	5.51855737	ETS translocation variant 4-like [Stylophora pistillata]	NA
evm.TU.Scaffold_11_HRSCAF-12.247	113.6063625	-5.0458479	0.85826935	-5.879096	4.13E-09	3.30E-06	5.48148606	microadriaticum]	NA
evm.TU.Scaffold_2593_HRSCAF-2754.859	187.4433307	-3.8510236	0.65998966	-5.8349756	5.38E-09	3.96E-06	5.40230481	PREDICTED- E3 ubiquitin-protein ligase RNF213-like [Acropora digitifera]	NA
evm.TU.Scaffold_40196_HRSCAF-40652.367	1345.087164	-3.7558514	0.64478256	-5.8249891	5.71E-09	3.96E-06	5.40230481	hypothetical protein TrispH2_009981 [Trichoplax sp. H2]	NA
evm.TU.Scaffold_40140_HRSCAF-40479.700	2056.609847	-1.1743425	0.20266344	-5.7945451	6.85E-09	4.60E-06	5.33724217	NA	K09444
evm.TU.Scaffold_743_HRSCAF-861.1353	38.99832589	-5.9766751	1.04358014	-5.7270878	1.02E-08	6.65E-06	5.17717836	PREDICTED- filamin-A-like [Hydra vulgaris]	K04437
evm.TU.Scaffold_40140_HRSCAF-40479.1108	225.8814776	-2.5081303	0.44243933	-5.6688683	1.44E-08	9.07E-06	5.04239271	NA	NA
evm.TU.Scaffold_11_HRSCAF-12.1268	441.0004335	-5.589904	0.98761039	-5.6600295	1.51E-08	9.27E-06	5.03292027	ETS translocation variant 3-like protein [Columba livia]	K09432
evm.TU.Scaffold_168_HRSCAF-201.734	1927.932275	-4.1794451	0.74248121	-5.6290247	1.81E-08	1.05E-05	4.9788107	unnamed protein product, partial [Tetraodon nigroviridis]	K11861
evm.TU.Scaffold_73_HRSCAF-87.658	120.097617	-2.1699049	0.38517038	-5.6336234	1.76E-08	1.05E-05	4.9788107	PREDICTED- galactoside 3(4)-L-fucosyltransferase-like [Acropora digitifera]	K00753
evm.TU.Scaffold_168_HRSCAF-201.1132	148.0578218	-3.7632408	0.66937806	-5.621996	1.89E-08	1.06E-05	4.97469414	uncharacterized protein LOC114544031 [Dendronephthya gigantea]	NA
evm.TU.Scaffold_40196_HRSCAF-40652.330	16956.99153	-1.9342153	0.34577578	-5.5938425	2.22E-08	1.20E-05	4.92081875	PREDICTED- uncharacterized protein LOC105847273 [Hydra vulgaris]	K12800
evm.TU.Scaffold_168_HRSCAF-201.1720	416.7502373	-2.5778769	0.46284718	-5.5696069	2.55E-08	1.30E-05	4.88605665	NA	NA
evm.TU.Scaffold_11_HRSCAF-12.480	1293.375706	-1.8000774	0.32293815	-5.5740624	2.49E-08	1.30E-05	4.88605665	DNA damage-regulated autophagy modulator protein 2 [Exaiptasia pallida]	K21956
evm.TU.Scaffold_743_HRSCAF-861.1716	119.1865257	-3.1269969	0.56268329	-5.5572947	2.74E-08	1.36E-05	4.86646109	pistillata]	NA
evm.TU.Scaffold_40135_HRSCAF-40458.629	4423.089258	-1.501061	0.27090003	-5.5410144	3.01E-08	1.46E-05	4.83564714	protein c-ets-2-A-like isoform X2 [Orbicella faveolata]	K09442
evm.TU.Scaffold_40164_HRSCAF-40545.759	1053.50909	-5.2811691	0.95816673	-5.5117433	3.55E-08	1.68E-05	4.77469072	mesoglein, partial [Aurelia sp. 1 LSA-2014]	NA
evm.TU.Scaffold_11_HRSCAF-12.804	193.2410379	-4.9439997	0.89886909	-5.5002444	3.79E-08	1.74E-05	4.75945075	gigantea]	K15261
evm.TU.Scaffold_40109_HRSCAF-40345.155	43.07268658	-9.1145612	1.66618912	-5.4703041	4.49E-08	1.99E-05	4.70114692	Nuclear migration protein nudC [Stylophora pistillata]	NA
evm.TU.Scaffold_40170_HRSCAF-40571.195	522.3569929	-2.8912854	0.52952394	-5.4601599	4.76E-08	2.06E-05	4.68613278	Ras-like protein 2 [Eufriesea mexicana]	K07838
evm.TU.Scaffold_40140_HRSCAF-40479.1104	98.74030043	-5.366447	0.98975002	-5.4220227	5.89E-08	2.36E-05	4.627088	NA	NA
evm.TU.Scaffold_73_HRSCAF-87.45	1341.852181	-1.0978627	0.20227059	-5.427693	5.71E-08	2.36E-05	4.627088	actin-like protein 7A [Acropora millepora]	NA
evm.TU.Scaffold_743_HRSCAF-861.1590	1959.12315	-2.2408223	0.41393165	-5.413508	6.18E-08	2.43E-05	4.61439373	digitifera]	NA
evm.TU.Scaffold_40170_HRSCAF-40571.756	1982.148023	-3.2410094	0.6004751	-5.3974086	6.76E-08	2.61E-05	4.58335949	NA	NA
evm.TU.Scaffold_40170_HRSCAF-40571.704	4665.684204	-1.5494882	0.28807565	-5.3787546	7.50E-08	2.84E-05	4.54668166	receptor-type tyrosine-protein phosphatase S isoform X1 [Exaiptasia pallida]	K05695
evm.TU.Scaffold_40196_HRSCAF-40652.368	1212.503223	-3.4147881	0.63884221	-5.3452762	9.03E-08	3.36E-05	4.47366072	uncharacterized protein LOC113684617 [Pocillopora damicornis]	NA
evm.TU.Scaffold_40135_HRSCAF-40458.166	188.0207164	-2.9498809	0.55304551	-5.3338845	9.61E-08	3.51E-05	4.45469288	NA	NA
evm.TU.Scaffold_40164_HRSCAF-40545.381	421.8921448	-7.6480399	1.43589291	-5.3263303	1.00E-07	3.54E-05	4.45099674	predicted protein [Nematostella vectensis]	NA
evm.TU.Scaffold_40140_HRSCAF-40479.1111	282.0439327	-4.4935998	0.84610734	-5.3109098	1.09E-07	3.78E-05	4.4225082	NA	NA
evm.TU.Scaffold_40170_HRSCAF-40571.110	493.3268656	-2.018596	0.38077861	-5.3012325	1.15E-07	3.86E-05	4.4134127	lymphocyte expansion molecule-like [Orbicella faveolata]	NA
evm.TU.Scaffold_11_HRSCAF-12.1285	666.9692798	-3.0448728	0.57692474	-5.2777643	1.31E-07	4.19E-05	4.37778598	uncharacterized protein LOC114520100 [Dendronephthya gigantea]	NA
evm.TU.Scaffold_40140_HRSCAF-40479.47	103.2725755	-2.6634964	0.50464194	-5.2779926	1.31E-07	4.19E-05	4.37778598	NA	K15734
evm.TU.Scaffold_40196_HRSCAF-40652.331	63.54062166	-7.6710735	1.45684928	-5.2655231	1.40E-07	4.39E-05	4.35753548	hypothetical protein EV586_101397 [Tumebacillus sp. BK434]	NA
evm.TU.Scaffold_11_HRSCAF-12.218	3017.210166	-1.5345135	0.2915355	-5.2635562	1.41E-07	4.39E-05	4.35753548	uncharacterized protein LOC113672169 [Pocillopora damicornis]	NA
evm.TU.Scaffold_40197_HRSCAF-40653.1385	4228.944137	-2.4120554	0.45914844	-5.2533239	1.49E-07	4.45E-05	4.35163999	NA	NA
evm.TU.Scaffold_40197_HRSCAF-40653.1115_evm.TU.Scaffold_40197_HRSCAF-40653.1118	626.7748157	-3.0055658	0.5749615	-5.2274209	1.72E-07	5.04E-05	4.29756946	putative leucine-rich repeat-containing protein DDB_G0281931 isoform X1 [Exaiptasia pallida]	NA
evm.TU.Scaffold_40122_HRSCAF-40411.1155	592.5992474	-3.0881208	0.59315087	-5.2062991	1.93E-07	5.57E-05	4.25414481	PREDICTED- heme-binding protein 2-like [Branchiostoma belcheri]	NA
evm.TU.Scaffold_40196_HRSCAF-40652.204	37.81937371	-5.1562676	0.99327711	-5.1911672	2.09E-07	5.88E-05	4.23062267	PREDICTED- protein PRQFV-amide-like, partial [Biomphalaria glabrata]	NA
evm.TU.Scaffold_40135_HRSCAF-40458.357	534.8061849	-3.4285709	0.66251132	-5.1751129	2.28E-07	6.24E-05	4.20481541	NA	NA
evm.TU.Scaffold_168_HRSCAF-201.1095	1063.544062	-1.4728628	0.28796532	-5.1147229	3.14E-07	8.32E-05	4.07987667	PREDICTED- uncharacterized protein LOC101235770 [Hydra vulgaris]	K15259
evm.TU.Scaffold_189_HRSCAF-231.1	22.44236313	-5.8279832	1.14147904	-5.1056419	3.30E-07	8.58E-05	4.06651271	ring 31 finger protein, partial [Mytilus galloprovincialis]	K11974
evm.TU.Scaffold_40197_HRSCAF-40653.1305	216.7237329	-3.2666356	0.64013199	-5.1030657	3.34E-07	8.59E-05	4.06600684	tripartite motif-containing protein 67-like [Stylophora pistillata]	NA
evm.TU.Scaffold_40152_HRSCAF-40518.125	4575.381339	-3.2966279	0.65312414	-5.0474752	4.48E-07	0.00010985	3.95919203	NA	NA
evm.TU.Scaffold_40161_HRSCAF-40537.17	3085.145308	-1.3299681	0.2637278	-5.0429575	4.58E-07	0.00010985	3.95919203	protein 1-like [Hydra vulgaris]	K07369
evm.TU.Scaffold_40161_HRSCAF-40537.285	475.999759	-3.4198678	0.67886788	-5.0376044	4.71E-07	0.00011154	3.95257715	NA	NA
evm.TU.Scaffold_40138_HRSCAF-40465.607	27.3284494	-5.194174	1.032157	-5.0323488	4.85E-07	0.00011209	3.95042151	NA	NA
evm.TU.Scaffold_40140_HRSCAF-40479.1112_evm.TU.Scaffold_40140_HRSCAF-40479.1114	110.2662714	-3.5591599	0.70713343	-5.0332226	4.82E-07	0.00011209	3.95042151	NA	NA
evm.TU.Scaffold_743_HRSCAF-861.551	336.8089549	-1.308022	0.26091502	-5.013211	5.35E-07	0.0001207	3.91831072	gigantea]	NA
evm.TU.Scaffold_40196_HRSCAF-40652.328	1564.238309	-2.4204844	0.4843372	-4.9975191	5.81E-07	0.00012864	3.8906341	uncharacterized protein LOC113665785 [Pocillopora damicornis]	NA
evm.TU.Scaffold_40161_HRSCAF-40537.206	34.96567909	-7.4865578	1.50743189	-4.9664319	6.82E-07	0.00014791	3.82999072	PREDICTED- protein PRRC1-B-like [Hydra vulgaris]	NA
evm.TU.Scaffold_40114_HRSCAF-40359.1240	409.2798902	-4.1308734	0.83356154	-4.955691	7.21E-07	0.00015471	3.81047319	NA	NA
evm.TU.Scaffold_743_HRSCAF-861.238	103.3644169	-2.5297715	0.511346	-4.9472794	7.53E-07	0.0001599	3.79615154	NA	NA
evm.TU.Scaffold_40152_HRSCAF-40518.1370	206.16549	-1.8279963	0.37058344	-4.9327523	8.11E-07	0.00016882	3.77256839	CMT1A duplicated region transcript 1 protein-like [Orbicella faveolata]	K10266
evm.TU.Scaffold_40140_HRSCAF-40479.895	1315.75292	-1.5577769	0.31801399	-4.8984542	9.66E-07	0.00019718	3.70513053	NA	NA
evm.TU.Scaffold_40140_HRSCAF-40479.185	283.4031083	-4.3619801	0.89632021	-4.8665421	1.14E-06	0.00022738	3.64325538	NA	NA
evm.TU.Scaffold_40138_HRSCAF-40465.437	288.4203764	-2.0042878	0.4126018	-4.8576805	1.19E-06	0.0002333	3.63208154	NA	NA
evm.TU.Scaffold_40114_HRSCAF-40359.502	5462.663763	-1.1229595	0.23273304	-4.825097	1.40E-06	0.00026732	3.57297667	X3 [Hydra vulgaris]	K06233
evm.TU.Scaffold_743_HRSCAF-861.361	149.0020217	-5.491329	1.13866623	-4.8225975	1.42E-06	0.00026823	3.57149751	DLG [Oscarella lobularis]	K12076
evm.TU.Scaffold_40140_HRSCAF-40479.1113	513.7663012	-3.3238008	0.69227577	-4.8012669	1.58E-06	0.00029576	3.52906497	NA	NA
evm.TU.Scaffold_168_HRSCAF-201.395	642.9229616	-1.504601	0.314781	-4.7798343	1.75E-06	0.00032616	3.48656797	ras-related protein Rab-20-like [Lingula anatina]	K07911
evm.TU.Scaffold_40122_HRSCAF-40411.1227	530.8112087	-1.6395707	0.34326906	-4.7763428	1.79E-06	0.00032894	3.48288859	[Acanthaster planci]	NA
evm.TU.Scaffold_40114_HRSCAF-40359.728	168.9510974	-2.6989185	0.56565177	-4.7713428	1.83E-06	0.00033135	3.47971958	stabilizer of axonemal microtubules 1-like [Stylophora pistillata]	NA
evm.TU.Scaffold_40122_HRSCAF-40411.922	258.675488	-2.3312952	0.48902248	-4.7672557	1.87E-06	0.00033365	3.47671278	Ribonuclease Oy [Trichoplax sp. H2]	NA
evm.TU.Scaffold_40161_HRSCAF-40537.233	370.6254648	-1.5312014	0.32124405	-4.766474	1.87E-06	0.00033365	3.47671278	NA	NA
evm.TU.Scaffold_11_HRSCAF-12.564	39.60728673	-3.9611063	0.83338563	-4.7530293	2.00E-06	0.00035361	3.45148037	uncharacterized protein LOC114525836 [Dendronephthya gigantea]	NA
evm.TU.Scaffold_40141_HRSCAF-40491.272	81.42520922	-2.4643667	0.51996229	-4.7395104	2.14E-06	0.00037486	3.42613322	NA	NA
evm.TU.Scaffold_40138_HRSCAF-40465.1903	1187.754992	-2.597784	0.54932143	-4.7290782	2.26E-06	0.00039135	3.40743244	coiled-coil domain-containing protein 170-like [Pocillopora damicornis]	NA
evm.TU.Scaffold_40138_HRSCAF-40465.1830	138.7513963	-2.9125725	0.61749944	-4.7167208	2.40E-06	0.00041244	3.38463817	digitifera]	NA
evm.TU.Scaffold_40161_HRSCAF-40537.596	239.8666291	-2.109521	0.450411	-4.6835468	2.82E-06	0.00046967	3.32821088	NA	NA
evm.TU.Scaffold_40140_HRSCAF-40479.407	2527.892735	-1.881965	0.40312943	-4.6683889	3.04E-06	0.00050166	3.29958793	NA	NA
evm.TU.Scaffold_40197_HRSCAF-40653.767	807.9614502	-2.5779222	0.55450795	-4.6490266	3.34E-06	0.00053417	3.27231807	NA	NA

evm.TU.Scaffold_40152_HRSCAF-40518.235	1877.772852	-2.3284541	0.50079189	-4.6495443	3.33E-06	0.00053417	3.27231807	Contactin-associated protein-like 5 [Stylophora pistillata]	NA
evm.TU.Scaffold_11_HRSCAF-12.282	192.0524664	-10.069949	2.17936854	-4.6205813	3.83E-06	0.00060824	3.21592859	sp. G]	K09705
evm.TU.Scaffold_40122_HRSCAF-40411.571	903.4428689	-2.500788	0.54229425	-4.6114965	4.00E-06	0.00063062	3.20023019	NA	NA
evm.TU.Scaffold_40161_HRSCAF-40537.850	150.1309676	-3.3614957	0.73302468	-4.5857879	4.52E-06	0.0006874	3.162793	kowalevskii]	NA
evm.TU.Scaffold_11_HRSCAF-12.928	69.64554363	-6.0410454	1.31899576	-4.580034	4.65E-06	0.00069641	3.15713251	PREDICTED- uncharacterized protein LOC107341959 [Acropora digitifera]	NA
evm.TU.Scaffold_40140_HRSCAF-40479.1152	25880.52648	-3.124101	0.68204248	-4.5805079	4.64E-06	0.00069641	3.15713251	NA	NA
evm.TU.Scaffold_168_HRSCAF-201.1716	732.5049436	-1.8326026	0.40073904	-4.5730573	4.81E-06	0.00071488	3.14576868	megakaryocyte-associated tyrosine-protein kinase [Myripristis murdjan]	NA
evm.TU.Scaffold_40109_HRSCAF-40345.1049	765.0375483	-1.4981228	0.32812597	-4.5656942	4.98E-06	0.00073519	3.13360336	PREDICTED- uncharacterized protein LOC105844767 [Hydra vulgaris]	NA
evm.TU.Scaffold_40122_HRSCAF-40411.65	135.5611977	-2.5274377	0.55541444	-4.5505437	5.35E-06	0.0007844	3.10546242	Nephrocystin-3 [Exaiptasia pallida]	NA
evm.TU.Scaffold_743_HRSCAF-861.302	649.9007667	-1.9750353	0.43415759	-4.5491208	5.39E-06	0.0007844	3.10546242	PREDICTED- hemicentin-2-like, partial [Hydra vulgaris]	K17341
evm.TU.Scaffold_40168_HRSCAF-40561.983	20.18558863	-4.6395682	1.02260373	-4.5370147	5.71E-06	0.00082502	3.08353763	[Hydra vulgaris]	K21956
evm.TU.Scaffold_40168_HRSCAF-40561.861	195.4751401	-9.6607633	2.13162334	-4.5321156	5.84E-06	0.00083857	3.07646327	yessoensis]	NA
evm.TU.Scaffold_2593_HRSCAF-2754.529	150.8640842	-5.1025812	1.12859582	-4.5211768	6.15E-06	0.00087705	3.05697812	tctex1 domain-containing protein 1-B-like [Orbicella faveolata]	NA
evm.TU.Scaffold_40152_HRSCAF-40518.233	1931.227346	-2.2200985	0.49217001	-4.5108366	6.46E-06	0.00091465	3.03874744	uncharacterized protein LOC110049839 [Orbicella faveolata]	NA
evm.TU.Scaffold_11_HRSCAF-12.1437	963.5991884	-1.2123004	0.26959715	-4.4967107	6.90E-06	0.00097094	3.01280984	putative MAP7 domain-containing protein 3 [Scophthalmus maximus]	NA
evm.TU.Scaffold_40168_HRSCAF-40561.28	3095.061916	-1.5629595	0.34854804	-4.4842012	7.32E-06	0.00102276	2.99022669	NA	NA
evm.TU.Scaffold_40114_HRSCAF-40359.232	2024.166353	-2.6516242	0.59350404	-4.4677441	7.90E-06	0.0010973	2.95967343	kinesin-like protein KIF28P [Acropora millepora]	NA
evm.TU.Scaffold_40197_HRSCAF-40653.1384	877.7804293	-2.2048159	0.49387847	-4.4642884	8.03E-06	0.00110778	2.95554805	NA	NA
evm.TU.Scaffold_40135_HRSCAF-40458.171	1476.834477	-2.8209105	0.63401164	-4.449304	8.61E-06	0.00118013	2.92807089	NA	NA
evm.TU.Scaffold_40197_HRSCAF-40653.1123	590.4622	-2.1667825	0.48732938	-4.4462383	8.74E-06	0.00118926	2.92472209	[Exaiptasia pallida]	NA
evm.TU.Scaffold_11_HRSCAF-12.44	233.0503778	-3.8617886	0.87179892	-4.4296782	9.44E-06	0.00126778	2.89695782	Phthiocerol synthesis polyketide synthase type I PpsC [Stylophora pistillata]	NA
evm.TU.Scaffold_40152_HRSCAF-40518.234	3784.855665	-2.2345734	0.50468615	-4.4276496	9.53E-06	0.00127155	2.89566656	millepora]	NA
evm.TU.Scaffold_40135_HRSCAF-40458.147	1848.078339	-2.8083775	0.63509584	-4.4219744	9.78E-06	0.00129711	2.88702487	hypothetical protein CAPTEDRAFT_203197 [Capitella teleta]	K00515
evm.TU.Scaffold_117_HRSCAF-135.1342	140.0978656	-3.2651491	0.73863885	-4.4204947	9.85E-06	0.00129775	2.88680762	uncharacterized protein LOC114540544 [Dendronephthya gigantea]	NA
evm.TU.Scaffold_40170_HRSCAF-40571.804	1009.988699	-2.1269578	0.482974	-4.4038763	1.06E-05	0.0013925	2.85620417	uncharacterized protein LOC111342858 isoform X1 [Stylophora pistillata]	K03899
evm.TU.Scaffold_743_HRSCAF-861.1717	205.3197101	-2.6334297	0.59884562	-4.3975101	1.09E-05	0.00141776	2.84839789	putative leucine-rich repeat-containing protein [Stylophora pistillata]	NA
evm.TU.Scaffold_40109_HRSCAF-40345.210	955.2907065	-1.6486188	0.37511455	-4.3949743	1.11E-05	0.00142394	2.84650831	predicted protein [Nematostella vectensis]	K04377
evm.TU.Scaffold_40152_HRSCAF-40518.1318	521.9725833	-2.7327277	0.6221356	-4.3924953	1.12E-05	0.00143144	2.84422594	trichohyalin-like [Acropora millepora]	NA
evm.TU.Scaffold_743_HRSCAF-861.1074	13343.40947	-1.2829047	0.29239341	-4.3875979	1.15E-05	0.00144772	2.83931453	hypothetical protein pdam_00022233 [Pocillopora damicornis]	K17341
evm.TU.Scaffold_2593_HRSCAF-2754.479	150.8469032	-3.7136123	0.84745857	-4.3820576	1.18E-05	0.00146582	2.83392054	uncharacterized protein LOC110051211 [Orbicella faveolata]	NA
evm.TU.Scaffold_40196_HRSCAF-40652.313	26.19558895	-7.865854	1.79924059	-4.3717633	1.23E-05	0.00152753	2.81601025	Nephrocystin-3 [Exaiptasia pallida]	NA
evm.TU.Scaffold_40168_HRSCAF-40561.1015	2469.36945	-1.1760345	0.27172857	-4.3279751	1.50E-05	0.00182176	2.7395086	hypothetical protein TRIADDRAFT_51134 [Trichoplax adhaerens]	K04456
evm.TU.Scaffold_11_HRSCAF-12.251	551.3673392	-1.7243213	0.39965334	-4.3145424	1.60E-05	0.00189248	2.7229687	borealin-like [Pocillopora damicornis]	NA
evm.TU.Scaffold_40168_HRSCAF-40561.982	298.6215059	-1.2607999	0.29216918	-4.3153075	1.59E-05	0.00189248	2.7229687	[Hydra vulgaris]	K21956
evm.TU.Scaffold_40114_HRSCAF-40359.1634	905.5844931	-1.7864401	0.41573233	-4.2970921	1.73E-05	0.00202434	2.69371762	PREDICTED- uncharacterized protein LOC105844767 [Hydra vulgaris]	NA
evm.TU.Scaffold_743_HRSCAF-861.871	1504.470324	-1.6722064	0.38944351	-4.2938358	1.76E-05	0.00203145	2.69219493	faveolata]	K17341
evm.TU.Scaffold_743_HRSCAF-861.272	220.8175748	-2.3865954	0.55858056	-4.2726073	1.93E-05	0.0021794	2.66166345	NA	NA
evm.TU.Scaffold_40135_HRSCAF-40458.673	2902.773588	-1.6000555	0.37453557	-4.2721056	1.94E-05	0.0021794	2.66166345	NA	NA
evm.TU.Scaffold_40152_HRSCAF-40518.456	2049.75076	-1.5985501	0.37495475	-4.2633148	2.01E-05	0.00225478	2.64689563	Mucin-like protein, partial [Trichoplax sp. H2]	NA
evm.TU.Scaffold_40161_HRSCAF-40537.340	1139.682643	-1.8294097	0.43212297	-4.2335395	2.30E-05	0.00253435	2.59613272	NA	NA
evm.TU.Scaffold_40141_HRSCAF-40491.286	2605.110339	-1.1179955	0.26464612	-4.2244924	2.39E-05	0.00262445	2.58096203	B-cell scaffold protein with ankyrin repeats-like [Dendronephthya gigantea]	K12230
evm.TU.Scaffold_40114_HRSCAF-40359.233	2376.413438	-2.8071819	0.6661772	-4.2138666	2.51E-05	0.00272243	2.5650436	PREDICTED- kinesin-like protein KIF28P [Acropora digitifera]	NA
evm.TU.Scaffold_168_HRSCAF-201.1633	47.90043034	-4.7183219	1.12146156	-4.207297	2.58E-05	0.00278824	2.55466954	Conodipine-M alpha chain [Stylophora pistillata]	NA
evm.TU.Scaffold_40161_HRSCAF-40537.341	870.8671665	-1.9145375	0.45648049	-4.1941278	2.74E-05	0.00292494	2.53388289	NA	NA
evm.TU.Scaffold_40164_HRSCAF-40545.86	105.5978651	-3.3829729	0.80923828	-4.180441	2.91E-05	0.00307515	2.51213398	protein SSUH2 homolog isoform X1 [Ciona intestinalis]	NA
evm.TU.Scaffold_40135_HRSCAF-40458.98	73.0482362	-5.5186847	1.32071235	-4.1785668	2.93E-05	0.00308494	2.51075384	uncharacterized protein LOC114946912 [Acropora millepora]	NA
evm.TU.Scaffold_11_HRSCAF-12.46	109.9894533	-6.7912243	1.63316169	-4.1583294	3.21E-05	0.00332099	2.47873243	uncharacterized protein LOC114542747 [Dendronephthya gigantea]	K13612
evm.TU.Scaffold_168_HRSCAF-201.351	527.8666233	-2.7792317	0.66986292	-4.1489559	3.34E-05	0.0033908	2.46969795	fibrillin-2-like isoform X1 [Penaeus vannamei]	NA
evm.TU.Scaffold_2593_HRSCAF-2754.285	1641.483564	-1.8416255	0.44383118	-4.1493828	3.33E-05	0.0033908	2.46969795	PREDICTED- uncharacterized protein LOC107327625 [Acropora digitifera]	K05463
evm.TU.Scaffold_40170_HRSCAF-40571.238	5329.211294	-1.4156565	0.34120778	-4.1489573	3.34E-05	0.0033908	2.46969795	PREDICTED- kinesin-1 heavy chain-like [Hydra vulgaris]	K10392
evm.TU.Scaffold_40196_HRSCAF-40652.312	28.10335763	-6.885353	1.66788312	-4.1281988	3.66E-05	0.00358758	2.44519865	NA	NA
evm.TU.Scaffold_40196_HRSCAF-40652.1147	28.56948425	-6.7828873	1.64222044	-4.1303147	3.62E-05	0.00358758	2.44519865	retinol dehydrogenase 8-like [Dendronephthya gigantea]	NA
evm.TU.Scaffold_40114_HRSCAF-40359.452	398.765507	-2.975185	0.72018093	-4.1311633	3.61E-05	0.00358758	2.44519865	titin-like isoform X4 [Stylophora pistillata]	K05695
evm.TU.Scaffold_11_HRSCAF-12.1178	480.6152156	-1.9871067	0.48125249	-4.1290316	3.64E-05	0.00358758	2.44519865	[Pocillopora damicornis]	K05048
evm.TU.Scaffold_73_HRSCAF-87.687	1784.545922	-1.3666064	0.33122153	-4.1259589	3.69E-05	0.00358758	2.44519865	NA	NA
evm.TU.Scaffold_40122_HRSCAF-40411.577	3446.09155	-3.1252425	0.75951293	-4.1147983	3.88E-05	0.00370133	2.43164208	NA	NA
evm.TU.Scaffold_40168_HRSCAF-40561.934	205.6554891	-1.4851609	0.36085302	-4.1156949	3.86E-05	0.00370133	2.43164208	acetylglucosaminyltransferase A-like, partial [Hydra vulgaris]	K00738
evm.TU.Scaffold_168_HRSCAF-201.1518	350.2215181	-1.3689253	0.33345497	-4.1052779	4.04E-05	0.00380477	2.41967216	uncharacterized protein LOC114948699 [Acropora millepora]	K12026
evm.TU.Scaffold_11_HRSCAF-12.965	19.16990632	-3.9234478	0.95648987	-4.101923	4.10E-05	0.00384298	2.4153321	NA	NA
evm.TU.Scaffold_40152_HRSCAF-40518.161	54.54510127	-2.3001191	0.56500525	-4.0709695	4.68E-05	0.00435197	2.36131381	predicted protein [Nematostella vectensis]	K14708
evm.TU.Scaffold_40135_HRSCAF-40458.222	2449.97543	-2.8004384	0.68844178	-4.0677927	4.75E-05	0.00435341	2.36117033	hypothetical protein pdam_00017818 [Pocillopora damicornis]	K14616
evm.TU.Scaffold_743_HRSCAF-861.1748	460.198117	-1.7567086	0.4316745	-4.0695214	4.71E-05	0.00435341	2.36117033	predicted protein [Nematostella vectensis]	NA
evm.TU.Scaffold_168_HRSCAF-201.1723	21.51710756	-5.8991875	1.45557175	-4.0528318	5.06E-05	0.00462115	2.33525003	hypothetical protein DSY43_03210 [Gammaproteobacteria bacterium]	NA
evm.TU.Scaffold_40138_HRSCAF-40465.1818	58.97680108	-3.9077558	0.96650925	-4.0431645	5.27E-05	0.00479493	2.31921737	[Orbicella faveolata]	K08844
evm.TU.Scaffold_40152_HRSCAF-40518.236	1018.095845	-2.2773335	0.5654497	-4.0274732	5.64E-05	0.00508197	2.29396833	belcheri]	NA
evm.TU.Scaffold_40196_HRSCAF-40652.1194	1039.444797	-1.6085188	0.40183188	-4.0029647	6.26E-05	0.0055901	2.25258081	PREDICTED- tetratricopeptide repeat protein 28-like [Aplysia californica]	NA
evm.TU.Scaffold_743_HRSCAF-861.1666	14.73220464	-4.1711742	1.04275249	-4.0001575	6.33E-05	0.00563265	2.24928739	protein C-ets-2-like isoform X2 [Acropora millepora]	K09442
evm.TU.Scaffold_40164_HRSCAF-40545.712	142.8447696	-1.5981442	0.39987907	-3.9965686	6.43E-05	0.00569435	2.24455561	putative leucine-rich repeat-containing protein [Stylophora pistillata]	NA
novel_gene_432_5d572bfb	8.548711523	-6.4567763	1.61683144	-3.9934752	6.51E-05	0.00574475	2.24072924	NA	#N/A
evm.TU.Scaffold_168_HRSCAF-201.1440	17570.30507	-2.0168292	0.50543117	-3.9903142	6.60E-05	0.00579636	2.23684442	NA	NA
evm.TU.Scaffold_40170_HRSCAF-40571.864	729.6090046	-1.9335392	0.48479574	-3.9883585	6.65E-05	0.00579636	2.23684442	ammonium transporter Rh type B-like [Dendronephthya gigantea]	NA
evm.TU.Scaffold_117_HRSCAF-135.352	2251.892184	-1.0383935	0.26030822	-3.9890925	6.63E-05	0.00579636	2.23684442	NA	NA
evm.TU.Scaffold_168_HRSCAF-201.1335	325.9953206	-3.4696104	0.87020231	-3.9871308	6.69E-05	0.00580215	2.23641097	uncharacterized protein LOC110067018 [Orbicella faveolata]	NA
evm.TU.Scaffold_743_HRSCAF-861.351	807.5096251	-1.9383592	0.48636977	-3.9853612	6.74E-05	0.00582131	2.23497927	predicted protein [Nematostella vectensis]	NA
evm.TU.Scaffold_11_HRSCAF-12.55	684.6087986	-1.1885491	0.29888345	-3.9766306	6.99E-05	0.00596487	2.2243988	predicted protein [Nematostella vectensis]	NA
evm.TU.Scaffold_73_HRSCAF-87.38	433.5055808	-1.8953179	0.47721483	-3.9716241	7.14E-05	0.00606679	2.2170409	uncharacterized protein C3orf84-like [Acropora millepora]	NA
evm.TU.Scaffold_40122_HRSCAF-40411.824	88.1167823	-3.2514358	0.82222281	-3.9544462	7.67E-05	0.0064668	2.18931044	uncharacterized protein LOC110246883 [Exaiptasia pallida]	NA
evm.TU.Scaffold_168_HRSCAF-201.1683	710.8441053	-1.3543274	0.34243572	-3.954983	7.65E-05	0.0064668	2.18931044	BTB/POZ domain-containing protein 6-B-like [Dendronephthya gigantea]	K10478
evm.TU.Scaffold_40168_HRSCAF-40561.955	178.9296538	-4.2820635	1.08369278	-3.951363	7.77E-05	0.00652429	2.18546708	Transposon Ty3-G Gag-Pol polyprotein [Exaiptasia pallida]	NA
evm.TU.Scaffold_73_HRSCAF-87.780	192.5033694	-1.8925203	0.48043127	-3.9392114	8.17E-05	0.00675475	2.17039091	NA	NA
evm.TU.Scaffold_40161_HRSCAF-40537.286	2062.464415	-1.2999088	0.33043263	-3.93396	8.36E-05	0.00684976	2.16432484	predicted protein [Nematostella vectensis]	NA
evm.TU.Scaffold_40197_HRSCAF-40653.1304	107.1882364	-3.6745032	0.93507465	-3.9296362	8.51E-05	0.00694675	2.15821839	tripartite motif-containing protein 67-like [Stylophora pistillata]	K12026
split_gene_evm.TU.Scaffold_40170_HRSCAF-40571.704-g694	44.62299761	-2.3984891	0.61128553	-3.9236805	8.72E-05	0.00706541	2.15086251	NA	#N/A
evm.TU.Scaffold_40140_HRSCAF-40479.196	949.4894318	-2.5349676	0.6490276	-3.9057933	9.39E-05	0.00752129	2.12370772	NA	NA
evm.TU.Scaffold_117_HRSCAF-135.188	53.74455509	-3.9660764	1.01607597	-3.9033266	9.49E-05	0.00753817	2.12273396	biogenic amine-like GPCR [Tripedalia cystophora]	K04285
evm.TU.Scaffold_40140_HRSCAF-40479.1146	2410.429716	-1.5646277	0.40093205	-3.902476	9.52E-05	0.00753817	2.12273396	NA	K23029
evm.TU.Scaffold_168_HRSCAF-201.1977	1784.855265	-1.9666998	0.50429143	-3.899927	9.62E-05	0.00758912	2.11980847	predicted protein [Nematostella vectensis]	NA
evm.TU.Scaffold_743_HRSCAF-861.783	685.7291567	-2.118505	0.54422333	-3.8927126	9.91E-05	0.00778897	2.10852025	protein phosphatase 1 regulatory subunit 3G [Acanthochromis polyacanthus]	NA
evm.TU.Scaffold_40164_HRSCAF-40545.810	463.1277747	-0.9538638	0.24569227	-3.8823517	0.00010345	0.00809797	2.09162405	PREDICTED- probable inactive purple acid phosphatase 2 [Hydra vulgaris]	K22390
evm.TU.Scaffold_11_HRSCAF-12.88	3046.359351	-0.8787198	0.22675366	-3.8752177	0.00010653	0.0083077	2.0805192	phospholipid-transporting ATPase IA-like [Stylophora pistillata]	K14802
evm.TU.Scaffold_40164_HRSCAF-40545.604	1293.744775	-1.8214999	0.47026553	-3.8733434	0.00010735	0.00834064	2.07880068	PREDICTED- protein FAM154A-like [Hydra vulgaris]	NA
evm.TU.Scaffold_40196_HRSCAF-40652.638	10.60866202	-7.1304419	1.84311045	-3.8687003	0.00010942	0.00843808	2.07375631	gigantea]	NA
evm.TU.Scaffold_40122_HRSCAF-40411.1130	1645.986735	-0.8817019	0.22788861	-3.869004	0.00010928	0.00843808	2.07375631	PREDICTED- protein strawberry notch homolog 1-like [Hydra vulgaris]	NA

evm.TU.Scaffold_11_HRSCAF-12.802	31.02775519	-6.0680684	1.56892469	-3.8676607	0.00010988	0.00844285	2.07351082	hypothetical protein AC249_AIPGENE11200 [Exaiptasia pallida]	NA
evm.TU.Scaffold_40152_HRSCAF-40518.1317	1415.226361	-2.6872138	0.70052266	-3.8360126	0.00012505	0.00953754	2.02056354	pistillata]	NA
evm.TU.Scaffold_40161_HRSCAF-40537.697	808.9783343	-1.5757821	0.41092205	-3.8347471	0.00012569	0.0095518	2.01991501	stabilizer of axonemal microtubules 2 [Exaiptasia pallida]	NA
evm.TU.Scaffold_168_HRSCAF-201.1294	385.8723852	-3.6656036	0.9564401	-3.8325491	0.00012682	0.00960252	2.01761469	uncharacterized protein LOC110232517 [Exaiptasia pallida]	NA
evm.TU.Scaffold_40122_HRSCAF-40411.1151	1800.217124	-1.3414648	0.35062458	-3.8259291	0.00013028	0.0098221	2.00779587	predicted protein [Nematostella vectensis]	K18628
evm.TU.Scaffold_117_HRSCAF-135.1347	12.86033286	-3.9902913	1.04425697	-3.8211776	0.00013282	0.00991217	2.00383143	hypothetical protein BRAFLDRAFT_79985 [Branchiostoma floridae]	NA
evm.TU.Scaffold_2593_HRSCAF-2754.240	1448.546669	-1.6586048	0.43459995	-3.8163944	0.00013542	0.01007012	1.99696557	NA	NA
evm.TU.Scaffold_40188_HRSCAF-40634.2	8.512681439	-6.1879346	1.62342554	-3.8116529	0.00013804	0.01022007	1.99054617	uncharacterized protein LOC114355981 [Ostrinia furnacalis]	NA
evm.TU.Scaffold_168_HRSCAF-201.1348	977.0690087	-1.7360321	0.45553369	-3.810985	0.00013841	0.01022007	1.99054617	lens membrane protein mp19-1 [Danio rerio]	NA
evm.TU.Scaffold_40141_HRSCAF-40491.823	20.7586454	-7.3665604	1.9357206	-3.8055907	0.00014147	0.01037186	1.9841434	NA	NA
evm.TU.Scaffold_40152_HRSCAF-40518.228	938.5031201	-1.2627449	0.3326634	-3.7958635	0.00014713	0.01071171	1.97014107	predicted protein [Nematostella vectensis]	K17988
evm.TU.Scaffold_11_HRSCAF-12.735	765.0952176	-0.7969033	0.21018442	-3.7914481	0.00014977	0.010866	1.96393038	PREDICTED- round spermatid basic protein 1-like [Hydra vulgaris]	K22610
evm.TU.Scaffold_73_HRSCAF-87.299	352.4673191	-1.7858464	0.47172339	-3.7857916	0.00015322	0.01103927	1.95705984	X2 [Hydra vulgaris]	K12800
evm.TU.Scaffold_11_HRSCAF-12.1306	339.3717832	-7.0542875	1.87060797	-3.7711202	0.00016252	0.01143215	1.94187224	uncharacterized protein LOC111334028 [Stylophora pistillata]	NA
evm.TU.Scaffold_40140_HRSCAF-40479.1107	37.46757354	-3.7426941	0.99238634	-3.7714084	0.00016233	0.01143215	1.94187224	NA	NA
evm.TU.Scaffold_2593_HRSCAF-2754.866	72.89884514	-3.4312568	0.90950889	-3.7726479	0.00016152	0.01143215	1.94187224	E3 ubiquitin-protein ligase rnf213-alpha-like [Acropora millepora]	NA
evm.TU.Scaffold_40135_HRSCAF-40458.756	1086.662954	-1.7611799	0.46823452	-3.7613199	0.00016902	0.01182639	1.92714773	uncharacterized protein LOC113684686 [Pocillopora damicornis]	NA
evm.TU.Scaffold_40196_HRSCAF-40652.267	34.3139742	-4.6749856	1.24452071	-3.7564546	0.00017234	0.01196137	1.92221904	[Branchiostoma belcheri]	NA
evm.TU.Scaffold_40168_HRSCAF-40561.851	160.182882	-2.3553859	0.62825591	-3.7490867	0.00017748	0.01227735	1.91089522	PREDICTED- neuropeptide Y receptor type 4-like [Monodelphis domestica]	NA
evm.TU.Scaffold_40168_HRSCAF-40561.1013	193.8890992	-2.6442217	0.70650732	-3.7426672	0.00018208	0.01255369	1.90122864	PREDICTED- gremlin-1 [Poecilia reticulata]	K23318
evm.TU.Scaffold_743_HRSCAF-861.1197	9.470845952	-5.1530144	1.37718938	-3.7416891	0.00018279	0.01256107	1.9009735	protein phosphatase 1 regulatory subunit 3G-like [Salmo trutta]	NA
evm.TU.Scaffold_40122_HRSCAF-40411.1132	478.9475208	-1.4964834	0.40039172	-3.7375483	0.00018582	0.01272768	1.89525079	PREDICTED- protein strawberry notch homolog 1-like [Hydra vulgaris]	NA
evm.TU.Scaffold_11_HRSCAF-12.504	300.3516402	-1.2583709	0.33753578	-3.7281112	0.00019292	0.01308464	1.88323812	uncharacterized protein LOC111339188 [Stylophora pistillata]	NA
evm.TU.Scaffold_743_HRSCAF-861.1826	578.6583664	-0.981638	0.26328433	-3.7284329	0.00019267	0.01308464	1.88323812	uncharacterized protein CXorf65 homolog [Orbicella faveolata]	NA
evm.TU.Scaffold_40161_HRSCAF-40537.213	4765.450951	-1.4363293	0.38584963	-3.7225106	0.00019725	0.01329182	1.87641542	cartilage oligomeric matrix protein-like [Pocillopora damicornis]	K04659
evm.TU.Scaffold_40114_HRSCAF-40359.389	2035.788866	-1.6346755	0.43947139	-3.7196403	0.00019951	0.01335733	1.87428044	uncharacterized protein LOC111326449 [Stylophora pistillata]	NA
evm.TU.Scaffold_40170_HRSCAF-40571.853	732.7096587	-1.5936955	0.42910189	-3.7140259	0.00020399	0.01361358	1.86602781	outer dynein arm protein 1-like [Orbicella faveolata]	K23732
evm.TU.Scaffold_40135_HRSCAF-40458.191	869.2640115	-1.9280176	0.51975002	-3.7095094	0.00020766	0.01377045	1.8610518	Procollagen C-endopeptidase enhancer 1 [Stylophora pistillata]	NA
evm.TU.Scaffold_40140_HRSCAF-40479.1101	270.7122591	-2.6734396	0.72109643	-3.7074648	0.00020935	0.01383801	1.85892645	NA	NA
evm.TU.Scaffold_40164_HRSCAF-40545.644	3861.087726	-0.9441223	0.25477781	-3.7056692	0.00021083	0.01389232	1.85722532	hypothetical protein pdam_00020377 [Pocillopora damicornis]	K20640
evm.TU.Scaffold_40135_HRSCAF-40458.646	91.83038525	-3.3484481	0.90434801	-3.7026101	0.00021339	0.01396025	1.85510696	NA	NA
evm.TU.Scaffold_40170_HRSCAF-40571.935	2384.534957	-1.5384784	0.41557622	-3.7020368	0.00021388	0.01396025	1.85510696	PREDICTED- uncharacterized protein LOC105843939 [Hydra vulgaris]	K17102
evm.TU.Scaffold_40122_HRSCAF-40411.1486	467.4991863	-0.9662105	0.26118262	-3.6993676	0.00021614	0.01406379	1.85189775	NA	NA
evm.TU.Scaffold_40196_HRSCAF-40652.1129	32.79548235	-2.7709523	0.75006225	-3.6942964	0.0002205	0.01425832	1.84593176	uncharacterized protein LOC114525283 [Dendronephthya gigantea]	NA
evm.TU.Scaffold_11_HRSCAF-12.604	1349.145852	-1.5582608	0.42220408	-3.6907762	0.00022357	0.01441236	1.84126502	endothelin-converting enzyme homolog [Ctenocephalides felis]	NA
evm.TU.Scaffold_40114_HRSCAF-40359.402	588.5976819	-2.0573256	0.55758712	-3.6896935	0.00022452	0.01442916	1.84075898	Protein white [Stylophora pistillata]	NA
evm.TU.Scaffold_40196_HRSCAF-40652.639	13.5726441	-5.6584963	1.53445652	-3.6876224	0.00022636	0.01450231	1.83856279	pistillata]	NA
evm.TU.Scaffold_40140_HRSCAF-40479.1128	21.18060825	-6.9046376	1.87431829	-3.6838128	0.00022977	0.01463087	1.83473	NA	NA
evm.TU.Scaffold_40168_HRSCAF-40561.244	612.9813891	-2.2107925	0.60044646	-3.6819145	0.00023149	0.01469532	1.83282092	putative N-acetylated-alpha-linked acidic dipeptidase [Stylophora pistillata]	K14592
evm.TU.Scaffold_11_HRSCAF-12.1642	1062.975501	-1.5199954	0.41365775	-3.6745243	0.00023829	0.01503556	1.82288031	kinesin-like protein KIF9 [Stylophora pistillata]	K10397
evm.TU.Scaffold_40114_HRSCAF-40359.515	361.7948907	-1.8851775	0.51569732	-3.6555891	0.00025659	0.01594854	1.79727918	WD repeat-containing protein 49-like isoform X2 [Acropora millepora]	NA
evm.TU.Scaffold_40152_HRSCAF-40518.991	1676.66937	-1.9412604	0.5313678	-3.6533271	0.00025886	0.01604187	1.79474501	NA	NA
evm.TU.Scaffold_40114_HRSCAF-40359.1623	322.8392116	-1.1085552	0.30368887	-3.650299	0.00026194	0.01618402	1.79091359	NA	NA
evm.TU.Scaffold_40196_HRSCAF-40652.1153	156.6301378	-4.0730814	1.11675834	-3.6472362	0.00026508	0.01631234	1.78748363	retinol dehydrogenase 8-like [Pocillopora damicornis]	K11150
evm.TU.Scaffold_40114_HRSCAF-40359.874	1817.392251	-1.1345105	0.31113493	-3.6463618	0.00026598	0.01631234	1.78748363	vulgaris]	K15105
evm.TU.Scaffold_73_HRSCAF-87.49	2728.331939	-1.7513201	0.48106695	-3.6404914	0.00027212	0.01656738	1.78074611	NA	NA
evm.TU.Scaffold_40197_HRSCAF-40653.809	239.205022	-2.9052118	0.79891176	-3.6364614	0.00027641	0.01677956	1.77521951	NA	NA
evm.TU.Scaffold_743_HRSCAF-861.1751	4235.607839	-1.1632104	0.32040331	-3.6304568	0.00028292	0.01706247	1.76795813	vulgaris]	K06734
evm.TU.Scaffold_117_HRSCAF-135.517	8391.843792	-0.9913841	0.27311585	-3.629903	0.00028353	0.01706247	1.76795813	uncharacterized protein LOC114522730 [Dendronephthya gigantea]	NA
evm.TU.Scaffold_40114_HRSCAF-40359.654	147.4832276	-1.4098903	0.38910377	-3.6234301	0.00029072	0.01740638	1.75929159	PREDICTED- uncharacterized protein LOC105844605 [Hydra vulgaris]	NA
evm.TU.Scaffold_168_HRSCAF-201.1445	5420.6999	-3.3321593	0.92172291	-3.6151422	0.00030018	0.01790948	1.7469171	type IV secretion protein Rhs [Vibrio mangrovi]	NA
evm.TU.Scaffold_11_HRSCAF-12.323	107.1031072	-2.9874244	0.82661463	-3.6140474	0.00030145	0.01793393	1.74632465	gracilis]	K00994
evm.TU.Scaffold_11_HRSCAF-12.1281	61.31671328	-2.2896286	0.63418837	-3.6103289	0.00030581	0.01814118	1.7413344	uncharacterized protein LOC114515865 [Dendronephthya gigantea]	NA
evm.TU.Scaffold_40122_HRSCAF-40411.161	688.7251358	-1.3983855	0.38783315	-3.6056369	0.00031139	0.01832686	1.73691187	[Exaiptasia pallida]	NA
evm.TU.Scaffold_40161_HRSCAF-40537.712	673.615	-0.8679081	0.24078566	-3.604484	0.00031277	0.01832686	1.73691187	[Acropora digitifera]	NA
evm.TU.Scaffold_40138_HRSCAF-40465.1929	1751.338201	-0.6852397	0.19011587	-3.6043266	0.00031296	0.01832686	1.73691187	damicornis]	NA
evm.TU.Scaffold_40135_HRSCAF-40458.146	794.3647817	-2.4129619	0.67139092	-3.5939746	0.00032567	0.01886137	1.7244267	hypothetical protein CAPTEDRAFT_203197 [Capitella teleta]	K10252
evm.TU.Scaffold_168_HRSCAF-201.1231	1545.697265	-0.6089683	0.16945769	-3.5936307	0.0003261	0.01886137	1.7244267	Tyrosine-protein phosphatase non-receptor type 11 [Araneus ventricosus]	K07293
evm.TU.Scaffold_40141_HRSCAF-40491.42	49.9103392	-1.8474578	0.51549597	-3.5838453	0.00033857	0.01947447	1.71053431	NA	NA
evm.TU.Scaffold_40197_HRSCAF-40653.1113	184.2532297	-2.937035	0.8199353	-3.5820326	0.00034093	0.01955611	1.70871746	[Stylophora pistillata]	NA
evm.TU.Scaffold_2593_HRSCAF-2754.481	233.290383	-1.9254365	0.53765148	-3.5811981	0.00034202	0.0195648	1.70852461	PREDICTED- protocadherin Fat 2-like isoform X2 [Acropora digitifera]	K16506
evm.TU.Scaffold_40097_HRSCAF-40281.1	49.35875316	-2.3291984	0.65164104	-3.574358	0.00035109	0.02001088	1.69873375	NA	NA
evm.TU.Scaffold_40152_HRSCAF-40518.232	6629.925026	-1.8133801	0.50739946	-3.5738708	0.00035174	0.02001088	1.69873375	uncharacterized protein LOC113680433 [Pocillopora damicornis]	NA
evm.TU.Scaffold_743_HRSCAF-861.389	1170.536738	-1.2723312	0.35631411	-3.5708134	0.00035587	0.02019078	1.69484684	metabotropic glutamate receptor 3-like [Orbicella faveolata]	K04606
evm.TU.Scaffold_40196_HRSCAF-40652.1154	659.5449705	-2.9038714	0.81338906	-3.5700891	0.00035686	0.02019167	1.69482776	predicted protein [Nematostella vectensis]	NA
evm.TU.Scaffold_40164_HRSCAF-40545.760	197.5957263	-3.7536302	1.05282924	-3.5652792	0.00036347	0.02045933	1.68910864	NA	NA
evm.TU.Scaffold_40197_HRSCAF-40653.1127	685.9687856	-3.1715855	0.8899237	-3.5638848	0.00036541	0.02050806	1.68807534	[Exaiptasia pallida]	NA
evm.TU.Scaffold_168_HRSCAF-201.240	1270.581361	-1.1343376	0.31877049	-3.5584776	0.00037301	0.02082259	1.68146519	uncharacterized protein LOC113686703 [Pocillopora damicornis]	NA
evm.TU.Scaffold_40135_HRSCAF-40458.518	3090.747331	-1.4799242	0.41670992	-3.5514495	0.00038312	0.02117256	1.67422669	CD151 antigen [Exaiptasia pallida]	K06537
evm.TU.Scaffold_168_HRSCAF-201.450	3726.695988	-1.0439704	0.29391969	-3.55189	0.00038248	0.02117256	1.67422669	macrophage-expressed gene 1 protein-like [Acropora millepora]	NA
evm.TU.Scaffold_40141_HRSCAF-40491.288	126.4032917	-1.1381395	0.32129454	-3.5423557	0.00039657	0.02150361	1.66748854	damicornis]	K04811
evm.TU.Scaffold_40135_HRSCAF-40458.173	25.90860276	-3.0615139	0.86486517	-3.5398741	0.00040032	0.02165044	1.66453325	PREDICTED- uncharacterized protein LOC105847456 [Hydra vulgaris]	NA
evm.TU.Scaffold_40152_HRSCAF-40518.172	1817.879663	-1.4892425	0.42098495	-3.5375196	0.0004039	0.02178781	1.6617865	ubiquitin carboxyl-terminal hydrolase 31-like [Orbicella faveolata]	K11852
evm.TU.Scaffold_117_HRSCAF-135.1028	35.88550903	-5.5949014	1.58617637	-3.5272883	0.00041984	0.022244	1.65278704	PREDICTED- uncharacterized protein LOC107171508 [Diuraphis noxia]	NA
evm.TU.Scaffold_40197_HRSCAF-40653.467	9.770826601	-4.8368869	1.37011123	-3.5302878	0.00041511	0.022244	1.65278704	NA	NA
evm.TU.Scaffold_246_HRSCAF-308.2	21.26680367	-3.0586431	0.86658336	-3.5295428	0.00041628	0.022244	1.65278704	craniofacial development protein 2-like [Diaphorina citri]	NA
evm.TU.Scaffold_40138_HRSCAF-40465.507	506.6084062	-2.015932	0.57151231	-3.5273641	0.00041972	0.022244	1.65278704	uncharacterized protein LOC114532461 [Dendronephthya gigantea]	NA
evm.TU.Scaffold_743_HRSCAF-861.1901	816.8310824	-1.0609126	0.30071471	-3.5279705	0.00041876	0.022244	1.65278704	E3 ubiquitin-protein ligase RNF13 [Lingula anatina]	K15706
evm.TU.Scaffold_40114_HRSCAF-40359.404	14.01053439	-3.3387163	0.94795196	-3.5220311	0.00042825	0.02241043	1.64954975	NA	NA
evm.TU.Scaffold_40152_HRSCAF-40518.53	2102.030473	-1.0566872	0.29989947	-3.5234713	0.00042593	0.02241043	1.64954975	PREDICTED- ras-like protein rasS [Hydra vulgaris]	K04353
evm.TU.Scaffold_743_HRSCAF-861.1579	1282.963699	-0.7980949	0.22664787	-3.5212989	0.00042944	0.02241043	1.64954975	PREDICTED- uncharacterized protein LOC106804913 [Priapulus caudatus]	NA
evm.TU.Scaffold_40196_HRSCAF-40652.1037	6.241486458	-5.9482115	1.69045732	-3.5186996	0.00043367	0.02251827	1.64746507	hypothetical protein C0J52_17340 [Blattella germanica]	NA
evm.TU.Scaffold_2593_HRSCAF-2754.1422	72.94609744	-2.2539483	0.64049877	-3.5190517	0.00043309	0.02251827	1.64746507	like [Hydra vulgaris]	NA
evm.TU.Scaffold_117_HRSCAF-135.80	344.9049732	-1.2703926	0.36121282	-3.5170198	0.00043642	0.02254661	1.64691871	like [Dendronephthya gigantea]	NA
evm.TU.Scaffold_40141_HRSCAF-40491.637	1279.299432	-1.1296079	0.32121588	-3.5166626	0.00043701	0.02254661	1.64691871	NA	K02580
evm.TU.Scaffold_11_HRSCAF-12.929	77.30038596	-3.7136356	1.05690197	-3.5136992	0.00044191	0.02271978	1.6435958	PREDICTED- uncharacterized protein LOC107340932 [Acropora digitifera]	NA
evm.TU.Scaffold_40122_HRSCAF-40411.245	47.3733514	-2.4330488	0.69288443	-3.5114785	0.00044562	0.02285402	1.64103747	LIM domain kinase 1 [Daphnia magna]	K05743
evm.TU.Scaffold_40197_HRSCAF-40653.806	7.40892908	-6.0343697	1.72083574	-3.5066506	0.00045379	0.02299406	1.63838426	caudatus]	NA
evm.TU.Scaffold_2593_HRSCAF-2754.289_evm.TU.Scaffold_2593_HRSCAF-2754.291	24.25856613	-3.5348869	1.00787627	-3.5072627	0.00045274	0.02299406	1.63838426	uncharacterized protein LOC110062702 [Orbicella faveolata]	K17253
evm.TU.Scaffold_40122_HRSCAF-40411.912	138.5290597	-2.9284199	0.83467749	-3.5084448	0.00045074	0.02299406	1.63838426	[Aplysia californica]	K16518
evm.TU.Scaffold_40109_HRSCAF-40345.1048	4449.198495	-1.6818131	0.47935834	-3.5084675	0.0004507	0.02299406	1.63838426	CHAT domain-containing protein [Geminocystis sp. NIES-3708]	NA
evm.TU.Scaffold_40135_HRSCAF-40458.770	665.8955894	-1.6278725	0.4651615	-3.4995856	0.00046598	0.02337994	1.63115663	lamin-L(I)-like [Pocillopora damicornis]	K12641

evm.TU.Scaffold_40170_HRSCAF-40571.799	2230.16598	-1.5184514	0.43383737	-3.5000476	0.00046518	0.02337994	1.63115663	Neuropilin-2 [Stylophora pistillata]	K18032
evm.TU.Scaffold_40197_HRSCAF-40653.1490	4009.710668	-1.2289121	0.35109956	-3.5001814	0.00046494	0.02337994	1.63115663	pistillata]	NA
evm.TU.Scaffold_40114_HRSCAF-40359.1393	448.8198409	-1.5840882	0.45313308	-3.4958565	0.00047254	0.0235469	1.62806626	PREDICTED- vesicular inhibitory amino acid transporter-like [Hydra vulgaris]	K15015
evm.TU.Scaffold_40138_HRSCAF-40465.411	350.2617106	-1.0671744	0.30527621	-3.4957665	0.0004727	0.0235469	1.62806626	NA	NA
evm.TU.Scaffold_168_HRSCAF-201.356	509.9802648	-1.9606503	0.56174571	-3.4902809	0.00048251	0.02397826	1.62018241	uncharacterized protein LOC114949688 [Acropora millepora]	K04905
evm.TU.Scaffold_40138_HRSCAF-40465.543	139.7424837	-2.6927707	0.77280169	-3.4844265	0.00049319	0.02433846	1.61370683	Apolipoprotein L3 [Stylophora pistillata]	NA
evm.TU.Scaffold_40114_HRSCAF-40359.862	1702.63862	-1.0817175	0.31044711	-3.4843856	0.00049327	0.02433846	1.61370683	enkurin-like [Stylophora pistillata]	NA
evm.TU.Scaffold_40164_HRSCAF-40545.49	1753.269304	-0.7843482	0.22517976	-3.4832091	0.00049544	0.02438789	1.61282574	PREDICTED- uncharacterized protein LOC100209854 [Hydra vulgaris]	NA
evm.TU.Scaffold_40140_HRSCAF-40479.1109	294.6321595	-3.2990306	0.94768539	-3.4811453	0.00049928	0.02441767	1.61229574	NA	K08123
evm.TU.Scaffold_11_HRSCAF-12.1561	38.10656885	-2.8900209	0.83037942	-3.4803618	0.00050074	0.02441767	1.61229574	Protein brambleberry [Exaiptasia pallida]	NA
evm.TU.Scaffold_40140_HRSCAF-40479.180	209.4397488	-2.4392647	0.70077633	-3.4808034	0.00049991	0.02441767	1.61229574	NA	K13912
evm.TU.Scaffold_11_HRSCAF-12.960	27.37875059	-4.2653549	1.22661426	-3.4773401	0.00050642	0.02452381	1.61041206	uncharacterized protein LOC114972132 [Acropora millepora]	NA
evm.TU.Scaffold_11_HRSCAF-12.1560	33.09994426	-3.0426011	0.87585265	-3.4738732	0.000513	0.02478366	1.60583465	predicted protein [Nematostella vectensis]	NA
evm.TU.Scaffold_40141_HRSCAF-40491.643	74.18047179	-4.2008526	1.20983482	-3.472253	0.00051611	0.02481856	1.60522347	NA	NA
evm.TU.Scaffold_40197_HRSCAF-40653.1119	267.4129435	-3.3196524	0.95598378	-3.4724986	0.00051564	0.02481856	1.60522347	[Exaiptasia pallida]	NA
evm.TU.Scaffold_40109_HRSCAF-40345.992	1628.291012	-0.7751351	0.22338777	-3.4699084	0.00052064	0.02492111	1.60343267	E3 ubiquitin-protein ligase MIB1-like isoform X2 [Octopus vulgaris]	K10645
evm.TU.Scaffold_117_HRSCAF-135.1595	1023.472657	-1.437845	0.41500705	-3.4646279	0.00053097	0.02529924	1.59689259	WD repeat-containing protein 97-like isoform X4 [Acropora millepora]	NA
evm.TU.Scaffold_40122_HRSCAF-40411.122	1404.649027	-1.0314874	0.29834574	-3.4573558	0.0005455	0.02581475	1.58813216	ERK5, partial [Aurelia aurita]	K04464
evm.TU.Scaffold_40196_HRSCAF-40652.299	55.37362144	-8.2536255	2.39105977	-3.4518692	0.00055672	0.0261112	1.5831732	NA	NA
evm.TU.Scaffold_40140_HRSCAF-40479.1055	410.5499512	-2.8857313	0.83591542	-3.452181	0.00055608	0.0261112	1.5831732	NA	K06760
evm.TU.Scaffold_40141_HRSCAF-40491.776	1117.070032	-0.8491823	0.2460793	-3.4508482	0.00055883	0.02614812	1.58255956	PREDICTED- protein SAND-like [Hydra vulgaris]	K20195
evm.TU.Scaffold_40161_HRSCAF-40537.715	2448.078117	-2.8133233	0.81552889	-3.4496917	0.00056123	0.0262015	1.58167383	faveolata]	NA
evm.TU.Scaffold_40122_HRSCAF-40411.420	428.8595524	-1.463154	0.42553341	-3.4383999	0.00058516	0.02713644	1.56644713	PREDICTED- ras-related protein Rab-36-like, partial [Hydra vulgaris]	K07921
evm.TU.Scaffold_743_HRSCAF-861.1941	481.0388389	-1.1187282	0.32532628	-3.4387883	0.00058432	0.02713644	1.56644713	hypothetical protein SAV14893_014500 [Streptomyces avermitilis]	NA
evm.TU.Scaffold_40114_HRSCAF-40359.1243	287.2880877	-2.960815	0.8612683	-3.4377383	0.00058659	0.02713797	1.56642261	NA	NA
evm.TU.Scaffold_40168_HRSCAF-40561.876	510.6102684	-0.8586252	0.24980504	-3.437181	0.0005878	0.02713797	1.56642261	PREDICTED- toll-like receptor 2 [Acropora digitifera]	K05398
evm.TU.Scaffold_11_HRSCAF-12.1236	116.3018884	-2.2222523	0.64692618	-3.435094	0.00059235	0.02728734	1.56403885	vulgaris]	NA
evm.TU.Scaffold_40170_HRSCAF-40571.1106	18.67345816	-6.937194	2.02006292	-3.4341475	0.00059442	0.02732236	1.56348187	NA	NA
evm.TU.Scaffold_40141_HRSCAF-40491.455	4.465345132	-5.5736732	1.62412825	-3.4317938	0.0005996	0.0273951	1.56232714	NA	NA
evm.TU.Scaffold_40122_HRSCAF-40411.347	1937.882507	-0.9049922	0.26367731	-3.4321961	0.00059871	0.0273951	1.56232714	PREDICTED- vascular endothelial growth factor C-like [Hydra vulgaris]	NA
evm.TU.Scaffold_40197_HRSCAF-40653.1392	1094.366228	-1.0957477	0.3194265	-3.4303594	0.00060278	0.02746419	1.56123325	kowalevskii]	K13421
evm.TU.Scaffold_40152_HRSCAF-40518.1319	1483.728078	-2.0290783	0.5916634	-3.429447	0.00060481	0.02749652	1.56072227	trichohyalin-like [Acropora millepora]	NA
evm.TU.Scaffold_40114_HRSCAF-40359.388	878.1660916	-2.1964846	0.64072873	-3.4281037	0.00060781	0.02751279	1.5604653	PREDICTED- glycine betaine transporter OpuD-like [Acropora digitifera]	K02168
evm.TU.Scaffold_40152_HRSCAF-40518.992	101.6724919	-2.0712657	0.60548924	-3.4208134	0.00062434	0.02819966	1.54975621	NA	NA
evm.TU.Scaffold_40170_HRSCAF-40571.934	1002.533321	-1.2224919	0.35782818	-3.4164216	0.0006345	0.02853465	1.54462745	vulgaris]	K18598
evm.TU.Scaffold_2593_HRSCAF-2754.967	297.0510353	-1.7103998	0.50088049	-3.4147863	0.00063832	0.02856602	1.54415033	xenotropic and polytropic retrovirus receptor [Cyanea capillata]	NA
evm.TU.Scaffold_40141_HRSCAF-40491.104	324.8453623	-1.716393	0.50289944	-3.4129945	0.00064253	0.02864841	1.54289942	NA	NA
evm.TU.Scaffold_40196_HRSCAF-40652.198	1544.375973	-1.6731905	0.49032945	-3.4123802	0.00064398	0.02865171	1.5428495	like [Hydra vulgaris]	K06704
evm.TU.Scaffold_168_HRSCAF-201.29	1311.501768	-1.0293184	0.30176321	-3.4110136	0.00064722	0.0287343	1.54159939	uncharacterized protein LOC114955139 [Acropora millepora]	NA
evm.TU.Scaffold_117_HRSCAF-135.452	6.136436771	-5.6546976	1.65847611	-3.4095743	0.00065064	0.02882489	1.54023236	uncharacterized protein LOC113665248 [Pocillopora damicornis]	NA
evm.TU.Scaffold_73_HRSCAF-87.676	266.2788586	-1.9078553	0.56003647	-3.4066626	0.00065762	0.02907227	1.536521	PREDICTED- galactoside 3(4)-L-fucosyltransferase-like [Acropora digitifera]	K00753
evm.TU.Scaffold_11_HRSCAF-12.1445	268.4369003	-1.6938922	0.49783835	-3.4024945	0.00066774	0.02945682	1.5308141	uncharacterized protein LOC110983960 isoform X3 [Acanthaster planci]	NA
evm.TU.Scaffold_40141_HRSCAF-40491.284	1008.028203	-1.4483255	0.42600968	-3.3997478	0.00067448	0.02950427	1.53011516	PREDICTED- tetraspanin-5-like [Hydra vulgaris]	K17346
evm.TU.Scaffold_40109_HRSCAF-40345.1206	3235.145154	-1.3179652	0.38758815	-3.4004269	0.00067281	0.02950427	1.53011516	tektin-2-like [Pocillopora damicornis]	K18629
evm.TU.Scaffold_40109_HRSCAF-40345.781	3204.458433	-0.8731008	0.25677198	-3.4002962	0.00067313	0.02950427	1.53011516	tyrosine-protein kinase Src42A [Sipha flava]	K08892
evm.TU.Scaffold_40141_HRSCAF-40491.333	17.74429586	-6.8719941	2.02267158	-3.3974839	0.00068009	0.029625	1.52834164	NA	NA
evm.TU.Scaffold_40164_HRSCAF-40545.287	725.2580944	-1.0960688	0.32257665	-3.3978554	0.00067916	0.029625	1.52834164	probable inactive purple acid phosphatase 2 [Orbicella faveolata]	K22390
evm.TU.Scaffold_40161_HRSCAF-40537.252	1425.041534	-1.2156867	0.35810753	-3.3947553	0.00068691	0.02985956	1.52491665	vulgaris]	NA
evm.TU.Scaffold_40138_HRSCAF-40465.186	67.6803761	-3.6332312	1.07091948	-3.3926278	0.00069226	0.02991744	1.52407553	CG11_big_fil_rev_8_21_14_0_20_36_8]	K03648
evm.TU.Scaffold_743_HRSCAF-861.496	520.0542245	-1.2362283	0.36441795	-3.3923364	0.00069299	0.02991744	1.52407553	NA	NA
evm.TU.Scaffold_743_HRSCAF-861.1958	163.7023893	-1.2356778	0.36412199	-3.393582	0.00068985	0.02991744	1.52407553	PREDICTED- transmembrane protein 136-like [Xenopus laevis]	NA
evm.TU.Scaffold_168_HRSCAF-201.1736	582.1364077	-1.5892501	0.46887485	-3.3894975	0.00070021	0.03012343	1.52109558	vespilloides]	K05108
evm.TU.Scaffold_40109_HRSCAF-40345.105	794.1926874	-0.987246	0.29132374	-3.3888278	0.00070192	0.03013483	1.52093125	NA	NA
evm.TU.Scaffold_40152_HRSCAF-40518.264	16.78517383	-5.798266	1.71484885	-3.3812111	0.00072167	0.03066659	1.51333454	hypothetical protein F511_39717 [Dorcoceras hygrometricum]	K22522
evm.TU.Scaffold_11_HRSCAF-12.1324	532.5986232	-1.5225979	0.45028123	-3.3814375	0.00072108	0.03066659	1.51333454	Dystroglycan [Stylophora pistillata]	K06265
evm.TU.Scaffold_40168_HRSCAF-40561.330	999.3338555	-2.0473746	0.60565837	-3.3804116	0.00072377	0.0306933	1.51295637	uncharacterized protein LOC110254725 [Exaiptasia pallida]	K22866
evm.TU.Scaffold_40140_HRSCAF-40479.137	6.720993525	-5.5409327	1.64156808	-3.3753902	0.00073711	0.03106908	1.50767157	NA	K11593
evm.TU.Scaffold_40109_HRSCAF-40345.92	557.3775153	-1.2116398	0.35944912	-3.3708243	0.00074944	0.03152478	1.50134789	[Apostichopus japonicus]	K05195
evm.TU.Scaffold_40197_HRSCAF-40653.771	8.255610703	-5.7471225	1.70807818	-3.3646718	0.00076635	0.03207297	1.49386077	uncharacterized protein LOC106152497 [Lingula anatina]	NA
evm.TU.Scaffold_40164_HRSCAF-40545.149	483.8149159	-1.3863937	0.41207551	-3.3644166	0.00076706	0.03207297	1.49386077	protein FAM166B [Exaiptasia pallida]	NA
evm.TU.Scaffold_11_HRSCAF-12.1309	840.445173	-1.2606877	0.37471346	-3.3644047	0.00076709	0.03207297	1.49386077	uncharacterized protein LOC111334028 [Stylophora pistillata]	NA
evm.TU.Scaffold_40196_HRSCAF-40652.929	126.1810642	-1.8871682	0.56122815	-3.3625686	0.00077221	0.03210399	1.49344095	NA	NA
evm.TU.Scaffold_2593_HRSCAF-2754.645	1556.517601	-1.1527199	0.34281836	-3.3624801	0.00077246	0.03210399	1.49344095	PREDICTED- uncharacterized protein LOC109311384 [Crocodylus porosus]	K05637
evm.TU.Scaffold_40114_HRSCAF-40359.732	1638.368545	-0.9681317	0.28789815	-3.3627577	0.00077168	0.03210399	1.49344095	sperm-associated antigen 6 [Lingula anatina]	NA
evm.TU.Scaffold_743_HRSCAF-861.1715	259.2051408	-3.4313264	1.02297428	-3.3542645	0.00079576	0.03287573	1.48312466	pistillata]	NA
evm.TU.Scaffold_40122_HRSCAF-40411.224	91.42033434	-2.8617487	0.85303745	-3.354775	0.0007943	0.03287573	1.48312466	NA	NA
evm.TU.Scaffold_73_HRSCAF-87.195	3272.405475	-1.4898268	0.44408661	-3.3548115	0.00079419	0.03287573	1.48312466	ras-related protein Rap-1b-like [Dendronephthya gigantea]	K04353
evm.TU.Scaffold_2593_HRSCAF-2754.115	1080.911959	-1.1555567	0.34470288	-3.3523268	0.00080135	0.03297586	1.4818039	laminin subunit alpha-like [Stylophora pistillata]	K05635
evm.TU.Scaffold_40135_HRSCAF-40458.543	2132.852903	-1.5289415	0.45622623	-3.3512792	0.00080439	0.03298876	1.48163407	uncharacterized protein LOC110043888 [Orbicella faveolata]	NA
evm.TU.Scaffold_40170_HRSCAF-40571.1134	58.92269918	-1.5734557	0.4697317	-3.3496902	0.00080902	0.03309511	1.48023613	uncharacterized protein LOC114949708 [Acropora millepora]	NA
evm.TU.Scaffold_40138_HRSCAF-40465.514	3500.212827	-0.7448883	0.22258315	-3.346562	0.0008182	0.0333398	1.47703698	delta(8)-fatty-acid desaturase [Exaiptasia pallida]	K21734
evm.TU.Scaffold_40168_HRSCAF-40561.831	489.3529654	-1.4573071	0.43607856	-3.3418453	0.00083223	0.03384528	1.47050186	ankyrin repeat and IBR domain-containing protein 1-like [Acropora millepora]	K11967
evm.TU.Scaffold_40196_HRSCAF-40652.1131	664.7134922	-1.199108	0.35890041	-3.3410607	0.00083459	0.03387491	1.47012191	PREDICTED- uncharacterized protein LOC101240982 [Hydra vulgaris]	NA
evm.TU.Scaffold_168_HRSCAF-201.1229	1970.394274	-0.8740724	0.2619191	-3.3371845	0.00084632	0.03428409	1.46490736	PREDICTED- tyrosine-protein phosphatase corkscrew-like [Hydra vulgaris]	K07293
evm.TU.Scaffold_2593_HRSCAF-2754.480	118.7917551	-2.6132024	0.78406366	-3.3328957	0.00085947	0.03474935	1.45905334	cadherin-related tumor suppressor-like [Stylophora pistillata]	K16506
evm.TU.Scaffold_40168_HRSCAF-40561.786	593.8815322	-1.6275194	0.48945506	-3.3251662	0.00088366	0.03558904	1.44868375	PREDICTED- alsin-like [Hydra vulgaris]	K04575
evm.TU.Scaffold_743_HRSCAF-861.588	1262.513306	-1.4146394	0.4257029	-3.3230673	0.00089033	0.03578868	1.44625427	isoform X1 [Branchiostoma belcheri]	K15909
evm.TU.Scaffold_11_HRSCAF-12.501	6.977316341	-4.6905808	1.41200154	-3.3219374	0.00089395	0.03586469	1.44533297	galaxin-like [Stylophora pistillata]	NA
novel_gene_430_5d572bfb	86.51113823	-3.6109653	1.0878972	-3.3192156	0.00090271	0.03614648	1.44193403	NA	NA
evm.TU.Scaffold_40109_HRSCAF-40345.1142	86.29018284	-3.481639	1.04928897	-3.3180936	0.00090634	0.03622233	1.44102359	vulgaris]	NA
evm.TU.Scaffold_40197_HRSCAF-40653.1311	308.1563655	-0.8829044	0.26676258	-3.3097013	0.00093396	0.03711227	1.43048246	NA	NA
evm.TU.Scaffold_40197_HRSCAF-40653.1135	356.2379108	-0.7646007	0.23117251	-3.3074896	0.00094136	0.03726433	1.42870665	PREDICTED- uncharacterized protein LOC100204904 [Hydra vulgaris]	K17586
evm.TU.Scaffold_11_HRSCAF-12.961	24.63398824	-3.4327996	1.03810323	-3.3067998	0.00094368	0.03728533	1.428462	hypothetical protein T12_13943 [Trichinella patagoniensis]	NA
evm.TU.Scaffold_40168_HRSCAF-40561.1139	2064.341163	-1.0888838	0.32936107	-3.3060488	0.00094622	0.0373146	1.42812121	VWFA and cache domain-containing protein 1-like [Pocillopora damicornis]	NA
evm.TU.Scaffold_40152_HRSCAF-40518.917	1341.744953	-1.6139047	0.48902319	-3.3002621	0.00096595	0.03787745	1.4216193	paramyosin-like protein [Aurelia sp. 2017-HT]	NA
evm.TU.Scaffold_743_HRSCAF-861.240	329.3511894	-1.4610902	0.44301864	-3.2980332	0.00097365	0.03805197	1.4196229	NA	NA
evm.TU.Scaffold_40141_HRSCAF-40491.41	331.6044842	-1.012394	0.30765153	-3.2907164	0.00099933	0.03881918	1.41095364	NA	K06792
evm.TU.Scaffold_40170_HRSCAF-40571.693	1225.619551	-1.6316586	0.49622096	-3.2881696	0.00100841	0.03902812	1.40862234	PREDICTED- uncharacterized protein LOC100206956 [Hydra vulgaris]	NA
evm.TU.Scaffold_40140_HRSCAF-40479.188	58.14871889	-2.6717867	0.81298421	-3.2863943	0.00101479	0.0391295	1.40749575	NA	NA
evm.TU.Scaffold_40168_HRSCAF-40561.984	443.966785	-1.3102153	0.39865553	-3.2865852	0.0010141	0.0391295	1.40749575	protein 1-like [Hydra vulgaris]	K07369
evm.TU.Scaffold_40170_HRSCAF-40571.191	1898.535151	-1.5021104	0.45757226	-3.2827828	0.00102788	0.03941525	1.40433574	PREDICTED- protein patched homolog 1-like, partial [Acropora digitifera]	NA
evm.TU.Scaffold_168_HRSCAF-201.1717	13.2067456	-3.3853389	1.03217786	-3.2798018	0.0010388	0.03972071	1.40098298	hypothetical protein AWC38_SpisGene20732 [Stylophora pistillata]	NA
evm.TU.Scaffold_73_HRSCAF-87.694	1176.988697	-1.4346486	0.43745041	-3.2795686	0.00103966	0.03972071	1.40098298	NA	NA
evm.TU.Scaffold_40109_HRSCAF-40345.653	6.120815469	-5.3557899	1.63493897	-3.2758348	0.0010535	0.0401024	1.39682963	NA	NA

novel_gene_480_5d572bfb	5.205465211	-5.5491672	1.69423779	-3.2753178	0.00105543	0.04010255	1.39682802	NA	NA
evm.TU.Scaffold_168_HRSCAF-201.792	5.117133782	-5.7601933	1.75989408	-3.2730341	0.001064	0.04028942	1.39480904	Retrovirus-related Pol polyprotein from transposon 297 [Araneus ventricosus]	NA
evm.TU.Scaffold_40168_HRSCAF-40561.184	795.7653468	-1.4870879	0.45542778	-3.2652553	0.00109366	0.04110483	1.3861071	PREDICTED- tetraspanin-15 [Stegastes partitus]	NA
evm.TU.Scaffold_40196_HRSCAF-40652.1046	337.3334744	-2.7959346	0.85707318	-3.2621889	0.00110556	0.04147722	1.38219034	uncharacterized protein LOC114543425, partial [Dendronephthya gigantea]	NA
evm.TU.Scaffold_743_HRSCAF-861.1196	9.424382351	-4.9700462	1.5266032	-3.2556241	0.00113143	0.04221994	1.37448239	protein phosphatase 1 regulatory subunit 3G [Salvelinus alpinus]	NA
evm.TU.Scaffold_168_HRSCAF-201.566	1270.281386	-1.3663613	0.41992391	-3.2538307	0.0011386	0.04241137	1.37251774	[Strongylocentrotus purpuratus]	K17533
evm.TU.Scaffold_11_HRSCAF-12.1314	386.8838863	-1.7861604	0.54939965	-3.251113	0.00114954	0.04259031	1.37068925	uncharacterized protein LOC111334028 [Stylophora pistillata]	NA
evm.TU.Scaffold_117_HRSCAF-135.368	94.66736354	-1.3634973	0.41934641	-3.251482	0.00114805	0.04259031	1.37068925	uncharacterized protein LOC111339378 [Stylophora pistillata]	NA
evm.TU.Scaffold_40170_HRSCAF-40571.753	246.3938759	-2.3224195	0.7145798	-3.2500491	0.00115385	0.04259837	1.37060703	NA	NA
evm.TU.Scaffold_40152_HRSCAF-40518.916	8834.144268	-0.9625795	0.29615976	-3.2502037	0.00115322	0.04259837	1.37060703	paramyosin-like protein [Aurelia sp. 2017-HT]	NA
evm.TU.Scaffold_40109_HRSCAF-40345.810	6215.200336	-1.2935411	0.39861368	-3.2450996	0.0011741	0.04326906	1.36382253	Tyrosinase [Stylophora pistillata]	NA
evm.TU.Scaffold_40152_HRSCAF-40518.580	5.919801064	-6.342863	1.95549677	-3.243607	0.00118027	0.04331428	1.36336893	protein HEATR9 [Exaiptasia pallida]	NA
evm.TU.Scaffold_40122_HRSCAF-40411.1399	3446.751665	-1.8148205	0.55947056	-3.2438178	0.00117939	0.04331428	1.36336893	adhesion G-protein coupled receptor G4-like, partial [Stylophora pistillata]	K08455
evm.TU.Scaffold_40122_HRSCAF-40411.298	62.49582328	-2.4238522	0.74747087	-3.242738	0.00118387	0.04332258	1.36328571	uncharacterized protein LOC114949039 [Acropora millepora]	NA
evm.TU.Scaffold_40197_HRSCAF-40653.706	3287.461352	-1.3370896	0.41261296	-3.2405421	0.00119303	0.04358106	1.36070226	putative radial spoke head protein 3-like isoform X1 [Apostichopus japonicus]	NA
evm.TU.Scaffold_40196_HRSCAF-40652.750	59.16077026	-2.3079449	0.71304738	-3.2367343	0.00120906	0.04401229	1.35642604	uncharacterized protein LOC113665785 [Pocillopora damicornis]	NA
evm.TU.Scaffold_168_HRSCAF-201.592	350.450822	-1.2091396	0.3739508	-3.233419	0.00122318	0.04426738	1.35391623	damicornis]	NA
evm.TU.Scaffold_2593_HRSCAF-2754.516	322.5186656	-1.062878	0.32865276	-3.2340455	0.0012205	0.04426738	1.35391623	NA	NA
evm.TU.Scaffold_117_HRSCAF-135.230	826.6646072	-1.1409552	0.35319403	-3.2303923	0.00123621	0.04453332	1.35131497	NA	NA
evm.TU.Scaffold_40140_HRSCAF-40479.598	13.06504018	-4.6229773	1.43354144	-3.2248648	0.00126032	0.04508099	1.34600658	NA	NA
evm.TU.Scaffold_40168_HRSCAF-40561.1161	402.6607584	-1.0670271	0.33105484	-3.2231128	0.00126806	0.04513412	1.34549501	UPF0573 protein C2orf70-like [Exaiptasia pallida]	NA
evm.TU.Scaffold_40168_HRSCAF-40561.579	9.486577665	-6.171393	1.91526431	-3.2222148	0.00127204	0.04519857	1.34487532	PREDICTED- uncharacterized protein LOC105847456 [Hydra vulgaris]	NA
evm.TU.Scaffold_40114_HRSCAF-40359.1316	1706.421296	-1.5606993	0.48597239	-3.2114979	0.00132045	0.04644325	1.33307744	PREDICTED- hemicentin-2-like, partial [Acropora digitifera]	K17341
evm.TU.Scaffold_40197_HRSCAF-40653.1270	432.3282027	-1.6247088	0.50613029	-3.2100604	0.00132707	0.04659741	1.33163821	hypothetical protein CAPTEDRAFT_185637 [Capitella teleta]	NA
evm.TU.Scaffold_40122_HRSCAF-40411.600	1971.796126	-1.437771	0.44809008	-3.208665	0.00133353	0.0467453	1.33026202	PREDICTED- uncharacterized protein LOC100206524 [Hydra vulgaris]	NA
evm.TU.Scaffold_40197_HRSCAF-40653.901	188.0262698	-1.132357	0.35335523	-3.2045853	0.00135257	0.04725378	1.32556347	PREDICTED- GPN-loop GTPase 2 [Balearica regulorum gibbericeps]	K06883
evm.TU.Scaffold_40122_HRSCAF-40411.172	28.28866948	-5.1034974	1.59325746	-3.2031844	0.00135917	0.04740473	1.32417831	PREDICTED- hephaestin-like protein [Acropora digitifera]	NA
evm.TU.Scaffold_11_HRSCAF-12.259	170.0231622	-2.7584683	0.86149361	-3.2019603	0.00136496	0.04744768	1.32378506	[Branchiostoma belcheri]	NA
evm.TU.Scaffold_40196_HRSCAF-40652.377	82.02556784	-2.0656564	0.64506792	-3.2022309	0.00136368	0.04744768	1.32378506	vulgaris]	K20412
evm.TU.Scaffold_40197_HRSCAF-40653.1535	858.3519192	-1.2791614	0.39980995	-3.1994237	0.00137703	0.04768453	1.3216225	PREDICTED- cytochrome b-245 heavy chain-like [Hydra vulgaris]	K21421
novel_gene_431_5d572bfb	13.87313231	-3.7696447	1.17860335	-3.1983998	0.00138193	0.04771883	1.3213102	uncharacterized protein LOC114952197 [Acropora millepora]	NA
evm.TU.Scaffold_743_HRSCAF-861.94	3.89466859	-5.518477	1.7257654	-3.1976983	0.00138529	0.04775586	1.32097335	hypothetical protein EGW08_007927 [Elysia chlorotica]	NA
evm.TU.Scaffold_40152_HRSCAF-40518.866	558.9177158	-0.9188805	0.28750371	-3.196065	0.00139316	0.04794764	1.31923279	PREDICTED- uncharacterized protein LOC105845530 [Hydra vulgaris]	NA
evm.TU.Scaffold_40141_HRSCAF-40491.283	124.7939962	-1.5478519	0.48471174	-3.1933452	0.00140635	0.04812718	1.31760958	PREDICTED- tetraspanin-5-like [Hydra vulgaris]	K17346
evm.TU.Scaffold_40138_HRSCAF-40465.879	6246.189001	-0.8491904	0.26592469	-3.1933491	0.00140633	0.04812718	1.31760958	uncharacterized protein LOC114969069 [Acropora millepora]	K08463
evm.TU.Scaffold_11_HRSCAF-12.1568	587.8968099	-0.8126296	0.25449676	-3.1930841	0.00140762	0.04812718	1.31760958	rho GTPase-activating protein 24-like isoform X2 [Orbicella faveolata]	K20642
evm.TU.Scaffold_40170_HRSCAF-40571.759	957.6286738	-4.9890392	1.56281182	-3.192348	0.00141121	0.04816045	1.31730946	NA	NA
evm.TU.Scaffold_40140_HRSCAF-40479.1094	586.6226175	-0.685022	0.21461009	-3.1919376	0.00141322	0.04816045	1.31730946	NA	NA
evm.TU.Scaffold_2593_HRSCAF-2754.201	22.34050894	-2.5043396	0.78507226	-3.189948	0.00142298	0.04837608	1.31536929	Trypsin-containing gene product, partial [Aurelia aurita]	K01310
evm.TU.Scaffold_73_HRSCAF-87.184	2151.917001	-1.4216814	0.44570971	-3.1897026	0.00142419	0.04837608	1.31536929	predicted protein [Nematostella vectensis]	K00966
evm.TU.Scaffold_2593_HRSCAF-2754.182	4348.268075	-1.2196839	0.38276839	-3.1864802	0.00144015	0.04860109	1.313354	PREDICTED- choline transporter-like protein 4 [Hydra vulgaris]	K15377
evm.TU.Scaffold_40114_HRSCAF-40359.1631	1184.777278	-0.9092914	0.28555163	-3.1843326	0.00145088	0.04877	1.31184727	faveolata]	NA
evm.TU.Scaffold_40140_HRSCAF-40479.1086	4215.131868	-2.1182465	0.66585633	-3.1812366	0.00146648	0.04901285	1.30969005	NA	K08814
evm.TU.Scaffold_40164_HRSCAF-40545.539	24.15405097	-2.8461712	0.89499515	-3.1800968	0.00147226	0.04904859	1.30937344	uncharacterized protein LOC114528320 [Dendronephthya gigantea]	NA
evm.TU.Scaffold_117_HRSCAF-135.518	769.5914891	-0.9986994	0.31404319	-3.180134	0.00147207	0.04904859	1.30937344	predicted protein [Nematostella vectensis]	NA
evm.TU.Scaffold_168_HRSCAF-201.584	154.7869726	-1.9417258	0.61127627	-3.176511	0.00149058	0.04934322	1.3067725	PREDICTED- glycine-rich domain-containing protein 1-like [Hydra vulgaris]	NA
evm.TU.Scaffold_40161_HRSCAF-40537.685	2231.956548	-0.7020147	0.22109232	-3.1752108	0.00149728	0.04948619	1.30551596	formin-like protein 13 [Orbicella faveolata]	NA
evm.TU.Scaffold_40114_HRSCAF-40359.1519	21.0275329	-3.2460088	1.02602298	-3.1636805	0.00155788	0.05116423	1.29103355	uncharacterized protein LOC114966573 isoform X2 [Acropora millepora]	NA
evm.TU.Scaffold_40138_HRSCAF-40465.1854	347.1416419	-1.3163128	0.41623765	-3.1624068	0.00156471	0.05122697	1.29050137	PREDICTED- furin-like protease kpc-1 [Branchiostoma belcheri]	K01360
evm.TU.Scaffold_40122_HRSCAF-40411.492	7.334613205	-5.4984073	1.73954724	-3.1608267	0.00157322	0.05130137	1.289871	hypothetical protein X975_10654, partial [Stegodyphus mimosarum]	NA
evm.TU.Scaffold_73_HRSCAF-87.119	342.5909387	-0.9916539	0.3137536	-3.1606136	0.00157437	0.05130137	1.289871	pistillata]	K15980
evm.TU.Scaffold_40135_HRSCAF-40458.96	40.57706728	-8.9606215	2.83789897	-3.1574843	0.00159137	0.05171241	1.28640521	uncharacterized protein LOC114946938 [Acropora millepora]	NA
evm.TU.Scaffold_117_HRSCAF-135.1539	299.1749586	-2.2664748	0.71819752	-3.155782	0.00160068	0.05191503	1.28470691	pistillata]	NA
evm.TU.Scaffold_40164_HRSCAF-40545.718	195.792947	-2.8134728	0.89217543	-3.1534973	0.00161327	0.05216063	1.28265719	vulgaris]	NA
evm.TU.Scaffold_2593_HRSCAF-2754.353	418.9062041	-1.5126178	0.4799595	-3.151553	0.00162405	0.05242775	1.28043876	[Pocillopora damicornis]	NA
evm.TU.Scaffold_40170_HRSCAF-40571.812	29.56733914	-2.8861123	0.91685922	-3.1478249	0.0016449	0.05300612	1.27567403	uncharacterized protein LOC114958815 [Acropora millepora]	NA
evm.TU.Scaffold_40196_HRSCAF-40652.910	36.60651485	-2.5781057	0.81935072	-3.1465228	0.00165224	0.05306239	1.2752132	hypothetical protein EGW08_021985 [Elysia chlorotica]	NA
evm.TU.Scaffold_40141_HRSCAF-40491.627	499.6141197	-0.937613	0.29820646	-3.144174	0.00166556	0.05335441	1.27282966	predicted protein [Nematostella vectensis]	K10260
evm.TU.Scaffold_40122_HRSCAF-40411.78	537.2488991	-0.7260595	0.23099715	-3.1431534	0.00167138	0.05345856	1.27198279	PREDICTED- myosin-IIIa-like [Sinocyclocheilus rhinocerous]	NA
evm.TU.Scaffold_40164_HRSCAF-40545.252	120.250531	-1.3219499	0.42131467	-3.1376782	0.00170292	0.05413456	1.26652537	aspartate aminotransferase, cytoplasmic-like [Stylophora pistillata]	K14454
evm.TU.Scaffold_40138_HRSCAF-40465.472	139.5835238	-1.0447538	0.3333536	-3.1340708	0.00172399	0.0546247	1.26261094	carbohydrate sulfotransferase 5-like [Dendronephthya gigantea]	NA
evm.TU.Scaffold_40109_HRSCAF-40345.1095	18.14646428	-2.6078762	0.83282714	-3.1313535	0.00174003	0.05497848	1.25980731	uncharacterized protein LOC114949346 [Acropora millepora]	NA
evm.TU.Scaffold_11_HRSCAF-12.45	1579.200738	-2.4383549	0.77889307	-3.1305387	0.00174486	0.0550477	1.25926085	uncharacterized protein LOC114536617 [Dendronephthya gigantea]	K13612
evm.TU.Scaffold_40152_HRSCAF-40518.82	177.9172786	-1.9059372	0.60919102	-3.1286364	0.0017562	0.05518301	1.25819464	glutamine-rich protein 2-like isoform X2 [Orbicella faveolata]	NA
evm.TU.Scaffold_40122_HRSCAF-40411.1368	2003.017383	-1.6178654	0.51718967	-3.1281857	0.00175889	0.05518301	1.25819464	PREDICTED- follistatin-like [Hydra vulgaris]	K04661
evm.TU.Scaffold_40164_HRSCAF-40545.804	237.4945026	-1.1407855	0.36469924	-3.1280173	0.0017599	0.05518301	1.25819464	kelch-like protein 2 [Orbicella faveolata]	K10450
evm.TU.Scaffold_40161_HRSCAF-40537.523	2067.466084	-1.0692258	0.34186785	-3.1275997	0.0017624	0.05518301	1.25819464	outer dense fiber protein 3 [Exaiptasia pallida]	NA
evm.TU.Scaffold_40196_HRSCAF-40652.1045	760.3254192	-1.3987827	0.44734877	-3.126828	0.00176703	0.05524498	1.25770718	[Dendronephthya gigantea]	NA
evm.TU.Scaffold_40170_HRSCAF-40571.893	91.39323988	-1.2631013	0.40426469	-3.1244412	0.00178143	0.05548727	1.25580667	NA	NA
evm.TU.Scaffold_40114_HRSCAF-40359.173	499.0907527	-1.553151	0.49744666	-3.1222463	0.00179477	0.05569767	1.25416301	PREDICTED- ankyrin-3-like [Amphimedon queenslandica]	K20032
evm.TU.Scaffold_40197_HRSCAF-40653.1335	2615.902822	-1.6118467	0.51634712	-3.121634	0.0017985	0.05572686	1.25393541	uncharacterized protein LOC113678803 [Pocillopora damicornis]	K16669
evm.TU.Scaffold_40109_HRSCAF-40345.213	203.4228127	-1.126664	0.36109815	-3.1201046	0.00180787	0.05587871	1.25275361	unnamed protein product, partial [Oncorhynchus mykiss]	K10305
evm.TU.Scaffold_743_HRSCAF-861.227	454.5008828	-5.7609848	1.85001078	-3.1140277	0.00184552	0.05684534	1.24530511	allorecognition 1 [Hydractinia symbiolongicarpus]	NA
evm.TU.Scaffold_40168_HRSCAF-40561.785	102.9214895	-1.7173846	0.5522776	-3.1096402	0.00187315	0.05761123	1.23949289	hypothetical protein AC249_AIPGENE28342 [Exaiptasia pallida]	NA
evm.TU.Scaffold_40109_HRSCAF-40345.1050	68.33482997	-2.6216248	0.84392638	-3.1064614	0.00189341	0.05806271	1.23610267	PREDICTED- uncharacterized protein LOC105844767 [Hydra vulgaris]	NA
evm.TU.Scaffold_743_HRSCAF-861.282	13.83910279	-4.7493124	1.53135011	-3.101389	0.00192615	0.05872081	1.231208	NA	NA
evm.TU.Scaffold_40168_HRSCAF-40561.138	372.1156597	-1.3185958	0.42566109	-3.0977598	0.00194989	0.05927108	1.22715715	SPARC-related modular calcium-binding protein 1 isoform X5 [Gallus gallus]	NA
evm.TU.Scaffold_40152_HRSCAF-40518.69	99.72447062	-1.1271061	0.36381319	-3.0980353	0.00194808	0.05927108	1.22715715	protein Njmu-R1 isoform X2 [Lingula anatina]	NA
evm.TU.Scaffold_40170_HRSCAF-40571.1008	1253.801268	-1.0486412	0.33928376	-3.0907499	0.00199652	0.06051162	1.21816123	[Orbicella faveolata]	K02649
evm.TU.Scaffold_40140_HRSCAF-40479.194	926.3260657	-2.9765902	0.96331585	-3.0899421	0.00200196	0.06058824	1.2176117	NA	NA
evm.TU.Scaffold_743_HRSCAF-861.481	53.58364488	-1.8206503	0.5901479	-3.0850746	0.00203501	0.06141014	1.21175991	NA	NA
evm.TU.Scaffold_168_HRSCAF-201.1364	19.75240376	-4.6568069	1.5100942	-3.0837857	0.00204385	0.06158755	1.21050707	NA	NA
evm.TU.Scaffold_40197_HRSCAF-40653.1057	192.402893	-1.8898443	0.61321479	-3.0818636	0.00205709	0.06162985	1.21020888	uncharacterized protein LOC111335634 [Stylophora pistillata]	NA
evm.TU.Scaffold_743_HRSCAF-861.483	35.02780901	-1.6604475	0.53876957	-3.0819251	0.00205667	0.06162985	1.21020888	PREDICTED- zinc finger MYM-type protein 1-like [Nothobranchius furzeri]	NA
evm.TU.Scaffold_40152_HRSCAF-40518.1292	435.2499174	-1.6564616	0.5374066	-3.0823247	0.00205391	0.06162985	1.21020888	dynein regulatory complex protein 10 [Exaiptasia pallida]	NA
evm.TU.Scaffold_40197_HRSCAF-40653.777	11.03924788	-5.8941347	1.91319467	-3.0807815	0.00206458	0.06167677	1.20987841	uncharacterized protein LOC106152497 [Lingula anatina]	NA
evm.TU.Scaffold_40135_HRSCAF-40458.110	21.5632129	-2.5884772	0.84017346	-3.0808842	0.00206387	0.06167677	1.20987841	containing protein 4-like [Acropora digitifera]	NA
evm.TU.Scaffold_168_HRSCAF-201.1608	12.35071089	-5.6069157	1.82112893	-3.0788132	0.00207827	0.06199675	1.20763108	unnamed protein product, partial [Tetraodon nigroviridis]	NA
evm.TU.Scaffold_40140_HRSCAF-40479.232	1074.600847	-0.8027737	0.26188816	-3.0653302	0.0021743	0.06445699	1.19072997	NA	NA
evm.TU.Scaffold_11_HRSCAF-12.1305	9.093920167	-6.5718623	2.14556551	-3.0629977	0.00219132	0.06472001	1.18896144	NA	NA
evm.TU.Scaffold_40196_HRSCAF-40652.277	70.37093138	-9.2534406	3.02192676	-3.0620996	0.0021979	0.06482258	1.18827368	NA	NA
evm.TU.Scaffold_743_HRSCAF-861.743	143.5228441	-1.8273382	0.59706677	-3.0605257	0.00220949	0.06488853	1.18783208	Pogo transposable element with KRAB domain [Stylophora pistillata]	NA
evm.TU.Scaffold_40138_HRSCAF-40465.1761	404.3821656	-1.0128646	0.33093237	-3.0606392	0.00220865	0.06488853	1.18783208	caltractin-like [Stylophora pistillata]	K13448
evm.TU.Scaffold_11_HRSCAF-12.1459	7.618251307	-5.6638358	1.85285857	-3.0568096	0.00223706	0.06532978	1.1848888	uncharacterized protein LOC114954759 [Acropora millepora]	NA

evm.TU.Scaffold_40138_HRSCAF-40465.1791	292.2704055	-3.1538454	1.03204198	-3.0559274	0.00224366	0.06543053	1.18421956	NA	NA
evm.TU.Scaffold_40114_HRSCAF-40359.1317	19.87194883	-5.457975	1.78710984	-3.0540792	0.00225753	0.06565109	1.18275809	NA	NA
evm.TU.Scaffold_40138_HRSCAF-40465.1813_evm.TU.Scaffold_40138_HRSCAF-40465.1814	1114.662545	-0.7802406	0.25552944	-3.0534274	0.00226244	0.06570211	1.18242069	uncharacterized protein LOC110043138 isoform X2 [Orbicella faveolata]	K08843
evm.TU.Scaffold_40135_HRSCAF-40458.617	114.1193324	-2.1192132	0.6943588	-3.0520434	0.00227289	0.06591387	1.18102317	transcriptional regulator Myc-2-like [Pocillopora damicornis]	NA
evm.TU.Scaffold_40122_HRSCAF-40411.1066	72.72526743	-1.9282908	0.63206622	-3.0507733	0.00228253	0.06600945	1.18039391	putative peroxisomal acyl-coenzyme A oxidase 3 [Scophthalmus maximus]	K00232
evm.TU.Scaffold_40114_HRSCAF-40359.155	1005.371234	-1.2266314	0.40218706	-3.0499027	0.00228916	0.06601772	1.18033946	NA	NA
evm.TU.Scaffold_168_HRSCAF-201.758	27.09136689	-2.0660624	0.67826031	-3.0461202	0.00231815	0.06648561	1.17727238	NA	NA
evm.TU.Scaffold_40114_HRSCAF-40359.921	8.777095183	-3.0530858	1.00307803	-3.0437171	0.00233675	0.06692676	1.17440019	uncharacterized protein LOC114530181 [Dendronephthya gigantea]	NA
evm.TU.Scaffold_40197_HRSCAF-40653.807	165.0590657	-2.3928074	0.78806004	-3.0363263	0.0023948	0.06793534	1.16790428	uncharacterized protein LOC111328786 isoform X2 [Stylophora pistillata]	NA
evm.TU.Scaffold_40170_HRSCAF-40571.76	444.2407177	-0.9043344	0.297805	-3.0366663	0.0023921	0.06793534	1.16790428	hypothetical protein [Amycolatopsis sp. CFH S0740]	NA
evm.TU.Scaffold_40170_HRSCAF-40571.811	262.7352214	-1.9258975	0.63442122	-3.0356763	0.00239997	0.06798932	1.16755933	vulgaris]	K17253
evm.TU.Scaffold_40138_HRSCAF-40465.1734	799.5829052	-2.3257947	0.76660187	-3.0339016	0.00241413	0.06811252	1.16677304	uncharacterized protein LOC111330919 [Stylophora pistillata]	K04899
evm.TU.Scaffold_40196_HRSCAF-40652.293	102.5205576	-1.5312989	0.50494846	-3.0325845	0.00242469	0.06820222	1.1662015	uncharacterized protein LOC110253892 [Exaiptasia pallida]	NA
evm.TU.Scaffold_40114_HRSCAF-40359.857	1697.834329	-1.3195392	0.43516401	-3.0322803	0.00242714	0.06820222	1.1662015	predicted protein [Nematostella vectensis]	NA
evm.TU.Scaffold_117_HRSCAF-135.394	146.4549439	-1.0389696	0.34277691	-3.0310373	0.00243715	0.06829928	1.16558387	ubiquitin [Pithovirus LCPAC404]	NA
evm.TU.Scaffold_117_HRSCAF-135.889	3081.492877	-1.2070387	0.39844462	-3.0293763	0.00245059	0.06843719	1.16470784	F-actin-uncapping protein LRRC16A-like [Orbicella faveolata]	K20493
evm.TU.Scaffold_168_HRSCAF-201.247	764.1289328	-0.789225	0.26051498	-3.0294804	0.00244975	0.06843719	1.16470784	bone morphogenetic protein 2-like [Mastacembelus armatus]	K21283
evm.TU.Scaffold_40114_HRSCAF-40359.141	435.3411932	-1.2369609	0.40843099	-3.0285677	0.00245716	0.06849129	1.16436467	hypothetical protein AWC38_SpisGene12281 [Stylophora pistillata]	NA
evm.TU.Scaffold_11_HRSCAF-12.306	188.9552749	-1.5999746	0.52865481	-3.0265015	0.00247402	0.06868528	1.16313633	hypothetical protein BRAFLDRAFT_120347 [Branchiostoma floridae]	K16669
evm.TU.Scaffold_40170_HRSCAF-40571.1105	38.67649954	-4.5417746	1.5029527	-3.0219012	0.00251193	0.06950545	1.15798114	PREDICTED- histamine H2 receptor-like [Saccoglossus kowalevskii]	NA
evm.TU.Scaffold_40152_HRSCAF-40518.1228	63.99387623	-1.770752	0.58601132	-3.0217028	0.00251357	0.06950545	1.15798114	hypothetical protein BSL78_12150 [Apostichopus japonicus]	NA
evm.TU.Scaffold_40170_HRSCAF-40571.854	300.6428405	-1.7767111	0.588618	-3.018445	0.00254076	0.06999795	1.15491467	predicted protein [Nematostella vectensis]	K23732
evm.TU.Scaffold_2593_HRSCAF-2754.308	788.2401675	-1.2730904	0.42172643	-3.0187588	0.00253813	0.06999795	1.15491467	predicted protein [Nematostella vectensis]	NA
evm.TU.Scaffold_743_HRSCAF-861.139	196.0467007	-1.0081964	0.33402127	-3.01836	0.00254147	0.06999795	1.15491467	NA	NA
evm.TU.Scaffold_743_HRSCAF-861.457	71.47472355	-1.1122976	0.36883837	-3.0156775	0.00256406	0.07043379	1.15221892	F-box only protein 36-like [Exaiptasia pallida]	K10312
evm.TU.Scaffold_40168_HRSCAF-40561.881	4.069024334	-5.1861359	1.72020003	-3.0148447	0.00257111	0.07053442	1.15159888	NA	NA
evm.TU.Scaffold_40197_HRSCAF-40653.1174	48.02202134	-1.8690407	0.6202332	-3.0134484	0.00258297	0.07067359	1.15074285	gamma-glutamyl hydrolase-like [Paramormyrops kingsleyae]	K01307
evm.TU.Scaffold_168_HRSCAF-201.1920	159.5467107	-2.144446	0.71185318	-3.0124836	0.00259119	0.07080557	1.14993256	gigantea]	K08076
evm.TU.Scaffold_743_HRSCAF-861.1753	1323.548302	-1.6279197	0.54083556	-3.0100086	0.0026124	0.07101235	1.1486661	sortilin-related receptor-like [Stylophora pistillata]	NA
evm.TU.Scaffold_40114_HRSCAF-40359.856	699.1447237	-1.200451	0.39873038	-3.0106836	0.0026066	0.07101235	1.1486661	NA	NA
evm.TU.Scaffold_2593_HRSCAF-2754.185	923.372878	-0.8945801	0.29726739	-3.0093448	0.00261812	0.0710749	1.14828375	vulgaris]	K05125
evm.TU.Scaffold_40161_HRSCAF-40537.138	789.4513469	-1.2285235	0.40852171	-3.0072417	0.0026363	0.07147533	1.14584381	uncharacterized protein LOC114968325 [Acropora millepora]	K17637
evm.TU.Scaffold_743_HRSCAF-861.140	219.4442436	-1.6718197	0.55612762	-3.0061799	0.00264553	0.07163214	1.14489207	[Sphaeramia orbicularis]	NA
evm.TU.Scaffold_2593_HRSCAF-2754.208	538.5724381	-0.9160092	0.30519479	-3.001392	0.00268748	0.07257948	1.13918612	caspase-8-like isoform X1 [Stylophora pistillata]	K02187
evm.TU.Scaffold_40109_HRSCAF-40345.996	1612.484138	-1.4592092	0.48682955	-2.9973718	0.00272318	0.07335333	1.13458018	E3 ubiquitin-protein ligase MIB1 [Tupaia chinensis]	K10645
evm.TU.Scaffold_168_HRSCAF-201.41	4380.164593	-1.3676023	0.45677504	-2.9940391	0.00275311	0.07406355	1.1303955	hypothetical protein pdam_00007959 [Pocillopora damicornis]	NA
evm.TU.Scaffold_40138_HRSCAF-40465.1901	66.31349555	-3.3349495	1.11411003	-2.9933753	0.0027591	0.07411279	1.13010683	PREDICTED- calmodulin-like protein 3 [Nomascus leucogenys]	NA
evm.TU.Scaffold_40140_HRSCAF-40479.997	585.9910484	-1.1859823	0.39624557	-2.9930488	0.00276206	0.07411279	1.13010683	NA	K17936
evm.TU.Scaffold_11_HRSCAF-12.945	4.491438717	-5.2037335	1.73911178	-2.9921789	0.00276994	0.07422866	1.12942836	hypothetical protein BSL78_14642 [Apostichopus japonicus]	NA
evm.TU.Scaffold_40109_HRSCAF-40345.995	1069.441897	-0.8364587	0.27967791	-2.990793	0.00278254	0.07437491	1.12857358	PREDICTED- E3 ubiquitin-protein ligase MIB1-like [Biomphalaria glabrata]	K10645
evm.TU.Scaffold_11_HRSCAF-12.1527	991.6939542	-1.1907488	0.39820075	-2.9903229	0.00278683	0.07438069	1.12853982	hypothetical protein BRAFLDRAFT_251787 [Branchiostoma floridae]	K08824
evm.TU.Scaffold_40152_HRSCAF-40518.541	154.7055401	-3.1705	1.06075758	-2.9889016	0.00279982	0.07445455	1.12810876	protein DD3-3 [Lingula anatina]	NA
evm.TU.Scaffold_40161_HRSCAF-40537.318	2256.572232	-0.6683277	0.22368927	-2.9877502	0.00281039	0.0745452	1.12758033	PREDICTED- protein unc-13 homolog A-like [Hydra vulgaris]	K15293
evm.TU.Scaffold_40170_HRSCAF-40571.757	7296.100045	-4.7338149	1.58480595	-2.9869997	0.0028173	0.07462639	1.12710754	NA	NA
evm.TU.Scaffold_168_HRSCAF-201.1521	601.3866735	-1.2720605	0.42616266	-2.9849179	0.00283654	0.07495244	1.1252142	hypothetical protein BRAFLDRAFT_80694 [Branchiostoma floridae]	NA
evm.TU.Scaffold_168_HRSCAF-201.1604	6.010835789	-3.6451509	1.22165703	-2.983776	0.00284715	0.07511985	1.12424528	uncharacterized protein LOC114956759 [Acropora millepora]	NA
evm.TU.Scaffold_743_HRSCAF-861.552	141.7754024	-1.8157291	0.60880954	-2.9824256	0.00285974	0.07511985	1.12424528	uncharacterized protein LOC113665785 [Pocillopora damicornis]	NA
evm.TU.Scaffold_743_HRSCAF-861.1797	2254.495672	-1.0792824	0.36189605	-2.9822996	0.00286092	0.07511985	1.12424528	NA	NA
evm.TU.Scaffold_2593_HRSCAF-2754.681	188.9390675	-1.0972562	0.36829744	-2.9792666	0.00288939	0.07577197	1.12049145	PREDICTED- golgin subfamily A member 1-like [Hydra vulgaris]	NA
evm.TU.Scaffold_117_HRSCAF-135.417	1088.944583	-1.6913709	0.56802239	-2.9776483	0.00290469	0.0758864	1.11983603	inositol-3-phosphate synthase-like [Orbicella faveolata]	K01858
evm.TU.Scaffold_40140_HRSCAF-40479.768	830.2329159	-1.0898522	0.36593607	-2.9782584	0.00289892	0.0758864	1.11983603	NA	NA
evm.TU.Scaffold_40197_HRSCAF-40653.1126	513.9533291	-1.0776395	0.36188669	-2.9778368	0.00290291	0.0758864	1.11983603	[Exaiptasia pallida]	NA
evm.TU.Scaffold_40152_HRSCAF-40518.1102	3.793565446	-5.4861603	1.84416672	-2.9748722	0.00293111	0.07619413	1.11807849	NA	NA
evm.TU.Scaffold_40161_HRSCAF-40537.287	242.5947275	-1.5743673	0.52914115	-2.9753257	0.00292678	0.07619413	1.11807849	uncharacterized protein LOC114954074 [Acropora millepora]	NA
evm.TU.Scaffold_40135_HRSCAF-40458.728	342.8683329	-1.3069286	0.43925102	-2.975357	0.00292648	0.07619413	1.11807849	[Dendronephthya gigantea]	K22544
evm.TU.Scaffold_743_HRSCAF-861.622	758.6862576	-0.559537	0.18806985	-2.9751555	0.0029284	0.07619413	1.11807849	NA	NA
evm.TU.Scaffold_168_HRSCAF-201.181	3423.773621	-3.9568255	1.33032518	-2.9743296	0.0029363	0.07623384	1.11785222	pallida]	NA
evm.TU.Scaffold_40196_HRSCAF-40652.398	1952.886954	-1.3580985	0.45770153	-2.9672141	0.00300512	0.0775372	1.11048989	NA	K22614
evm.TU.Scaffold_40114_HRSCAF-40359.786	13.32863435	-4.2247433	1.4244215	-2.9659362	0.00301763	0.0775522	1.11040588	uncharacterized protein LOC114959643 [Acropora millepora]	NA
evm.TU.Scaffold_11_HRSCAF-12.26	319.3395236	-1.4578806	0.49151255	-2.9661106	0.00301592	0.0775522	1.11040588	PREDICTED- probable palmitoyltransferase ZDHHC20 [Hydra vulgaris]	K20028
evm.TU.Scaffold_40168_HRSCAF-40561.364	791.6534829	-1.1612172	0.3915455	-2.9657275	0.00301968	0.0775522	1.11040588	EF-hand domain-containing family member B-like [Stylophora pistillata]	NA
evm.TU.Scaffold_40114_HRSCAF-40359.1551	1318.046006	-1.4843931	0.50109114	-2.9623217	0.00305329	0.07812	1.10723775	uncharacterized protein LOC111334745 [Stylophora pistillata]	NA
evm.TU.Scaffold_40164_HRSCAF-40545.781	4297.869415	-1.4313579	0.48319905	-2.9622531	0.00305397	0.07812	1.10723775	predicted protein [Nematostella vectensis]	NA
evm.TU.Scaffold_40138_HRSCAF-40465.1799	1743.180048	-1.4904319	0.50335936	-2.96097	0.00306672	0.07834995	1.10596128	hypothetical protein C0Q70_04845 [Pomacea canaliculata]	NA
evm.TU.Scaffold_11_HRSCAF-12.384	464.9217798	-1.6132409	0.54537547	-2.9580371	0.00309605	0.07880919	1.10342313	PREDICTED- calpain-15-like [Hydra vulgaris]	K08582
evm.TU.Scaffold_11_HRSCAF-12.296	1079.730979	-0.7732957	0.26156276	-2.9564442	0.00311209	0.07912067	1.10171002	PREDICTED- exocyst complex component 1-like isoform X3 [Hydra vulgaris]	K19983
evm.TU.Scaffold_168_HRSCAF-201.1320	4.451708857	-4.7256868	1.6018295	-2.9501809	0.00317588	0.08035013	1.09501344	hypothetical protein Y032_0507g2688 [Ancylostoma ceylanicum]	NA
evm.TU.Scaffold_40140_HRSCAF-40479.1105	727.4642894	-4.487048	1.52369527	-2.9448461	0.00323115	0.08135318	1.08962546	NA	NA
evm.TU.Scaffold_40122_HRSCAF-40411.464	25.04689321	-3.0329691	1.03210752	-2.9386174	0.0032968	0.0826064	1.08298633	PREDICTED- lumican-like [Xenopus laevis]	NA
evm.TU.Scaffold_117_HRSCAF-135.1578	1604.411308	-1.1928237	0.40604445	-2.9376676	0.00330691	0.08276027	1.08217808	UPF0468 protein C16orf80, partial [Pterocles gutturalis]	NA
evm.TU.Scaffold_40122_HRSCAF-40411.561	326.802219	-1.0957939	0.37306977	-2.937236	0.00331152	0.08277608	1.08209515	NA	NA
evm.TU.Scaffold_40168_HRSCAF-40561.503	7.467803233	-6.2261877	2.12136984	-2.9349846	0.00333564	0.08294346	1.08121784	endonuclease-reverse transcriptase [Bombyx mori]	NA
evm.TU.Scaffold_40168_HRSCAF-40561.1041	53.84479421	-1.8098014	0.61691246	-2.9336438	0.00335008	0.08294346	1.08121784	NA	NA
evm.TU.Scaffold_40197_HRSCAF-40653.823	381.336117	-1.1267051	0.38403091	-2.9338916	0.00334741	0.08294346	1.08121784	PREDICTED- dnaJ homolog subfamily B member 13-like [Acropora digitifera]	K09519
evm.TU.Scaffold_40141_HRSCAF-40491.809	306.396705	-1.0437488	0.35600942	-2.9318011	0.00337003	0.08333807	1.07915658	rab-like protein 2A [Exaiptasia pallida]	K07931
novel_gene_48_5d581bba	30.20664044	-1.7712709	0.60478037	-2.9287837	0.00340291	0.08383183	1.07659105	NA	#N/A
evm.TU.Scaffold_2593_HRSCAF-2754.146	279.5491078	-1.403838	0.47937218	-2.9284929	0.0034061	0.08383183	1.07659105	biogenic amine-like GPCR [Tripedalia cystophora]	NA
evm.TU.Scaffold_40164_HRSCAF-40545.229	4105.88647	-1.0599226	0.36182996	-2.9293391	0.00339684	0.08383183	1.07659105	putative aminopeptidase W07G4.4 [Pocillopora damicornis]	K01255
evm.TU.Scaffold_40164_HRSCAF-40545.824	1128.440919	-1.6067194	0.54975139	-2.9226291	0.0034709	0.08522526	1.06943167	[Phascolarctos cinereus]	NA
evm.TU.Scaffold_40138_HRSCAF-40465.217	628.7719113	-1.0379837	0.35542832	-2.9203744	0.00349611	0.08574326	1.06680002	NA	NA
evm.TU.Scaffold_40152_HRSCAF-40518.753	33.90490942	-3.4768251	1.19117504	-2.9188197	0.00351359	0.08584245	1.06629789	hypothetical protein PHMEG_00031203 [Phytophthora megakarya]	NA
evm.TU.Scaffold_743_HRSCAF-861.226	75.74378317	-3.316185	1.13616034	-2.9187649	0.00351421	0.08584245	1.06629789	NA	NA
evm.TU.Scaffold_168_HRSCAF-201.110	2592.55003	-1.9833113	0.6795539	-2.918549	0.00351665	0.08584245	1.06629789	NA	NA
evm.TU.Scaffold_40164_HRSCAF-40545.120	55.21383688	-1.685505	0.57813822	-2.9154014	0.00355231	0.08661155	1.0624242	PREDICTED- huntingtin-like [Hydra vulgaris]	K04533
evm.TU.Scaffold_40196_HRSCAF-40652.148	458.5416957	-1.2933462	0.44440666	-2.9102764	0.00361109	0.08773648	1.0568198	WD repeat-containing protein 19-like [Pocillopora damicornis]	K19671
evm.TU.Scaffold_40170_HRSCAF-40571.510	3.730705578	-5.1114137	1.75684033	-2.9094356	0.00362082	0.0878282	1.05636603	NA	NA
evm.TU.Scaffold_40170_HRSCAF-40571.883	74.83909575	-3.1091619	1.06872651	-2.9092213	0.0036233	0.0878282	1.05636603	partitus]	K00699
evm.TU.Scaffold_40196_HRSCAF-40652.1171	501.9287057	-1.0995281	0.3780153	-2.9086868	0.0036295	0.08787617	1.0561289	PREDICTED- protein couch potato-like isoform X1 [Hydra vulgaris]	NA
evm.TU.Scaffold_2593_HRSCAF-2754.118	131.0472833	-2.5356745	0.87195334	-2.9080392	0.00363703	0.08795608	1.05573415	laminin subunit gamma-1-like [Orbicella faveolata]	K06245
evm.TU.Scaffold_11_HRSCAF-12.781	761.7956019	-1.180075	0.40602214	-2.9064301	0.00365579	0.08811455	1.0549524	tetratricopeptide repeat protein 29 [Exaiptasia pallida]	NA
evm.TU.Scaffold_117_HRSCAF-135.2	5.638499979	-4.8963711	1.68550087	-2.9049947	0.00367259	0.08834968	1.05379503	uncharacterized protein LOC111319981 [Stylophora pistillata]	NA
evm.TU.Scaffold_117_HRSCAF-135.1277	409.3830181	-1.354192	0.46624754	-2.9044486	0.00367901	0.08835556	1.0537661	ras-related protein Rab-22A-like [Dendronephthya gigantea]	K07891

evm.TU.Scaffold_40164_HRSCAF-40545.87	177.6338887	-1.4565829	0.50168545	-2.9033787	0.0036916	0.08853222	1.05289866	protein SSUH2 homolog [Acropora millepora]	NA
evm.TU.Scaffold_40122_HRSCAF-40411.1461	665.830988	-1.3081131	0.45059154	-2.9031017	0.00369487	0.08853222	1.05289866	solute carrier family 13 member 5-like [Stylophora pistillata]	K14445
evm.TU.Scaffold_40114_HRSCAF-40359.1228	36.95366313	-2.704498	0.93250407	-2.9002533	0.00372861	0.08885605	1.05131301	PREDICTED- melanocortin receptor 3-like [Hydra vulgaris]	NA
evm.TU.Scaffold_40168_HRSCAF-40561.243	597.9653685	-1.4427064	0.497345	-2.9008161	0.00372192	0.08885605	1.05131301	putative N-acetylated-alpha-linked acidic dipeptidase [Orbicella faveolata]	K14592
evm.TU.Scaffold_117_HRSCAF-135.77	919.7178142	-1.0566688	0.3643484	-2.9001604	0.00372972	0.08885605	1.05131301	Nose resistant to fluoxetine protein 6 [Stylophora pistillata]	NA
evm.TU.Scaffold_40161_HRSCAF-40537.234	359.4691873	-0.8551527	0.29503923	-2.8984372	0.00375027	0.08924365	1.04942266	[Anser cygnoides domesticus]	NA
evm.TU.Scaffold_40197_HRSCAF-40653.361	10.37350924	-3.3696917	1.16327658	-2.8967244	0.00377081	0.08962987	1.04754724	hypothetical protein AWC38_SpisGene12197 [Stylophora pistillata]	NA
evm.TU.Scaffold_40196_HRSCAF-40652.515	94.27349965	-1.6512293	0.57025117	-2.8956175	0.00378413	0.08969957	1.04720965	NA	NA
evm.TU.Scaffold_743_HRSCAF-861.1048	215.6671768	-1.4667544	0.50689134	-2.8936269	0.0038082	0.08969957	1.04720965	uncharacterized protein LOC114971463 [Acropora millepora]	NA
evm.TU.Scaffold_40140_HRSCAF-40479.235	1384.524249	-1.4450683	0.4993794	-2.8937283	0.00380697	0.08969957	1.04720965	NA	NA
evm.TU.Scaffold_40138_HRSCAF-40465.697	8.576539846	-4.1383708	1.43158451	-2.8907625	0.00384309	0.09041888	1.04374087	NA	NA
evm.TU.Scaffold_168_HRSCAF-201.1724	415.7576647	-2.8029767	0.97145975	-2.8853246	0.0039101	0.09147884	1.03867934	ephrin type-B receptor 1-B-like [Hydra vulgaris]	NA
evm.TU.Scaffold_40170_HRSCAF-40571.1065	65.63448735	-1.2895636	0.44740321	-2.8823297	0.00394747	0.09193972	1.03649681	Uncharacterized protein K02A2.6 [Stylophora pistillata]	NA
evm.TU.Scaffold_40161_HRSCAF-40537.480	514.6644023	-1.1984431	0.41576994	-2.8824669	0.00394575	0.09193972	1.03649681	vulgaris]	NA
evm.TU.Scaffold_40109_HRSCAF-40345.991	415.9614789	-1.4631878	0.5081352	-2.8795246	0.00398275	0.09245898	1.03405088	mind bomb, putative [Pediculus humanus corporis]	K10645
evm.TU.Scaffold_2593_HRSCAF-2754.348	469.8567742	-1.6250423	0.56443146	-2.8790782	0.00398839	0.09247923	1.03395579	PREDICTED- uncharacterized protein LOC100206524 [Hydra vulgaris]	NA
evm.TU.Scaffold_40141_HRSCAF-40491.807	2925.867024	-1.7324133	0.60281729	-2.8738613	0.00405487	0.09302909	1.03138121	NA	NA
evm.TU.Scaffold_40140_HRSCAF-40479.894	826.5479338	-1.7160301	0.59711361	-2.8738753	0.00405469	0.09302909	1.03138121	NA	NA
evm.TU.Scaffold_40197_HRSCAF-40653.1296	378.7617003	-1.4993389	0.52171445	-2.8738689	0.00405477	0.09302909	1.03138121	PREDICTED- PC3-like endoprotease variant A isoform X2 [Hydra vulgaris]	NA
evm.TU.Scaffold_40122_HRSCAF-40411.923	274.6417366	-1.4420513	0.50180788	-2.873712	0.00405679	0.09302909	1.03138121	[Exaiptasia pallida]	NA
evm.TU.Scaffold_40152_HRSCAF-40518.124	1042.127523	-0.6489704	0.22577093	-2.8744638	0.00404714	0.09302909	1.03138121	PREDICTED- UNC93-like protein MFSD11 [Crassostrea gigas]	NA
evm.TU.Scaffold_40135_HRSCAF-40458.745	1095.324981	-1.612335	0.56135035	-2.8722437	0.00407569	0.09335964	1.02984083	CD151 antigen-like isoform X2 [Dendronephthya gigantea]	K06537
evm.TU.Scaffold_168_HRSCAF-201.366	5.485895336	-5.8175254	2.02673712	-2.8703897	0.00409966	0.09353442	1.02902853	M24 family metallopeptidase [Mesorhizobium sp.]	NA
evm.TU.Scaffold_40164_HRSCAF-40545.1075	117.5517726	-1.2354029	0.43090994	-2.8669631	0.00414431	0.09441235	1.02497119	PREDICTED- gamma-glutamyl hydrolase-like [Hydra vulgaris]	K01307
evm.TU.Scaffold_40109_HRSCAF-40345.62	5598.669964	-0.750584	0.26192978	-2.8655927	0.00416229	0.09455374	1.02432127	tyrosine-protein kinase STK [Hydra vulgaris]	K05705
evm.TU.Scaffold_11_HRSCAF-12.1438	1032.137943	-1.085706	0.37967785	-2.8595452	0.00424249	0.09601861	1.01764458	glycine amidinotransferase, mitochondrial isoform X3 [Loxodonta africana]	K00613
evm.TU.Scaffold_40168_HRSCAF-40561.1082	323.0223171	-1.5524773	0.54356017	-2.8561278	0.00428843	0.09663809	1.01485168	forkhead box protein N2 [Exaiptasia pallida]	K09407
evm.TU.Scaffold_40109_HRSCAF-40345.989	1173.947676	-0.9997742	0.35013161	-2.8554241	0.00429794	0.09674777	1.01435903	belcheri]	K10645
evm.TU.Scaffold_40135_HRSCAF-40458.117	1119.963689	-1.3108053	0.45915279	-2.8548346	0.00430593	0.09675705	1.01431739	PREDICTED- apoptosis regulator BAX-like [Hydra vulgaris]	K02159
evm.TU.Scaffold_40168_HRSCAF-40561.1150	160.8119546	-0.751909	0.26339265	-2.8547077	0.00430765	0.09675705	1.01431739	Jun3 [Aurelia aurita]	NA
evm.TU.Scaffold_40140_HRSCAF-40479.660	222.8488548	-1.3271891	0.46521416	-2.8528562	0.00433282	0.09700864	1.01318959	NA	NA
novel_gene_549_5d572bfb	3.676322537	-4.6977582	1.6470432	-2.8522374	0.00434127	0.09707335	1.01289998	hypothetical protein XELAEV_18013881mg [Xenopus laevis]	NA
evm.TU.Scaffold_40138_HRSCAF-40465.1557	480.5651442	-1.119556	0.39255649	-2.8519614	0.00434504	0.09707335	1.01289998	NA	NA
evm.TU.Scaffold_11_HRSCAF-12.10	7.793278186	-5.6617838	1.98625982	-2.8504749	0.0043654	0.09721534	1.01226519	uncharacterized protein LOC110246883 [Exaiptasia pallida]	NA
evm.TU.Scaffold_40196_HRSCAF-40652.1207	76.97530313	-1.1684507	0.40983728	-2.8510113	0.00435804	0.09721534	1.01226519	beta-mannosidase isoform X1 [Lingula anatina]	K01192
evm.TU.Scaffold_40109_HRSCAF-40345.94	609.7485964	-1.4391133	0.50515168	-2.8488737	0.00438743	0.09742525	1.01132849	Calcium and integrin-binding protein 1 [Stylophora pistillata]	NA
evm.TU.Scaffold_11_HRSCAF-12.713	3113.014703	-0.836944	0.29396607	-2.847077	0.00441227	0.09784053	1.00948121	PREDICTED- testin-like [Hydra vulgaris]	K04511
evm.TU.Scaffold_40138_HRSCAF-40465.1895	2438.003253	-1.2015727	0.42237251	-2.8448175	0.00444369	0.09832779	1.00732373	axin interactor, dorsalization-associated protein isoform X2 [Sus scrofa]	NA
evm.TU.Scaffold_168_HRSCAF-201.49	2757.036682	-1.9999212	0.7035886	-2.8424582	0.00447671	0.09851298	1.00650653	hypothetical protein SODALDRAFT_280356 [Sodiomyces alkalinus F11]	NA
evm.TU.Scaffold_40152_HRSCAF-40518.79	200.5338465	-1.7778895	0.6255345	-2.8421925	0.00448044	0.09851298	1.00650653	[Pocillopora damicornis]	NA
evm.TU.Scaffold_743_HRSCAF-861.1354	7439.046063	-1.3465902	0.47353278	-2.8437106	0.00445915	0.09851298	1.00650653	PREDICTED- filamin-A-like [Hydra vulgaris]	K04437
evm.TU.Scaffold_40122_HRSCAF-40411.1450	421.009171	-0.6378365	0.22438124	-2.8426462	0.00447407	0.09851298	1.00650653	[Acropora digitifera]	K11851
evm.TU.Scaffold_11_HRSCAF-12.1488	1103.126024	-1.4429363	0.50797328	-2.8405753	0.00450322	0.09890942	1.00476236	predicted protein [Nematostella vectensis]	NA
evm.TU.Scaffold_117_HRSCAF-135.1242	9.244953177	-4.3415755	1.52897122	-2.8395404	0.00451786	0.09912627	1.00381124	NA	NA
evm.TU.Scaffold_40138_HRSCAF-40465.1848	209.7188897	-1.5352397	0.54090684	-2.8382701	0.00453588	0.09932257	1.00295203	hypothetical protein CAPTEDRAFT_177803 [Capitella teleta]	K01360
evm.TU.Scaffold_40109_HRSCAF-40345.1078	6.034664278	-4.679274	1.6499777	-2.835962	0.00456879	0.09985667	1.00062294	Otoferlin, partial [Tyto alba]	K19949
evm.TU.Scaffold_40141_HRSCAF-40491.15	1366.332504	0.57422827	0.18956363	3.02921112	0.00245193	0.06843719	1.16470784	queenslandica]	NA
evm.TU.Scaffold_40168_HRSCAF-40561.62	805.7111444	0.63480865	0.21872702	2.90228734	0.00370449	0.0886607	1.05226887	predicted protein [Nematostella vectensis]	NA
evm.TU.Scaffold_40168_HRSCAF-40561.147	657.4785807	0.64011295	0.20605595	3.10650074	0.00189316	0.05806271	1.23610267	uncharacterized protein LOC114520986 [Dendronephthya gigantea]	NA
evm.TU.Scaffold_40114_HRSCAF-40359.777	2007.49456	0.64698347	0.2075894	3.11664986	0.00182919	0.05642567	1.24852327	universal stress protein [Hymenolepis microstoma]	NA
evm.TU.Scaffold_40114_HRSCAF-40359.362	1423.298286	0.65905027	0.22839443	2.88557943	0.00390694	0.09147884	1.03867934	damicornis]	K20879
evm.TU.Scaffold_2593_HRSCAF-2754.980	3260.227178	0.66804844	0.21263989	3.14168914	0.00167976	0.05356205	1.27114279	selenoprotein F-like [Dendronephthya gigantea]	NA
evm.TU.Scaffold_743_HRSCAF-861.854	825.9768489	0.67352191	0.2204967	3.05456688	0.00225386	0.06563612	1.18285713	glycogenin-1 isoform X2 [Exaiptasia pallida]	K00750
evm.TU.Scaffold_117_HRSCAF-135.1387	2579.307636	0.68712909	0.21342355	3.21955611	0.00128389	0.04538745	1.34306426	PREDICTED- core-binding factor subunit beta-like isoform X2 [Hydra vulgaris]	NA
evm.TU.Scaffold_40138_HRSCAF-40465.1780	505.8791842	0.70139035	0.23517327	2.98244084	0.0028596	0.07511985	1.12424528	kowalevskii]	K07943
evm.TU.Scaffold_40140_HRSCAF-40479.108	2763.250476	0.71785089	0.23112937	3.1058402	0.00189739	0.05809927	1.23582936	NA	K10754
evm.TU.Scaffold_40135_HRSCAF-40458.635	551.0193888	0.73447487	0.229589	3.19908563	0.00137864	0.04768453	1.3216225	protein 2-like [Saccoglossus kowalevskii]	K20366
evm.TU.Scaffold_168_HRSCAF-201.1650	589.6169055	0.7481345	0.24652252	3.03475108	0.00240734	0.06802374	1.16733949	F-box/WD repeat-containing protein 7-like [Dendronephthya gigantea]	NA
evm.TU.Scaffold_2593_HRSCAF-2754.488	2232.249726	0.75040896	0.25763877	2.91263993	0.00358388	0.08717694	1.05959836	digitifera]	NA
evm.TU.Scaffold_40122_HRSCAF-40411.155	742.8730622	0.77420551	0.26747396	2.89450797	0.00379753	0.08969957	1.04720965	NA	NA
evm.TU.Scaffold_40140_HRSCAF-40479.1141	382.4976247	0.7818885	0.24437738	3.1995126	0.0013766	0.04768453	1.3216225	NA	K14779
evm.TU.Scaffold_168_HRSCAF-201.388	172.0014816	0.78513145	0.27513901	2.85358097	0.00432295	0.09699621	1.01324522	PREDICTED- cyclin-C-like [Hydra vulgaris]	K15161
evm.TU.Scaffold_40109_HRSCAF-40345.729	1589.510019	0.78623652	0.24176496	3.25206976	0.00114568	0.04259031	1.37068925	vulgaris]	NA
evm.TU.Scaffold_40114_HRSCAF-40359.1360	593.526565	0.78676268	0.26477031	2.97149126	0.00296357	0.07679503	1.11466691	transmembrane protein 245 [Exaiptasia pallida]	NA
evm.TU.Scaffold_40164_HRSCAF-40545.680	5575.994897	0.78712911	0.25807988	3.0499437	0.00228884	0.06601772	1.18033946	DDB_G0290503 [Hydra vulgaris]	K10591
evm.TU.Scaffold_40197_HRSCAF-40653.1575	3399.564882	0.79642789	0.27120385	2.93663933	0.0033179	0.08283604	1.08178066	Cu/Zn superoxide dismutase [Cyanea capillata]	K04565
evm.TU.Scaffold_40161_HRSCAF-40537.26	4439.322579	0.81325149	0.28281581	2.8755517	0.00403322	0.09302909	1.03138121	NA	NA
evm.TU.Scaffold_73_HRSCAF-87.542	811.3762359	0.81630796	0.28710108	2.84327724	0.00446522	0.09851298	1.00650653	uncharacterized protein LOC114951043 [Acropora millepora]	NA
evm.TU.Scaffold_743_HRSCAF-861.615	425.0491534	0.82107214	0.27451431	2.99099942	0.00278066	0.07437491	1.12857358	zinc finger protein ZPR1 [Denticeps clupeoides]	K06874
evm.TU.Scaffold_40161_HRSCAF-40537.87	647.9594129	0.82303409	0.27889349	2.95106956	0.00316676	0.08021678	1.09573479	PREDICTED- death-associated protein kinase 2-like [Hydra vulgaris]	K08803
evm.TU.Scaffold_40170_HRSCAF-40571.701	384.0650015	0.84242639	0.29396862	2.86570175	0.00416086	0.09455374	1.02432127	NA	NA
evm.TU.Scaffold_117_HRSCAF-135.90	410.0488154	0.84289936	0.29268261	2.87990932	0.0039779	0.09245898	1.03405088	3-ketodihydrosphingosine reductase-like [Acropora millepora]	K04708
evm.TU.Scaffold_40168_HRSCAF-40561.728	4128.488843	0.84860098	0.28385146	2.98959528	0.00279347	0.07438069	1.12853982	mitochondrial-like [Amphimedon queenslandica]	K00140
evm.TU.Scaffold_11_HRSCAF-12.327	435.6285635	0.86045253	0.26056581	3.30224643	0.00095914	0.03768144	1.42387249	sepiapterin reductase-like [Dendronephthya gigantea]	K00072
evm.TU.Scaffold_40109_HRSCAF-40345.962	6236.029279	0.86197233	0.26622022	3.2378169	0.00120448	0.04392242	1.35731376	NA	NA
evm.TU.Scaffold_40138_HRSCAF-40465.1718	2644.08604	0.8701499	0.27497461	3.16447358	0.00155364	0.0511056	1.29153153	vulgaris]	K21407
evm.TU.Scaffold_40109_HRSCAF-40345.822	393.2012716	0.87311696	0.30470479	2.86545208	0.00416414	0.09455374	1.02432127	NA	NA
evm.TU.Scaffold_743_HRSCAF-861.1849	368.5597268	0.87378637	0.26093577	3.34866455	0.00081202	0.03315273	1.47948077	predicted protein [Nematostella vectensis]	K10755
evm.TU.Scaffold_2593_HRSCAF-2754.76	547.9780912	0.87606402	0.29729678	2.94676594	0.00321116	0.08094771	1.09179543	[Crassostrea gigas]	NA
evm.TU.Scaffold_40170_HRSCAF-40571.861	795.8868379	0.88887131	0.28489854	3.11995738	0.00180877	0.05587871	1.25275361	uncharacterized protein LOC110062478 [Orbicella faveolata]	K03313
evm.TU.Scaffold_40168_HRSCAF-40561.23	1994.650615	0.90325548	0.29841703	3.02682282	0.00247139	0.06868528	1.16313633	homeobox protein ceh-10-like [Dendronephthya gigantea]	NA
evm.TU.Scaffold_40164_HRSCAF-40545.1177	15495.60215	0.90675855	0.31198793	2.90638983	0.00365626	0.08811455	1.0549524	chymotrypsinogen B-like [Orbicella faveolata]	NA
evm.TU.Scaffold_40138_HRSCAF-40465.292	509.7578994	0.90728165	0.25448147	3.56521687	0.00036356	0.02045933	1.68910864	NA	NA
evm.TU.Scaffold_743_HRSCAF-861.1661	10212.54393	0.90763204	0.25953652	3.4971265	0.0004703	0.02353981	1.62819705	serpin B4-like [Marmota flaviventris]	K23425
evm.TU.Scaffold_40168_HRSCAF-40561.1232	1333.849373	0.91651211	0.31188327	2.93863822	0.00329658	0.0826064	1.08298633	digitifera]	K00288
evm.TU.Scaffold_40197_HRSCAF-40653.763	958.6267829	0.92339394	0.30449065	3.03258552	0.00242468	0.06820222	1.1662015	yessoensis]	K00626
evm.TU.Scaffold_117_HRSCAF-135.266	171.203711	0.92369257	0.26065469	3.54374045	0.00039449	0.02144685	1.66863659	Docking protein 6-like protein [Leptotrombidium deliense]	NA
evm.TU.Scaffold_2593_HRSCAF-2754.734	775.2738108	0.93414157	0.30212087	3.09194654	0.00198849	0.06035608	1.21927901	NA	NA
evm.TU.Scaffold_40141_HRSCAF-40491.115	1606.588303	0.94538915	0.30086185	3.14226994	0.00167643	0.0535379	1.27133868	NA	NA
evm.TU.Scaffold_40138_HRSCAF-40465.733	677.5859031	0.94881053	0.32776235	2.89481243	0.00379385	0.08969957	1.04720965	vulgaris]	NA
evm.TU.Scaffold_40168_HRSCAF-40561.739	2406.369801	0.94906991	0.29433718	3.22443094	0.00126223	0.04508099	1.34600658	PREDICTED- death-associated protein 1 [Monodelphis domestica]	NA
novel_gene_45_5d572bfb	1921.598436	0.95626358	0.33257617	2.87532198	0.00403616	0.09302909	1.03138121	NA	#N/A
evm.TU.Scaffold_40161_HRSCAF-40537.332	159.4778271	0.96624696	0.30321171	3.18670727	0.00143902	0.04860109	1.313354	NA	NA
evm.TU.Scaffold_40138_HRSCAF-40465.558	664.7164432	0.96910218	0.33746197	2.87173744	0.00408222	0.09340657	1.02962256	hypothetical protein BRAFLDRAFT_57355 [Branchiostoma floridae]	NA

evm.TU.Scaffold_40170_HRSCAF-40571.258	440.808408	0.97128248	0.30401594	3.19484059	0.00139908	0.04807205	1.31810735	diphthine methyl ester synthase [Pangasianodon hypophthalmus]	K00586
evm.TU.Scaffold_40164_HRSCAF-40545.872	1204.337866	0.98110752	0.296522	3.30871745	0.00093724	0.03717198	1.42978432	dihydropteridine reductase-like [Stylophora pistillata]	K00357
evm.TU.Scaffold_11_HRSCAF-12.143	3489.474948	0.98215621	0.34060655	2.88355055	0.0039322	0.09182852	1.03702243	hypothetical protein BRAFLDRAFT_117612 [Branchiostoma floridae]	K01587
evm.TU.Scaffold_168_HRSCAF-201.377	136.8877575	1.00563286	0.35287313	2.8498426	0.00437409	0.09730474	1.011866	PREDICTED- protein FAM151B-like isoform X3 [Hydra vulgaris]	NA
evm.TU.Scaffold_40168_HRSCAF-40561.230_evm.TU.Scaffold_40168_HRSCAF-40561.231	2182.762328	1.01021313	0.21979717	4.59611524	4.30E-06	0.0006639	3.17789799	PREDICTED- uncharacterized protein MAL13P1.304-like isoform X2 [Hydra vulgaris]	K10369
evm.TU.Scaffold_40197_HRSCAF-40653.1332	1513.575946	1.0142464	0.35602961	2.84876984	0.00438886	0.09742525	1.01132849	NA	NA
evm.TU.Scaffold_40114_HRSCAF-40359.175	497.3169497	1.01584371	0.35025269	2.90031663	0.00372786	0.08885605	1.05131301	[Pocillopora damicornis]	NA
evm.TU.Scaffold_40114_HRSCAF-40359.1560	259.117321	1.01625742	0.3357624	3.02671597	0.00247226	0.06868528	1.16313633	glutathione S-transferase [Rhodobacteraceae bacterium]	K07393
evm.TU.Scaffold_168_HRSCAF-201.236	1397.482076	1.01898797	0.33577836	3.03470412	0.00240772	0.06802374	1.16733949	PREDICTED- LIM domain-binding protein 3-like isoform X4 [Hydra vulgaris]	NA
evm.TU.Scaffold_743_HRSCAF-861.489	1070.507369	1.01995178	0.33342208	3.05904093	0.00222047	0.06511914	1.18629137	conserved oligomeric Golgi complex subunit 6-like [Stylophora pistillata]	K20293
evm.TU.Scaffold_40138_HRSCAF-40465.369	155.8501069	1.02031558	0.3469525	2.94079325	0.00327373	0.0823256	1.08446508	PREDICTED- uncharacterized protein LOC101236484 [Hydra vulgaris]	NA
evm.TU.Scaffold_40161_HRSCAF-40537.22	9116.075393	1.02247568	0.31374018	3.25898869	0.0011181	0.04179731	1.37885163	canadensis]	K02503
evm.TU.Scaffold_40140_HRSCAF-40479.1081	4993.730919	1.03347062	0.32517233	3.17822431	0.0014818	0.04920902	1.30795532	NA	NA
evm.TU.Scaffold_40109_HRSCAF-40345.221	1107.138999	1.03431692	0.35099318	2.94682914	0.00321051	0.08094771	1.09179543	NA	NA
evm.TU.Scaffold_11_HRSCAF-12.1249	1290.510419	1.0350547	0.35817276	2.88981967	0.00385463	0.09052863	1.04321408	phosphoribosylformylglycinamidine synthase [Exaiptasia pallida]	K01952
evm.TU.Scaffold_40122_HRSCAF-40411.104	522.4878916	1.0404117	0.29420765	3.53631765	0.00040575	0.02183063	1.66093373	RWD domain-containing protein 4-like isoform X1 [Acropora millepora]	NA
evm.TU.Scaffold_73_HRSCAF-87.538	917.7116741	1.04294348	0.34508167	3.02230916	0.00250854	0.06950545	1.15798114	PREDICTED- uncharacterized protein LOC100212051 [Hydra vulgaris]	NA
evm.TU.Scaffold_11_HRSCAF-12.874	9555.277943	1.04331131	0.35561823	2.93379596	0.00334844	0.08294346	1.08121784	NA	NA
evm.TU.Scaffold_743_HRSCAF-861.1919	259.906829	1.04601957	0.30602321	3.41810534	0.00063059	0.02842009	1.54637455	ZP domain-containing protein [Exaiptasia pallida]	NA
evm.TU.Scaffold_40140_HRSCAF-40479.714	1024.774086	1.0491272	0.36313683	2.88906855	0.00386385	0.09060028	1.04287045	NA	K10593
evm.TU.Scaffold_40164_HRSCAF-40545.53	1688.86292	1.05498936	0.3276581	3.21978718	0.00128286	0.04538745	1.34306426	probable small nuclear ribonucleoprotein G [Stylophora pistillata]	K11099
evm.TU.Scaffold_168_HRSCAF-201.134	2257.757073	1.07635772	0.34452057	3.12421903	0.00178278	0.05548727	1.25580667	PREDICTED- prolyl 3-hydroxylase 2-like isoform X2 [Hydra vulgaris]	K08134
evm.TU.Scaffold_40122_HRSCAF-40411.1114	879.2330612	1.0807079	0.36001949	3.00180388	0.00268385	0.07257549	1.13921001	uncharacterized protein LOC111341238 [Stylophora pistillata]	K01921
evm.TU.Scaffold_40122_HRSCAF-40411.1183	383.2882782	1.08567929	0.32145546	3.37738642	0.00073178	0.03090701	1.50994295	E3 ubiquitin-protein ligase RGLG5-like [Stylophora pistillata]	NA
evm.TU.Scaffold_73_HRSCAF-87.17	1355.452418	1.08643414	0.24707048	4.39726402	1.10E-05	0.00141776	2.84839789	matrix metalloproteinase-2-like [Myzus persicae]	K07993
evm.TU.Scaffold_40152_HRSCAF-40518.91	1556.737103	1.08926562	0.30929621	3.52175544	0.0004287	0.02241043	1.64954975	small nuclear ribonucleoprotein 13 L homeolog [Xenopus laevis]	K12845
evm.TU.Scaffold_40114_HRSCAF-40359.636	1647.84246	1.08987882	0.35715347	3.05157002	0.00227648	0.06592609	1.18094271	PREDICTED- cubilin isoform X1 [Crassostrea gigas]	K14616
evm.TU.Scaffold_11_HRSCAF-12.341	9631.111072	1.09072501	0.30103425	3.62325888	0.00029091	0.01740638	1.75929159	NA	NA
evm.TU.Scaffold_40114_HRSCAF-40359.49	2247.623218	1.09161496	0.27091825	4.02931499	5.59E-05	0.00506424	2.29548589	PREDICTED- collagen alpha-1(XXVI) chain-like [Acropora digitifera]	NA
evm.TU.Scaffold_40197_HRSCAF-40653.936	2448.325398	1.09309753	0.23428329	4.66570841	3.08E-06	0.00050425	3.29735754	vulgaris]	K03126
evm.TU.Scaffold_117_HRSCAF-135.650	284.3216369	1.09418882	0.28825225	3.795942	0.00014708	0.01071171	1.97014107	PREDICTED- lysophosphatidylcholine acyltransferase 2-like [Hydra vulgaris]	K13510
evm.TU.Scaffold_40197_HRSCAF-40653.977	493.0486492	1.09617357	0.31260312	3.50659827	0.00045387	0.02299406	1.63838426	hypothetical protein pdam_00010848, partial [Pocillopora damicornis]	K17198
evm.TU.Scaffold_11_HRSCAF-12.632	2146.306255	1.10363558	0.28217574	3.91116399	9.19E-05	0.00738436	2.1316869	fibrillin-1-like [Dendronephthya gigantea]	NA
evm.TU.Scaffold_11_HRSCAF-12.694	94.91809519	1.10412647	0.36219448	3.04843535	0.00230036	0.06615771	1.17941955	PREDICTED- uncharacterized protein LOC105844041 [Hydra vulgaris]	NA
evm.TU.Scaffold_40141_HRSCAF-40491.149	1924.813537	1.1193318	0.3571924	3.13369436	0.00172621	0.0546247	1.26261094	[Apis dorsata]	K08765
evm.TU.Scaffold_40114_HRSCAF-40359.598	10206.4772	1.12168426	0.33833804	3.31527678	0.00091552	0.03644942	1.43830938	PREDICTED- collagen alpha-1(III) chain-like [Hydra vulgaris]	K19720
evm.TU.Scaffold_40152_HRSCAF-40518.888	242.3939873	1.12329857	0.38793116	2.89561313	0.00378419	0.08969957	1.04720965	NA	NA
evm.TU.Scaffold_40140_HRSCAF-40479.858	315.5850299	1.14959923	0.39721843	2.89412359	0.00380219	0.08969957	1.04720965	NA	K14801
evm.TU.Scaffold_73_HRSCAF-87.813	143.0009744	1.15192835	0.40133175	2.87026466	0.00410128	0.09353442	1.02902853	probable ATP-dependent DNA helicase HFM1 [Enhydra lutris kenyoni]	K15271
evm.TU.Scaffold_40170_HRSCAF-40571.687	794.868187	1.15534364	0.28775444	4.0150332	5.94E-05	0.00533451	2.27290555	cystinosin-like [Stylophora pistillata]	K12386
evm.TU.Scaffold_40197_HRSCAF-40653.1197	3157.677772	1.17252949	0.34568057	3.39194506	0.00069398	0.02991744	1.52407553	NA	NA
evm.TU.Scaffold_73_HRSCAF-87.153	72.13045547	1.17432678	0.37443683	3.13624809	0.00171124	0.05431634	1.26506952	protein MON2 homolog [Pocillopora damicornis]	NA
evm.TU.Scaffold_40168_HRSCAF-40561.1185	2784.182068	1.17529427	0.36328412	3.23519312	0.00121561	0.04417334	1.35483977	uncharacterized protein LOC114961713 isoform X1 [Acropora millepora]	K10404
evm.TU.Scaffold_40138_HRSCAF-40465.528	284.2205844	1.18445026	0.36253934	3.26709442	0.00108657	0.04091257	1.38814325	uncharacterized protein LOC114946779 [Acropora millepora]	NA
evm.TU.Scaffold_40161_HRSCAF-40537.352	94.40767989	1.18544957	0.35006756	3.38634516	0.0007083	0.03034624	1.51789514	gigas]	K03426
evm.TU.Scaffold_743_HRSCAF-861.1447	384.5319059	1.20131249	0.33851505	3.54877132	0.00038703	0.02117256	1.67422669	HAUS augmin-like complex subunit 5 [Pocillopora damicornis]	K04638
evm.TU.Scaffold_2593_HRSCAF-2754.518	280.8111156	1.20443119	0.41954119	2.87082944	0.00409396	0.09353442	1.02902853	[Chelonia mydas]	K09008
evm.TU.Scaffold_40141_HRSCAF-40491.923	997.2292493	1.2068223	0.36382128	3.31707458	0.00090965	0.03628506	1.44027215	PREDICTED- uncharacterized protein LOC100207339 [Hydra vulgaris]	NA
evm.TU.Scaffold_40170_HRSCAF-40571.122	1314.255657	1.21168127	0.35102509	3.45183663	0.00055679	0.0261112	1.5831732	girdin-like [Pocillopora damicornis]	NA
evm.TU.Scaffold_40152_HRSCAF-40518.266	209.5367286	1.21724062	0.38896121	3.12946535	0.00175125	0.05516561	1.25833155	[Biomphalaria glabrata]	NA
evm.TU.Scaffold_117_HRSCAF-135.299	1989.679088	1.21915366	0.27787711	4.3873842	1.15E-05	0.00144772	2.83931453	jaculus]	K02437
evm.TU.Scaffold_40161_HRSCAF-40537.31	534.7618181	1.22707974	0.41302855	2.97093202	0.00296898	0.07679503	1.11466691	PREDICTED- cartilage oligomeric matrix protein-like [Hydra vulgaris]	K04659
evm.TU.Scaffold_40114_HRSCAF-40359.62	686.4920821	1.23765087	0.41658272	2.97096065	0.0029687	0.07679503	1.11466691	NA	NA
evm.TU.Scaffold_40140_HRSCAF-40479.1040	1075.966206	1.24080424	0.24757381	5.01185591	5.39E-07	0.0001207	3.91831072	NA	NA
evm.TU.Scaffold_117_HRSCAF-135.1223	104.2853515	1.24676726	0.36160844	3.44783779	0.00056509	0.02632298	1.57966491	pistillata]	NA
evm.TU.Scaffold_117_HRSCAF-135.653	138.913646	1.2471179	0.42479151	2.93583526	0.00332651	0.08286202	1.08164451	transmembrane protein 229A [Lingula anatina]	NA
evm.TU.Scaffold_40140_HRSCAF-40479.757	1938.515957	1.24955121	0.37970033	3.2908879	0.00099872	0.03881918	1.41095364	NA	NA
evm.TU.Scaffold_168_HRSCAF-201.1737	3228.115901	1.25952984	0.2883377	4.36824546	1.25E-05	0.00154315	2.81159101	Kazal-type serine proteinase inhibitor 1, partial [Apostichopus japonicus]	NA
evm.TU.Scaffold_40161_HRSCAF-40537.516	909.7465626	1.26438595	0.40496289	3.12222668	0.00179489	0.05569767	1.25416301	growth arrest-specific protein 1-like [Orbicella faveolata]	NA
evm.TU.Scaffold_40140_HRSCAF-40479.834	2583.928955	1.27019035	0.26226742	4.84311148	1.28E-06	0.00024874	3.60425786	NA	NA
evm.TU.Scaffold_40170_HRSCAF-40571.47	4606.696058	1.27365623	0.33723902	3.77671668	0.00015891	0.01129287	1.94719583	selenoprotein Pb-like [Pocillopora damicornis]	NA
evm.TU.Scaffold_40109_HRSCAF-40345.1156	271.3343213	1.27451138	0.44105752	2.8896716	0.00385645	0.09052863	1.04321408	uridine-cytidine kinase-like 1 [Exaiptasia pallida]	K00876
evm.TU.Scaffold_40164_HRSCAF-40545.874	819.7932641	1.28658183	0.3917704	3.28402001	0.00102338	0.0393877	1.40463936	trichohyalin-like [Dendronephthya gigantea]	NA
evm.TU.Scaffold_40168_HRSCAF-40561.229	3743.462562	1.30099556	0.4246248	3.06387086	0.00218493	0.06462309	1.18961229	sacsin-like [Orbicella faveolata]	K17592
evm.TU.Scaffold_40164_HRSCAF-40545.1079	2204.611073	1.30511848	0.3704214	3.52333443	0.00042615	0.02241043	1.64954975	hypothetical protein EI555_004596 [Monodon monoceros]	K05738
evm.TU.Scaffold_743_HRSCAF-861.1663	70.5636201	1.32500485	0.41061046	3.22691452	0.00125133	0.04484537	1.3482824	PREDICTED- uncharacterized protein LOC105844503 [Hydra vulgaris]	NA
evm.TU.Scaffold_743_HRSCAF-861.22	159.6671434	1.34430123	0.40389515	3.32834207	0.00087365	0.03525396	1.45279214	NA	NA
evm.TU.Scaffold_2593_HRSCAF-2754.365	6657.377407	1.34677963	0.2857064	4.71385881	2.43E-06	0.00041485	3.38210786	332-1 secreted propeptide [Malo kingi]	NA
evm.TU.Scaffold_40122_HRSCAF-40411.1221	318.1737043	1.34847788	0.32745143	4.1181005	3.82E-05	0.00368249	2.43385878	queenslandica]	K00799
evm.TU.Scaffold_40161_HRSCAF-40537.773	110.7935742	1.35232289	0.30843966	4.3844002	1.16E-05	0.00145887	2.8359843	potassium channel subfamily K member 3-like [Stylophora pistillata]	K04914
evm.TU.Scaffold_40164_HRSCAF-40545.968	3218.015327	1.35371759	0.43905053	3.08328428	0.0020473	0.06160227	1.21040326	PREDICTED- lysyl oxidase homolog 4-like [Acropora digitifera]	K00280
evm.TU.Scaffold_40140_HRSCAF-40479.1010	1831.226765	1.37562922	0.45698167	3.01025034	0.00261032	0.07101235	1.1486661	NA	NA
evm.TU.Scaffold_117_HRSCAF-135.1653	144.1794248	1.39328493	0.45507898	3.06163324	0.00220133	0.06483181	1.18821183	hypothetical protein OCBIM_22028330mg, partial [Octopus bimaculoides]	K06971
evm.TU.Scaffold_117_HRSCAF-135.329	1558.691781	1.39597038	0.40679432	3.43163688	0.00059995	0.0273951	1.56232714	NA	NA
evm.TU.Scaffold_168_HRSCAF-201.1432	772.8864935	1.42098787	0.40838652	3.47951709	0.00050232	0.02443755	1.6119423	[Exaiptasia pallida]	K06255
evm.TU.Scaffold_2593_HRSCAF-2754.1073	83.40974259	1.42502601	0.33817434	4.21387971	2.51E-05	0.00272243	2.5650436	[Dendronephthya gigantea]	NA
evm.TU.Scaffold_40164_HRSCAF-40545.1016	867.7107667	1.42732193	0.465364	3.06710859	0.0021614	0.06420077	1.19245978	[Strongylocentrotus purpuratus]	NA
evm.TU.Scaffold_40152_HRSCAF-40518.1127	88932.00032	1.42900866	0.41214988	3.46720629	0.0005259	0.02511525	1.60006243	NA	NA
evm.TU.Scaffold_40109_HRSCAF-40345.408	58.46913789	1.43403542	0.4534236	3.16268372	0.00156322	0.05122697	1.29050137	zinc finger protein 271-like [Octopus vulgaris]	NA
evm.TU.Scaffold_11_HRSCAF-12.1253	2090.053969	1.44158938	0.33565412	4.29486574	1.75E-05	0.00203145	2.69219493	Pancreatic Triacylglyceride Lipase-like protein 1, partial [Aurelia aurita]	K14073
evm.TU.Scaffold_168_HRSCAF-201.1827	296.5747533	1.44385524	0.50518897	2.85804978	0.00426254	0.09626302	1.01654051	vulgaris]	NA
evm.TU.Scaffold_117_HRSCAF-135.378	561.8426127	1.44560383	0.45947177	3.14622993	0.0016539	0.05306239	1.2752132	PREDICTED- glutathione synthetase-like [Hydra vulgaris]	K21456
evm.TU.Scaffold_168_HRSCAF-201.1691	154.3315239	1.45004207	0.42650956	3.39978796	0.00067438	0.02950427	1.53011516	BTB/POZ domain-containing protein 6-like [Dendronephthya gigantea]	K10478
evm.TU.Scaffold_40170_HRSCAF-40571.1148	1933.230031	1.46577195	0.4904846	2.98841585	0.00280428	0.07447788	1.12797269	NA	NA
evm.TU.Scaffold_743_HRSCAF-861.494	39740.94851	1.49274652	0.36695121	4.06797007	4.74E-05	0.00435341	2.36117033	follistatin-related protein 1 [Mus caroli]	NA
evm.TU.Scaffold_168_HRSCAF-201.1824	2626.486854	1.49914329	0.36138885	4.14828315	3.35E-05	0.0033908	2.46969795	PLOD3 isoform 14, partial [Pongo abelii]	K13646
evm.TU.Scaffold_40197_HRSCAF-40653.299	869.6466512	1.53044109	0.50323693	3.04119392	0.00235642	0.0673975	1.17135622	hypothetical protein AWC38_SpisGene17943, partial [Stylophora pistillata]	NA
evm.TU.Scaffold_11_HRSCAF-12.317	702.0523591	1.53489795	0.4057516	3.78285121	0.00015504	0.011106	1.95444245	Sulfotransferase 1C4 [Papilio xuthus]	K01025
evm.TU.Scaffold_40140_HRSCAF-40479.1054	557.5921666	1.56257033	0.51547616	3.03131446	0.00243492	0.06829928	1.16558387	NA	NA
evm.TU.Scaffold_40114_HRSCAF-40359.1131	45.67716614	1.56692208	0.54965001	2.85076333	0.00436144	0.09721534	1.01226519	neurexin-4 [Lingula anatina]	K07380
evm.TU.Scaffold_40138_HRSCAF-40465.134	109.5415561	1.57329541	0.51760406	3.03957317	0.00236914	0.06766826	1.16961501	hypothetical protein EGW08_003010 [Elysia chlorotica]	NA
evm.TU.Scaffold_40197_HRSCAF-40653.1205	116.9876402	1.57393403	0.49517199	3.17856032	0.00148008	0.04920902	1.30795532	NA	NA

evm.TU.Scaffold_40135_HRSCAF-40458.53	100.5250365	1.58232609	0.52501385	3.01387497	0.00257934	0.07066715	1.15078242	uncharacterized protein LOC114516711 [Dendronephthya gigantea]	NA
evm.TU.Scaffold_40114_HRSCAF-40359.799	42.3800312	1.62378037	0.55440485	2.92887115	0.00340195	0.08383183	1.07659105	PREDICTED- uncharacterized protein LOC105844874 [Hydra vulgaris]	NA
evm.TU.Scaffold_40114_HRSCAF-40359.631	26416.82192	1.63079425	0.53336281	3.05757023	0.00223139	0.06532978	1.1848888	collagen-like protein [Acetobacterium wieringae]	NA
evm.TU.Scaffold_40170_HRSCAF-40571.947	231.4502132	1.63612939	0.43981708	3.72002244	0.00019921	0.01335733	1.87428044	phospholipid phosphatase 2 [Exaiptasia pallida]	K01080
evm.TU.Scaffold_168_HRSCAF-201.1814	61.52495673	1.63854398	0.45407678	3.60851741	0.00030795	0.0182164	1.7395374	Sulfotransferase family cytosolic 1B member [Daphnia magna]	K01025
evm.TU.Scaffold_40114_HRSCAF-40359.227	200065.0504	1.63981564	0.50719694	3.23309448	0.00122457	0.04426738	1.35391623	PREDICTED- collagen alpha-1(I) chain-like [Hydra vulgaris]	NA
evm.TU.Scaffold_40122_HRSCAF-40411.1442	1089.476838	1.64330765	0.4734772	3.47072182	0.00051906	0.02490299	1.60374854	nitric oxide synthase, endothelial-like isoform X2 [Acropora millepora]	K13240
evm.TU.Scaffold_40161_HRSCAF-40537.496	532.7237035	1.65958745	0.55967779	2.96525516	0.00302432	0.0775522	1.11040588	PREDICTED- galactose-3-O-sulfotransferase 2-like [Hydra vulgaris]	K01019
evm.TU.Scaffold_40168_HRSCAF-40561.56	198.9957413	1.68317191	0.40802354	4.12518334	3.70E-05	0.00358758	2.44519865	neurexin-4 [Parasteatoda tepidariorum]	NA
evm.TU.Scaffold_40141_HRSCAF-40491.142	305.2864821	1.68565713	0.51163398	3.29465435	0.00098543	0.03842431	1.41539388	NA	NA
evm.TU.Scaffold_40114_HRSCAF-40359.597	78.95651305	1.69082399	0.51186561	3.30325766	0.00095569	0.03761679	1.42461831	PREDICTED- uncharacterized protein LOC107335479 [Acropora digitifera]	NA
evm.TU.Scaffold_40135_HRSCAF-40458.741	1078.358405	1.69193025	0.40997433	4.12691752	3.68E-05	0.00358758	2.44519865	hypothetical protein EPR50_G00076050 [Perca flavescens]	K09377
evm.TU.Scaffold_40122_HRSCAF-40411.1373	950.5160378	1.69859632	0.5718776	2.97020956	0.00297597	0.07688037	1.11418454	NA	NA
evm.TU.Scaffold_11_HRSCAF-12.499	639.468374	1.70135618	0.54844798	3.10212863	0.00192135	0.05866017	1.23165668	NA	NA
evm.TU.Scaffold_40138_HRSCAF-40465.692	163.9454566	1.70900978	0.45181096	3.78257709	0.00015521	0.011106	1.95444245	histamine N-methyltransferase A-like [Stylophora pistillata]	K00546
evm.TU.Scaffold_40170_HRSCAF-40571.783	4761.18334	1.7207042	0.5647401	3.04689573	0.00231218	0.06640578	1.17779411	uncharacterized protein LOC112576500 isoform X4 [Pomacea canaliculata]	K12382
evm.TU.Scaffold_40122_HRSCAF-40411.1139	735.3266657	1.7216676	0.45012578	3.82485888	0.00013085	0.0098221	2.00779587	PREDICTED- cytospin-A-like isoform X2 [Hydra vulgaris]	K23028
evm.TU.Scaffold_40161_HRSCAF-40537.542	17365.97546	1.72299673	0.43617788	3.95021571	7.81E-05	0.00652931	2.18513271	PREDICTED- alpha-2-macroglobulin-like, partial [Hydra vulgaris]	NA
evm.TU.Scaffold_40114_HRSCAF-40359.837	270017.9966	1.77836181	0.42331397	4.20104684	2.66E-05	0.00285158	2.54491459	fibrillar collagen, partial [Hydra vulgaris]	K19719
evm.TU.Scaffold_117_HRSCAF-135.492	223.366315	1.77973868	0.55831575	3.18769205	0.00143413	0.04857768	1.31356326	bivittatum]	K03327
evm.TU.Scaffold_2593_HRSCAF-2754.720	302.4669092	1.78584758	0.54477687	3.27812663	0.00104499	0.03985106	1.39956012	PREDICTED- uncharacterized protein LOC100206524 [Hydra vulgaris]	NA
evm.TU.Scaffold_40152_HRSCAF-40518.660	38.11051115	1.79248936	0.62353431	2.87472451	0.0040438	0.09302909	1.03138121	NA	NA
evm.TU.Scaffold_40114_HRSCAF-40359.534	25738.30594	1.7996513	0.48933396	3.6777568	0.00023529	0.01489147	1.82706234	uncharacterized protein LOC113669629 [Pocillopora damicornis]	K17341
evm.TU.Scaffold_40114_HRSCAF-40359.166	117.5675909	1.80722801	0.47441215	3.80940501	0.0001393	0.01024925	1.98930787	sine oculis, partial [Aurelia sp. 1 NN-2010a]	NA
evm.TU.Scaffold_40122_HRSCAF-40411.1170	210.0793424	1.81077154	0.53493242	3.38504732	0.00071166	0.03042748	1.51673406	PREDICTED- aflatoxin B1 aldehyde reductase member 2-like [Hydra vulgaris]	K15303
evm.TU.Scaffold_117_HRSCAF-135.1619	301.6690252	1.81227698	0.62937242	2.87949859	0.00398308	0.09245898	1.03405088	cytochrome P450 3A14-like [Pocillopora damicornis]	K07424
evm.TU.Scaffold_73_HRSCAF-87.855	63.18613938	1.81345672	0.57485809	3.15461632	0.00160709	0.05204182	1.28364752	digitifera]	K10838
evm.TU.Scaffold_11_HRSCAF-12.198	504.1837478	1.82006834	0.61912411	2.93974716	0.0032848	0.08250439	1.08352293	predicted protein [Nematostella vectensis]	K13366
evm.TU.Scaffold_40161_HRSCAF-40537.472	1906.46055	1.82290729	0.6111187	2.98290217	0.00285529	0.07511985	1.12424528	vulgaris]	K00315
evm.TU.Scaffold_40109_HRSCAF-40345.1001	3198.650157	1.82657234	0.39689994	4.60209783	4.18E-06	0.00064992	3.18713943	intraflagellar transport protein 172 homolog [Lingula anatina]	K19676
evm.TU.Scaffold_40164_HRSCAF-40545.221	52.95615247	1.83284513	0.62778734	2.91953184	0.00350558	0.08584245	1.06629789	solute carrier family 46 member 3-like [Pocillopora damicornis]	K14613
evm.TU.Scaffold_40161_HRSCAF-40537.543	322.5136555	1.84049825	0.58229102	3.16078761	0.00157343	0.05130137	1.289871	PREDICTED- alpha-2-macroglobulin-like isoform X2 [Hydra vulgaris]	NA
evm.TU.Scaffold_40161_HRSCAF-40537.891	147.9030769	1.85281153	0.36742404	5.04270637	4.59E-07	0.00010985	3.95919203	peptidyl-prolyl cis-trans isomerase 6-like [Orbicella faveolata]	K03768
evm.TU.Scaffold_11_HRSCAF-12.584	3994.564853	1.85493109	0.56709497	3.27093555	0.00107192	0.04043404	1.3932529	protostelioides]	NA
evm.TU.Scaffold_40135_HRSCAF-40458.799	114.0954142	1.85507379	0.47231693	3.92760383	8.58E-05	0.00697833	2.15624856	hypothetical protein Y032_0053g2350 [Ancylostoma ceylanicum]	NA
evm.TU.Scaffold_117_HRSCAF-135.211	119.4331143	1.87545118	0.35197189	5.32841182	9.91E-08	3.54E-05	4.45099674	PREDICTED- solute carrier family 22 member 15-like [Hydra vulgaris]	K08211
evm.TU.Scaffold_168_HRSCAF-201.1739	1282.651731	1.87554598	0.51479708	3.6432724	0.00026919	0.01643739	1.78416706	gigantea]	NA
evm.TU.Scaffold_168_HRSCAF-201.72	10007.55218	1.886632	0.47846851	3.94306413	8.04E-05	0.00667359	2.17564074	S-adenosylmethionine synthase-like [Orbicella faveolata]	K00789
evm.TU.Scaffold_40170_HRSCAF-40571.1167	358.3345513	1.91723906	0.59389907	3.22822371	0.00124562	0.04479482	1.34877222	PREDICTED- solute carrier family 22 member 15-like [Hydra vulgaris]	K08202
evm.TU.Scaffold_73_HRSCAF-87.602	720.50303	1.91992024	0.51712609	3.71267329	0.00020508	0.01364284	1.86509531	NA	NA
evm.TU.Scaffold_40114_HRSCAF-40359.630	231610.288	1.92256434	0.37741993	5.09396613	3.51E-07	8.90E-05	4.05060999	collagen alpha-1(I) chain-like precursor [Hydra vulgaris]	K19719
evm.TU.Scaffold_117_HRSCAF-135.449	13096.29301	1.92707861	0.32143578	5.99522124	2.03E-09	1.92E-06	5.71669877	NA	NA
evm.TU.Scaffold_40138_HRSCAF-40465.1961	97.42947207	1.94536479	0.51346717	3.78868384	0.00015145	0.01094944	1.96060793	pistillata]	NA
evm.TU.Scaffold_168_HRSCAF-201.624	128.0546232	1.9454376	0.66936605	2.90638819	0.00365628	0.08811455	1.0549524	uncharacterized protein LOC110051864 [Orbicella faveolata]	NA
evm.TU.Scaffold_40197_HRSCAF-40653.1465	285.1510899	1.95365745	0.60796227	3.21345177	0.0013115	0.04628477	1.33456186	PREDICTED- NADPH oxidase 4-like isoform X1 [Hydra vulgaris]	K21423
evm.TU.Scaffold_2593_HRSCAF-2754.175	179.0109936	1.97681158	0.42898453	4.60811861	4.06E-06	0.00063613	3.19645139	NA	NA
evm.TU.Scaffold_168_HRSCAF-201.1580	162.9453071	2.02830468	0.5504254	3.68497655	0.00022872	0.01460885	1.835384	uncharacterized protein LOC114951874 [Acropora millepora]	NA
evm.TU.Scaffold_40170_HRSCAF-40571.871	40.22495761	2.02848	0.71016379	2.85635515	0.00428536	0.09663809	1.01485168	gremlin292 precursor [Saccoglossus kowalevskii]	K23318
evm.TU.Scaffold_40122_HRSCAF-40411.1433	71.61372993	2.04265587	0.52083121	3.92191525	8.78E-05	0.0070898	2.14936589	Mnx, partial [Sepia officinalis]	NA
evm.TU.Scaffold_40122_HRSCAF-40411.1222	435.0783148	2.05383054	0.44760641	4.58847432	4.46E-06	0.0006836	3.16519795	queenslandica]	K00799
evm.TU.Scaffold_40138_HRSCAF-40465.1300	3235.467278	2.12629793	0.6152747	3.45585143	0.00054856	0.02590038	1.58669391	PREDICTED- protein mab-21-like 2 [Acropora digitifera]	NA
evm.TU.Scaffold_40122_HRSCAF-40411.97	13.31457732	2.12738953	0.72503289	2.93419729	0.00334412	0.08294346	1.08121784	NA	NA
evm.TU.Scaffold_168_HRSCAF-201.1581	8774.802725	2.13736238	0.58412394	3.65909051	0.00025311	0.01577933	1.80191139	NA	NA
evm.TU.Scaffold_168_HRSCAF-201.690	124.8857148	2.14463192	0.43888274	4.88657158	1.03E-06	0.00020743	3.68313681	NA	NA
evm.TU.Scaffold_40114_HRSCAF-40359.229	79755.61505	2.14977759	0.36876493	5.82966926	5.55E-09	3.96E-06	5.40230481	collagen alpha-1(I) chain-like precursor [Hydra vulgaris]	K19720
evm.TU.Scaffold_40114_HRSCAF-40359.228	23190.41348	2.16064343	0.39748765	5.43574986	5.46E-08	2.32E-05	4.63451202	NA	NA
evm.TU.Scaffold_40141_HRSCAF-40491.276	109.2446573	2.16194441	0.72306359	2.98997824	0.00278997	0.07438069	1.12853982	[Takifugu rubripes]	NA
evm.TU.Scaffold_40168_HRSCAF-40561.576	76.51302162	2.19182342	0.75247833	2.91280602	0.00358197	0.08717694	1.05959836	hypothetical protein BOX15_Mlig005960g1 [Macrostomum lignano]	NA
evm.TU.Scaffold_168_HRSCAF-201.1552	119.8283967	2.19237018	0.62875439	3.48684673	0.00048875	0.02423043	1.61563886	PREDICTED- homeobox protein aristaless-like 4 [Branchiostoma belcheri]	K09451
evm.TU.Scaffold_2593_HRSCAF-2754.602	181.4182734	2.20655709	0.43149051	5.11380209	3.16E-07	8.32E-05	4.07987667	vitamin K-dependent protein C-like isoform X2 [Stylophora pistillata]	K01314
evm.TU.Scaffold_40122_HRSCAF-40411.296	332.0340768	2.21128231	0.70035421	3.15737706	0.00159195	0.05171241	1.28640521	TNF receptor-associated factor 3-like [Stylophora pistillata]	K03174
evm.TU.Scaffold_2593_HRSCAF-2754.1092	1286.723105	2.24713557	0.52496521	4.2805419	1.86E-05	0.00214477	2.66861988	protein 1-like [Pocillopora damicornis]	NA
evm.TU.Scaffold_40197_HRSCAF-40653.753	20.35280405	2.25766266	0.70899311	3.18432245	0.00145093	0.04877	1.31184727	hypothetical protein pdam_00008052 [Pocillopora damicornis]	NA
evm.TU.Scaffold_40122_HRSCAF-40411.1596	222.0406848	2.263074	0.50942551	4.44240412	8.90E-06	0.0012028	2.91980694	papilin-like isoform X1 [Pocillopora damicornis]	NA
evm.TU.Scaffold_40114_HRSCAF-40359.78	5403.641862	2.28783454	0.47073289	4.86015446	1.17E-06	0.0002326	3.63339029	blue pigment protein precursor [Rhizostoma pulmo]	NA
evm.TU.Scaffold_2593_HRSCAF-2754.1618	40.62785056	2.29864719	0.77511902	2.96554094	0.00302151	0.0775522	1.11040588	hypothetical protein Y032_0188g1142 [Ancylostoma ceylanicum]	NA
evm.TU.Scaffold_40109_HRSCAF-40345.559	54.58574633	2.31563835	0.7827768	2.95823581	0.00309405	0.07880919	1.10342313	MAC/Perforin domain containing protein [Rhopilema esculentum]	NA
evm.TU.Scaffold_40138_HRSCAF-40465.1491	1646.065149	2.31615339	0.72899834	3.17717236	0.00148719	0.04930922	1.30707185	uncharacterized protein LOC110055427 [Orbicella faveolata]	NA
evm.TU.Scaffold_40109_HRSCAF-40345.670	29.37661181	2.33530061	0.66285609	3.52308841	0.00042655	0.02241043	1.64954975	pallida]	NA
evm.TU.Scaffold_40138_HRSCAF-40465.57	12808.78842	2.33536072	0.41759044	5.5924669	2.24E-08	1.20E-05	4.92081875	hypothetical protein pdam_00018053 [Pocillopora damicornis]	K23603
evm.TU.Scaffold_40141_HRSCAF-40491.273	3129.561377	2.36241381	0.35857077	6.58841722	4.45E-11	7.12E-08	7.14752001	myosin essential light chain [Aurelia sp. 2017-HT]	K12751
evm.TU.Scaffold_40197_HRSCAF-40653.1236	274.5980309	2.37382312	0.7487864	3.17022734	0.0015232	0.05026309	1.2987508	hydroxylase MINA-like [Hydra vulgaris]	K21760
evm.TU.Scaffold_40114_HRSCAF-40359.1490	51.7220406	2.40264833	0.68092146	3.5285249	0.00041788	0.022244	1.65278704	Precursor	NA
evm.TU.Scaffold_2593_HRSCAF-2754.831	178.1612638	2.45910384	0.81494468	3.01751013	0.00254861	0.07010179	1.15427087	mesoglein, partial [Aurelia sp. 1 LSA-2014]	K19899
evm.TU.Scaffold_40138_HRSCAF-40465.668	273.9664286	2.47029452	0.7371535	3.35112638	0.00080484	0.03298876	1.48163407	histamine N-methyltransferase-like [Stylophora pistillata]	NA
evm.TU.Scaffold_11_HRSCAF-12.629	266.3478055	2.47735673	0.69594086	3.55972306	0.00037125	0.0207798	1.6823586	containing protein 1-like, partial [Hydra vulgaris]	NA
evm.TU.Scaffold_40109_HRSCAF-40345.786	1239.143847	2.48245877	0.72692425	3.41501714	0.00063778	0.02856602	1.54415033	transmembrane protease serine 9-like [Stylophora pistillata]	K09637
evm.TU.Scaffold_168_HRSCAF-201.1998	119.3476675	2.48299241	0.77981648	3.18407275	0.00145219	0.04877	1.31184727	NA	NA
evm.TU.Scaffold_40114_HRSCAF-40359.1140	63.07646574	2.48954133	0.7825532	3.18130618	0.00146613	0.04901285	1.30969005	PREDICTED- proton-coupled folate transporter-like [Hydra vulgaris]	K20840
evm.TU.Scaffold_40122_HRSCAF-40411.799	62.88354483	2.49493862	0.80381063	3.10388857	0.00190995	0.05839799	1.2336021	uncharacterized protein LOC103519065 [Diaphorina citri]	NA
evm.TU.Scaffold_73_HRSCAF-87.266	657.2292166	2.51214549	0.28869381	8.7017642	3.27E-18	1.70E-14	13.7695511	carboxypeptidase B-like [Pocillopora damicornis]	K08637
evm.TU.Scaffold_40122_HRSCAF-40411.197	15.73599558	2.52211617	0.80295362	3.14104838	0.00168344	0.0535973	1.27085709	uncharacterized protein LOC112684782 [Sipha flava]	NA
evm.TU.Scaffold_40170_HRSCAF-40571.776_evm.TU.Scaffold_40170_HRSCAF-40571.778	25.03599516	2.53423777	0.79647652	3.181811	0.00146357	0.04901285	1.30969005	uncharacterized protein LOC111321123 [Stylophora pistillata]	K04988
evm.TU.Scaffold_40168_HRSCAF-40561.344	29.86489583	2.56289774	0.66795682	3.8369213	0.00012459	0.00953727	2.02057592	PREDICTED- uncharacterized protein LOC107332814 [Acropora digitifera]	NA
evm.TU.Scaffold_40114_HRSCAF-40359.606	78.78014593	2.56787522	0.68807685	3.73195994	0.00019	0.01297079	1.88703357	PREDICTED- uncharacterized protein LOC107328045 [Acropora digitifera]	NA
evm.TU.Scaffold_40196_HRSCAF-40652.50	50.00225579	2.57431475	0.6239767	4.12565847	3.70E-05	0.00358758	2.44519865	uncharacterized protein LOC112056533 [Bicyclus anynana]	NA
evm.TU.Scaffold_11_HRSCAF-12.1682	238.9042152	2.57800057	0.6970297	3.69855199	0.00021683	0.0140651	1.85185733	NA	NA
evm.TU.Scaffold_40161_HRSCAF-40537.822	84.09974881	2.5917628	0.54316715	4.77157498	1.83E-06	0.00033135	3.47971958	PREDICTED- uncharacterized protein LOC107332814 [Acropora digitifera]	NA
evm.TU.Scaffold_40122_HRSCAF-40411.312	82.41383026	2.61763181	0.70255973	3.72584951	0.00019466	0.01315967	1.88075487	NA	NA
evm.TU.Scaffold_40109_HRSCAF-40345.441	147.6365161	2.6350058	0.7311308	3.60401423	0.00031334	0.01832686	1.73691187	hypothetical protein BOX15_Mlig031494g1 [Macrostomum lignano]	NA
evm.TU.Scaffold_168_HRSCAF-201.1570	1416.166424	2.66290206	0.83540488	3.18755865	0.00143479	0.04857768	1.31356326	NA	NA
evm.TU.Scaffold_40114_HRSCAF-40359.1610	88.50716006	2.68162699	0.7308041	3.66941973	0.0002431	0.01524657	1.81682773	retinol dehydrogenase 8-like [Pocillopora damicornis]	K11150

evm.TU.Scaffold_40138_HRSCAF-40465.1303	1481.740202	2.70049848	0.64883089	4.16209912	3.15E-05	0.00328296	2.48373481	PREDICTED- lysyl oxidase homolog 2B-like [Hydra vulgaris]	K00280
evm.TU.Scaffold_40197_HRSCAF-40653.1546	2142.612419	2.70181008	0.65702112	4.1122119	3.92E-05	0.00370903	2.43074012	persalinus]	NA
novel_gene_54_5d572bfb	82.57204856	2.74509278	0.91197064	3.01006707	0.0026119	0.07101235	1.1486661	NA	#N/A
evm.TU.Scaffold_40140_HRSCAF-40479.1053	3067.601613	2.78147172	0.59806381	4.65079427	3.31E-06	0.00053417	3.27231807	NA	K09609
evm.TU.Scaffold_2593_HRSCAF-2754.1202	24.91765506	2.78883908	0.93377158	2.98663951	0.00282062	0.07462639	1.12710754	stimulated by retinoic acid gene 6 protein-like [Stylophora pistillata]	NA
evm.TU.Scaffold_168_HRSCAF-201.1093	12.67459604	2.80354624	0.85391025	3.28318609	0.00102641	0.03941525	1.40433574	unnamed protein product (mitochondrion) [Plasmodiophora brassicae]	NA
evm.TU.Scaffold_40138_HRSCAF-40465.1174	12.76035295	2.82147566	0.99121322	2.84648711	0.00442045	0.09791771	1.00913874	hypothetical protein DMG74_22350, partial [Acidobacteria bacterium]	NA
evm.TU.Scaffold_40109_HRSCAF-40345.813	918.4858712	2.82580832	0.65615243	4.30663391	1.66E-05	0.00194995	2.70997742	Tyrosinase [Stylophora pistillata]	NA
evm.TU.Scaffold_40161_HRSCAF-40537.554	93.2053409	2.83431996	0.82674802	3.42827548	0.00060743	0.02751279	1.5604653	retinol dehydrogenase 8-like [Dendronephthya gigantea]	K11150
evm.TU.Scaffold_2593_HRSCAF-2754.595	32820.31464	2.84641732	0.68749177	4.14029292	3.47E-05	0.00348907	2.45729044	von Willebrand factor A domain-containing protein 7-like [Exaiptasia pallida]	NA
evm.TU.Scaffold_117_HRSCAF-135.666	164.0366841	2.84784711	0.78388708	3.63298132	0.00028017	0.01695815	1.77062163	PREDICTED- zinc metalloproteinase nas-15-like [Hydra vulgaris]	NA
evm.TU.Scaffold_168_HRSCAF-201.1569	25.62903395	2.87042897	0.98815707	2.90483067	0.00367452	0.08834968	1.05379503	uncharacterized protein LOC111324678 [Stylophora pistillata]	NA
evm.TU.Scaffold_40114_HRSCAF-40359.684	22.57783497	2.8954405	0.95288065	3.03861822	0.00237666	0.06775722	1.16904442	uncharacterized protein LOC110040580 [Orbicella faveolata]	NA
evm.TU.Scaffold_40114_HRSCAF-40359.761	160.0226409	2.97978252	1.01498174	2.93579913	0.0033269	0.08286202	1.08164451	LamG domain-containing protein, partial [Nodularia spumigena]	NA
evm.TU.Scaffold_40197_HRSCAF-40653.779	47.40592849	2.98410293	0.92575901	3.22341225	0.00126673	0.04513412	1.34549501	uncharacterized protein LOC114541496 [Dendronephthya gigantea]	NA
evm.TU.Scaffold_40114_HRSCAF-40359.68	143.574045	2.99932328	0.63788336	4.70199326	2.58E-06	0.00043614	3.36037906	predicted protein [Nematostella vectensis]	K10864
evm.TU.Scaffold_168_HRSCAF-201.1090	140.3048789	3.00942077	1.04369966	2.88341646	0.00393387	0.09182852	1.03702243	uncharacterized protein LOC110246338 [Exaiptasia pallida]	NA
evm.TU.Scaffold_73_HRSCAF-87.389	43.13372884	3.04106737	0.57889516	5.25322651	1.49E-07	4.45E-05	4.35163999	hypothetical protein B5V51_1814 [Heliothis virescens]	NA
evm.TU.Scaffold_40152_HRSCAF-40518.402	68.77492062	3.0477958	0.74088894	4.11370132	3.89E-05	0.00370199	2.43156464	NA	NA
evm.TU.Scaffold_168_HRSCAF-201.1303	203.4324125	3.08444284	0.77491802	3.9803473	6.88E-05	0.00589654	2.2294029	formosus]	K00069
evm.TU.Scaffold_40140_HRSCAF-40479.753	852.1427517	3.10902111	0.57326289	5.42337758	5.85E-08	2.36E-05	4.627088	NA	NA
evm.TU.Scaffold_40161_HRSCAF-40537.239	106.0598061	3.11270797	1.01151239	3.07728113	0.00208898	0.06222715	1.20602006	[Anser cygnoides domesticus]	K08888
evm.TU.Scaffold_743_HRSCAF-861.1805	30.26810264	3.13869698	0.95172121	3.29791638	0.00097405	0.03805197	1.4196229	belcheri]	NA
evm.TU.Scaffold_11_HRSCAF-12.1471	14.4455908	3.14935863	0.90474245	3.48094493	0.00049965	0.02441767	1.61229574	hypothetical protein EGW08_011329 [Elysia chlorotica]	NA
evm.TU.Scaffold_11_HRSCAF-12.1766	18.08915685	3.16174553	0.88143466	3.58704469	0.00033445	0.01929046	1.71465742	sculpturatus]	NA
evm.TU.Scaffold_40141_HRSCAF-40491.680	50.06593851	3.17522025	0.84425602	3.76096845	0.00016926	0.01182639	1.92714773	NA	NA
evm.TU.Scaffold_40164_HRSCAF-40545.925	24.25526246	3.2277398	1.13818959	2.83585426	0.00457033	0.09985667	1.00062294	Trypsin-3 [Stylophora pistillata]	K01315
evm.TU.Scaffold_40138_HRSCAF-40465.1355	102.4031488	3.24361115	0.50260317	6.45362256	1.09E-10	1.62E-07	6.79048499	hypothetical protein [Pantoea deleyi]	NA
evm.TU.Scaffold_40135_HRSCAF-40458.46	39.5897762	3.31044216	1.15578645	2.86423341	0.0041802	0.09471175	1.02359615	uncharacterized protein LOC113800461 [Penaeus vannamei]	NA
evm.TU.Scaffold_40135_HRSCAF-40458.583	1150.828062	3.33657297	0.84814197	3.93397932	8.36E-05	0.00684976	2.16432484	hypothetical protein Saspl_018281 [Salvia splendens]	NA
evm.TU.Scaffold_40114_HRSCAF-40359.1611	110.7653326	3.38998704	0.89751859	3.77706611	0.00015869	0.01129287	1.94719583	retinol dehydrogenase 8-like [Dendronephthya gigantea]	K11150
evm.TU.Scaffold_40140_HRSCAF-40479.631	82.39333755	3.43118566	0.82720021	4.14795065	3.35E-05	0.0033908	2.46969795	NA	K03948
evm.TU.Scaffold_40197_HRSCAF-40653.113	37.57831604	3.47773525	1.09806602	3.16714586	0.00153943	0.0507184	1.29483446	uncharacterized protein LOC110067018 [Orbicella faveolata]	NA
evm.TU.Scaffold_40138_HRSCAF-40465.1852	516.8566213	3.49120526	0.72274271	4.83049527	1.36E-06	0.00026258	3.58074332	uncharacterized protein LOC113683922 [Pocillopora damicornis]	NA
evm.TU.Scaffold_11_HRSCAF-12.320	330.2791448	3.61938487	0.91711974	3.94646929	7.93E-05	0.00660575	2.1800782	[Nasonia vitripennis]	K00994
evm.TU.Scaffold_117_HRSCAF-135.1623	347.8484853	3.6634218	1.03211087	3.549446	0.00038604	0.02117256	1.67422669	cytochrome P450 3A8-like [Stylophora pistillata]	NA
evm.TU.Scaffold_40170_HRSCAF-40571.45	143.9676467	3.68818677	1.14106023	3.23224548	0.00122822	0.04432217	1.35337899	probable dimethyladenosine transferase [Acanthaster planci]	K14191
evm.TU.Scaffold_40109_HRSCAF-40345.521	88.93275602	3.71348435	0.86020718	4.31696505	1.58E-05	0.00189248	2.7229687	uncharacterized protein LOC113671488 [Pocillopora damicornis]	K07806
evm.TU.Scaffold_40114_HRSCAF-40359.1231	93.94171574	3.74125252	0.95851503	3.90317564	9.49E-05	0.00753817	2.12273396	PREDICTED- uncharacterized protein LOC107341959 [Acropora digitifera]	NA
evm.TU.Scaffold_40141_HRSCAF-40491.649	1424.455575	3.75015149	0.70783479	5.29806046	1.17E-07	3.87E-05	4.41228904	NA	NA
evm.TU.Scaffold_40138_HRSCAF-40465.396	45.27185042	3.77850894	1.15513656	3.27104958	0.00107149	0.04043404	1.3932529	PREDICTED- 26S protease regulatory subunit 4 [Hydra vulgaris]	K03062
evm.TU.Scaffold_40196_HRSCAF-40652.1258	9.929546353	3.80665727	1.28933334	2.9524229	0.00315291	0.07996328	1.09710942	endothelin-converting enzyme 1-like [Orbicella faveolata]	K08635
evm.TU.Scaffold_40122_HRSCAF-40411.106	13.34887782	3.81920204	1.34562465	2.83823728	0.00453635	0.09932257	1.00295203	[Strongylocentrotus purpuratus]	K17973
evm.TU.Scaffold_743_HRSCAF-861.1129	71.96114577	3.87355617	1.09226014	3.54636779	0.00039058	0.02128971	1.67183017	NA	NA
evm.TU.Scaffold_40168_HRSCAF-40561.958	19.87114269	3.90778481	1.07180293	3.64599192	0.00026636	0.01631234	1.78748363	hypothetical protein C7M84_003485 [Penaeus vannamei]	NA
evm.TU.Scaffold_40114_HRSCAF-40359.1656	25.89565457	3.96464266	0.92727801	4.27557065	1.91E-05	0.00216922	2.6636956	PREDICTED- homeobox protein DLX-1-like isoform X1 [Hydra vulgaris]	K18488
evm.TU.Scaffold_11_HRSCAF-12.1727	35.86557507	4.02018608	0.92234859	4.35864068	1.31E-05	0.00160296	2.79507704	NA	NA
evm.TU.Scaffold_40196_HRSCAF-40652.1137	290.7861932	4.02291792	1.40721262	2.85878472	0.00425267	0.09614458	1.01707521	millepora]	K00512
evm.TU.Scaffold_40114_HRSCAF-40359.849	21.30090398	4.02637362	1.31714214	3.05690138	0.00223638	0.06532978	1.1848888	Tigger transposable element-derived protein 3 [Acipenser ruthenus]	NA
evm.TU.Scaffold_40138_HRSCAF-40465.1946	97.4695989	4.05516145	1.19904288	3.38199868	0.00071961	0.03066659	1.51333454	NA	NA
evm.TU.Scaffold_40140_HRSCAF-40479.899	18.94837234	4.16519438	1.19000471	3.5001495	0.000465	0.02337994	1.63115663	NA	NA
evm.TU.Scaffold_743_HRSCAF-861.342	146.2544858	4.19299736	1.14322293	3.6676988	0.00024474	0.01530344	1.81521108	PREDICTED- gamma-tubulin complex component 3 homolog [Hydra vulgaris]	NA
evm.TU.Scaffold_40197_HRSCAF-40653.1410	7.706653605	4.24620382	1.45060297	2.92719917	0.0034203	0.08408198	1.07529706	damicornis]	K07852
evm.TU.Scaffold_40197_HRSCAF-40653.65	6.697883012	4.26118825	1.49914206	2.84241792	0.00447728	0.09851298	1.00650653	[Strongylocentrotus purpuratus]	NA
evm.TU.Scaffold_743_HRSCAF-861.32	83.47732991	4.29106724	0.8721993	4.91982422	8.66E-07	0.00017858	3.74817205	leukocyte elastase inhibitor isoform X2 [Scleropages formosus]	K13963
evm.TU.Scaffold_11_HRSCAF-12.1448	78.87603165	4.3144061	0.85377452	5.05333203	4.34E-07	0.00010892	3.96289636	solute carrier family 46 member 3-like [Pocillopora damicornis]	K20840
evm.TU.Scaffold_11_HRSCAF-12.897	108.066675	4.32349068	0.92047651	4.6970136	2.64E-06	0.0004433	3.35330717	uncharacterized protein LOC114950140 [Acropora millepora]	NA
evm.TU.Scaffold_743_HRSCAF-861.178	10.37218956	4.33148211	1.42624141	3.03699086	0.00238953	0.06793534	1.16790428	NA	NA
evm.TU.Scaffold_40140_HRSCAF-40479.1197	287.961792	4.38301618	0.72121354	6.07727937	1.22E-09	1.34E-06	5.8728952	NA	NA
evm.TU.Scaffold_40151_HRSCAF-40517.5	30.35856781	4.46206189	1.04354027	4.27588855	1.90E-05	0.00216922	2.6636956	hypothetical protein AWC38_SpisGene24559 [Stylophora pistillata]	NA
evm.TU.Scaffold_40141_HRSCAF-40491.732	36.06330323	4.5455554	1.23862423	3.66984214	0.0002427	0.01524657	1.81682773	[Branchiostoma belcheri]	NA
evm.TU.Scaffold_2593_HRSCAF-2754.157	56.69656654	4.55640841	1.44765413	3.14744269	0.00164705	0.05300612	1.27567403	NA	NA
evm.TU.Scaffold_40197_HRSCAF-40653.776	307.9770849	4.56244433	0.82980825	5.49819112	3.84E-08	1.74E-05	4.75945075	uncharacterized protein LOC110067018 [Orbicella faveolata]	K07497
evm.TU.Scaffold_40122_HRSCAF-40411.229	93.32892385	4.56365999	1.2326601	3.70228581	0.00021367	0.01396025	1.85510696	NA	NA
evm.TU.Scaffold_40170_HRSCAF-40571.814	15.20337788	4.59547037	1.53245179	2.99876994	0.00271072	0.07311217	1.13601034	uncharacterized protein K02A2.6-like [Dendronephthya gigantea]	NA
evm.TU.Scaffold_40122_HRSCAF-40411.309	11.54908986	4.59592855	1.58792059	2.89430629	0.00379997	0.08969957	1.04720965	NA	NA
evm.TU.Scaffold_40168_HRSCAF-40561.836	60.96907315	4.64074496	1.34211698	3.45777979	0.00054465	0.02581475	1.58813216	PREDICTED- regucalcin-like [Branchiostoma belcheri]	K01053
evm.TU.Scaffold_168_HRSCAF-201.174	7.053178734	4.64656958	1.57014037	2.95933388	0.00308305	0.07867064	1.10418732	neuronal acetylcholine receptor subunit alpha-10-like [Stylophora pistillata]	K04805
evm.TU.Scaffold_11_HRSCAF-12.1209	112.7274456	4.77897902	1.16588447	4.09901592	4.15E-05	0.00387411	2.41182794	high-mobility group box 2a-like [Scleropages formosus]	K11295
evm.TU.Scaffold_743_HRSCAF-861.607	17.33571724	4.84177037	1.64283317	2.94720759	0.00320658	0.08094771	1.09179543	NA	NA
evm.TU.Scaffold_2593_HRSCAF-2754.369	5.975949651	4.94506905	1.71839552	2.87772459	0.00400555	0.09271435	1.03285303	meristosporus CBS 931.73]	NA
evm.TU.Scaffold_40122_HRSCAF-40411.185	8.098143857	4.98341805	1.68762407	2.95291952	0.00314784	0.07993211	1.09727873	arginine kinase 1 [Tropiometra macrodiscus]	K00933
evm.TU.Scaffold_40141_HRSCAF-40491.77	23.98568241	5.04835959	1.42134763	3.55181202	0.00038259	0.02117256	1.67422669	NA	NA
evm.TU.Scaffold_40109_HRSCAF-40345.288	144.7093965	5.07124869	1.01024202	5.01983544	5.17E-07	0.00011833	3.9268978	NA	NA
evm.TU.Scaffold_743_HRSCAF-861.354	119.1339272	5.07209728	1.17559299	4.31450114	1.60E-05	0.00189248	2.7229687	methylosome subunit pICln-like [Dendronephthya gigantea]	K05019
evm.TU.Scaffold_40152_HRSCAF-40518.1163	7.298610925	5.08102881	1.43187593	3.54851192	0.00038741	0.02117256	1.67422669	predicted protein [Nematostella vectensis]	NA
novel_gene_566_5d572bfb	7.684324636	5.09128	1.44787226	3.51638757	0.00043746	0.02254661	1.64691871	hypothetical protein CRYPA_887 [uncultured SUP05 cluster bacterium]	NA
evm.TU.Scaffold_11_HRSCAF-12.573	552.4106897	5.10086106	0.98133699	5.19786894	2.02E-07	5.75E-05	4.24033216	NA	NA
evm.TU.Scaffold_2593_HRSCAF-2754.819	45.36005648	5.11554058	0.87289926	5.86040201	4.62E-09	3.56E-06	5.44855	phosphatidylinositol transfer protein beta isoform-like [Orbicella faveolata]	NA
evm.TU.Scaffold_40161_HRSCAF-40537.153	9.148253935	5.13642781	1.66463455	3.08561889	0.00203129	0.06138682	1.21192485	NA	NA
evm.TU.Scaffold_73_HRSCAF-87.287	23284.46598	5.2485568	1.6795325	3.12501055	0.00177799	0.05548727	1.25580667	Transposon Ty3-I Gag-Pol polyprotein [Exaiptasia pallida]	NA
evm.TU.Scaffold_40196_HRSCAF-40652.855	4.7313815	5.25558264	1.714672	3.06506588	0.00217622	0.06445699	1.19072997	NA	NA
evm.TU.Scaffold_40138_HRSCAF-40465.1823	13.93354295	5.42581471	1.89392992	2.8648445	0.00417214	0.09463215	1.02396131	PREDICTED- caskin-1-like, partial [Hydra vulgaris]	NA
evm.TU.Scaffold_743_HRSCAF-861.1808	54.25002582	5.42821183	1.50655907	3.60305276	0.0003145	0.01834329	1.73652284	hypothetical protein AC249_AIPGENE5294 [Exaiptasia pallida]	NA
evm.TU.Scaffold_40152_HRSCAF-40518.1116	30204.87894	5.43372329	1.67537192	3.24329375	0.00118156	0.04331428	1.36336893	NA	NA
evm.TU.Scaffold_40164_HRSCAF-40545.997	81.76946288	5.62218953	1.70876626	3.29020398	0.00100115	0.03881918	1.41095364	transcription factor Sox-17-beta.1-like [Stylophora pistillata]	K09271
evm.TU.Scaffold_11_HRSCAF-12.1301	67.61760119	5.67617664	1.10807597	5.12255188	3.01E-07	8.15E-05	4.08884239	predicted protein [Nematostella vectensis]	NA
evm.TU.Scaffold_40168_HRSCAF-40561.347	4.511871285	5.85077212	1.91892282	3.04898773	0.00229614	0.06612752	1.17961775	NA	NA
evm.TU.Scaffold_40196_HRSCAF-40652.49	18.2002734	5.9492346	1.39825792	4.25474765	2.09E-05	0.00233034	2.63258127	[Strongylocentrotus purpuratus]	NA
evm.TU.Scaffold_40196_HRSCAF-40652.128	9.590641506	5.99452378	1.68833862	3.55054592	0.00038443	0.02117256	1.67422669	craniofacial development protein 2-like [Centruroides sculpturatus]	NA
evm.TU.Scaffold_40196_HRSCAF-40652.53	5.760044483	6.02512489	2.11185282	2.85300416	0.00433081	0.09700864	1.01318959	hypothetical protein CAPTEDRAFT_106103, partial [Capitella teleta]	NA
evm.TU.Scaffold_40109_HRSCAF-40345.774	6.155240907	6.13198587	1.99432345	3.07471984	0.002107	0.06267433	1.20291029	E3 ubiquitin-protein ligase MIB1 isoform X5 [Anas platyrhynchos]	K16060
evm.TU.Scaffold_743_HRSCAF-861.179	12.43979844	6.17282798	1.7751666	3.4773232	0.00050645	0.02452381	1.61041206	NA	NA
evm.TU.Scaffold_40164_HRSCAF-40545.690	111.3444021	6.23439813	1.18615392	5.25597733	1.47E-07	4.45E-05	4.35163999	transmembrane 9 superfamily member 1 isoform X3 [Dasypus novemcinctus]	NA

evm.TU.Scaffold_11_HRSCAF-12.1687	16.53414513	6.30115367	1.67681044	3.75782112	0.0001714	0.01193604	1.92313963	PREDICTED- cathepsin Z-like [Hydra vulgaris]	K08568
evm.TU.Scaffold_2593_HRSCAF-2754.249	9.593266076	6.46525603	1.92822047	3.35296515	0.00079951	0.03296505	1.48194627	NA	NA
evm.TU.Scaffold_117_HRSCAF-135.563	7.758522682	6.47866765	2.25143273	2.87757549	0.00400744	0.09271435	1.03285303	biogenic amine-like GPCR [Tripedalia cystophora]	NA
evm.TU.Scaffold_40152_HRSCAF-40518.413	6.32291009	6.55632742	2.03553021	3.22094332	0.00127769	0.04532223	1.34368875	hypothetical protein BSL78_26493 [Apostichopus japonicus]	NA
evm.TU.Scaffold_40122_HRSCAF-40411.1601	13.24808279	6.57070169	1.57138006	4.1814847	2.90E-05	0.00307515	2.51213398	hypothetical protein EGW08_016106 [Elysia chlorotica]	NA
evm.TU.Scaffold_40135_HRSCAF-40458.585	2960.878351	6.57313842	1.57787322	4.16582165	3.10E-05	0.00324607	2.48864239	hypothetical protein pdam_00021684 [Pocillopora damicornis]	NA
evm.TU.Scaffold_40109_HRSCAF-40345.442	37.555648	6.59036999	1.33433527	4.93906603	7.85E-07	0.0001651	3.78225556	NA	NA
novel_gene_559_5d572bfb	15.74566424	6.61658789	2.05998435	3.21196027	0.00131833	0.04644325	1.33307744	hypothetical protein BSL78_12747 [Apostichopus japonicus]	NA
evm.TU.Scaffold_168_HRSCAF-201.63	9.816324464	6.72833254	2.21446778	3.03835197	0.00237876	0.06775722	1.16904442	Zinc finger MYM-type protein 1 [Intoshia linei]	NA
evm.TU.Scaffold_40122_HRSCAF-40411.100	15.73520494	6.73769923	1.94679425	3.46092004	0.00053833	0.0255917	1.59190092	predicted protein [Nematostella vectensis]	NA
evm.TU.Scaffold_40135_HRSCAF-40458.320	6.949535141	6.81519941	1.89267714	3.60082513	0.00031721	0.01844951	1.73401523	R2DM [Exaiptasia pallida]	NA
novel_gene_228_5d572bfb	8.206535876	6.86500538	2.10553824	3.26045154	0.00111235	0.0416571	1.38031099	NA	#N/A
evm.TU.Scaffold_40122_HRSCAF-40411.1449	45.97961574	6.95330331	1.39618302	4.98022337	6.35E-07	0.0001392	3.85635141	PREDICTED- uncharacterized protein LOC100201425 [Hydra vulgaris]	NA
evm.TU.Scaffold_40164_HRSCAF-40545.151	7.412352832	6.97578656	2.17549229	3.20653242	0.00134345	0.04701404	1.32777247	NA	NA
evm.TU.Scaffold_40114_HRSCAF-40359.514	9.572707612	7.02202106	2.07885738	3.37782723	0.00073061	0.03090701	1.50994295	NA	NA
evm.TU.Scaffold_2593_HRSCAF-2754.1190	10.80556487	7.45019186	1.94811262	3.82431272	0.00013114	0.0098221	2.00779587	uncharacterized protein LOC114530181 [Dendronephthya gigantea]	NA
evm.TU.Scaffold_40122_HRSCAF-40411.1211	12.02588782	7.56652042	1.74343823	4.33999913	1.42E-05	0.00173496	2.76070978	PREDICTED- alpha-crystallin B chain-like [Hydra vulgaris]	K09542
evm.TU.Scaffold_40138_HRSCAF-40465.1849	12.93603653	7.79030523	1.95566028	3.98346549	6.79E-05	0.00584372	2.23331097	caudatus]	NA
evm.TU.Scaffold_11_HRSCAF-12.1312	542.7693532	8.17460734	2.30218351	3.55080614	0.00038405	0.02117256	1.67422669	uncharacterized protein LOC111334028 [Stylophora pistillata]	NA
evm.TU.Scaffold_743_HRSCAF-861.64	182.2429487	8.470752	2.62460332	3.22744086	0.00124903	0.04484013	1.34833319	NA	NA
evm.TU.Scaffold_40168_HRSCAF-40561.227	31.73284768	8.55771016	1.6963492	5.04478097	4.54E-07	0.00010985	3.95919203	uncharacterized protein LOC110045899 [Orbicella faveolata]	NA
evm.TU.Scaffold_40170_HRSCAF-40571.1128	1018.325761	9.05615621	2.76694953	3.27297484	0.00106422	0.04028942	1.39480904	Uromodulin [Stylophora pistillata]	K19899
evm.TU.Scaffold_11_HRSCAF-12.40	1525.171906	11.860918	3.47383	3.4143634	0.00063931	0.02856602	1.54415033	[Strongylocentrotus purpuratus]	NA
evm.TU.Scaffold_40161_HRSCAF-40537.701	1231.351033	13.6906785	2.63863823	5.18853945	2.12E-07	5.88E-05	4.23062267	PREDICTED- hemicentin-2-like [Acropora digitifera]	K06753
evm.TU.Scaffold_40168_HRSCAF-40561.967	12890.13433	15.1389196	3.57063567	4.23983879	2.24E-05	0.00247738	2.60600755	hypothetical protein AC249_AIPGENE22499 [Exaiptasia pallida]	NA
evm.TU.Scaffold_40161_HRSCAF-40537.258	37.4973811	20.746274	3.90848899	5.3080037	1.11E-07	3.78E-05	4.4225082	NA	NA
evm.TU.Scaffold_40141_HRSCAF-40491.114	4915.88111	28.5280794	3.906748	7.30225737	2.83E-13	7.18E-10	9.14387556	NA	NA

Table S4. . KEGG enrichment of genes of animals in the late day (ZT11) that had either experienced control conditions or had been exposed to MSD for two consecutive nights

Cluster	ID	Description	GeneRatio	BgRatio	pvalue	p.adjust	qvalue	geneID	Count	
1	Up	map00565	Ether lipid metabolism	27851	31/14152	2.16E-05	0.003723336	0.003121769	K00994/K13510/K01019/K01080	4
2	Up	map00600	Sphingolipid metabolism	27851	52/14152	0.000171024	0.014708059	0.012331727	K04708/K01019/K12382/K01080	4
3	Up	map04977	Vitamin digestion and absorption	27820	25/14152	0.00031442	0.018026724	0.015114206	K14073/K14616/K14613	3
4	Up	map04975	Fat digestion and absorption	27820	28/14152	0.000442689	0.019035608	0.015960087	K14073/K01080/K00626	3
5	Up	map04820	Cytoskeleton in muscle cells	27881	155/14152	0.00146046	0.050239836	0.042122751	K06255/K19720/K09377/K04659/K05738	5
6	Down	map05222	Small cell lung cancer	33117	74/14152	8.91E-10	2.20E-07	1.15E-07	K05635/K06245/K02187/K05637/K04377/K02159/K02580/K04456/K02649	9
7	Down	map05165	Human papillomavirus infection	33178	231/14152	2.32E-07	2.86E-05	1.50E-05	K05635/K06245/K02187/K05637/K02159/K09444/K02580/K04659/K04456/K02649/K12076	11
8	Down	map05417	Lipid and atherosclerosis	33117	160/14152	7.78E-07	5.29E-05	2.78E-05	K02187/K10645/K02159/K02580/K04353/K04456/K02649/K12800/K21421	9
9	Down	map04625	C-type lectin receptor signaling path	33055	82/14152	8.57E-07	5.29E-05	2.78E-05	K07293/K09444/K02580/K07369/K04456/K02649/K12800	7
10	Down	map04722	Neurotrophin signaling pathway	33055	90/14152	1.62E-06	7.99E-05	4.19E-05	K07293/K04464/K02159/K02580/K04353/K04456/K02649	7
11	Down	map04662	B cell receptor signaling pathway	33025	60/14152	2.12E-06	8.74E-05	4.58E-05	K12230/K02580/K07369/K04456/K02649/K15909	6
12	Down	map05220	Chronic myeloid leukemia	33025	63/14152	2.84E-06	0.000100165	5.25E-05	K07293/K04377/K02159/K02580/K04456/K02649	6
13	Down	map04510	Focal adhesion	33086	152/14152	5.42E-06	0.000167379	8.77E-05	K05635/K06245/K05637/K04353/K04659/K04456/K02649/K04437	8
14	Down	map04933	AGE-RAGE signaling pathway in diab	33025	76/14152	8.55E-06	0.000234626	0.000122987	K02187/K02159/K02580/K04456/K02649/K21421	6
15	Down	map05146	Amoebiasis	33025	87/14152	1.87E-05	0.000461233	0.000241771	K05635/K06245/K02187/K05637/K02580/K02649	6
16	Down	map04660	T cell receptor signaling pathway	33025	92/14152	2.57E-05	0.00057723	0.000302575	K07293/K02580/K07369/K04456/K02649/K12076	6
17	Down	map05145	Toxoplasmosis	33025	95/14152	3.09E-05	0.000635161	0.000332942	K05635/K06245/K02187/K05637/K02580/K04456	6
18	Down	map05131	Shigellosis	33086	205/14152	4.74E-05	0.00089473	0.000469004	K11974/K02159/K02580/K17988/K07369/K04456/K02649/K12800	8
19	Down	map04151	PI3K-Akt signaling pathway	33117	268/14152	5.07E-05	0.00089473	0.000469004	K05635/K06245/K05637/K04377/K12230/K02580/K04659/K04456/K02649	9
20	Down	map04512	ECM-receptor interaction	32994	66/14152	6.12E-05	0.001008127	0.000528445	K06265/K05635/K06245/K05637/K04659	5
21	Down	map04210	Apoptosis	33025	110/14152	7.05E-05	0.00102866	0.000539208	K02187/K02159/K12641/K02580/K04456/K02649	6
22	Down	map05132	Salmonella infection	33086	217/14152	7.08E-05	0.00102866	0.000539208	K02187/K04377/K02159/K02580/K17637/K04456/K12800/K04437	8
23	Down	map05160	Hepatitis C	33025	112/14152	7.79E-05	0.001029848	0.000539831	K02187/K04377/K02159/K02580/K04456/K02649	6
24	Down	map05170	Human immunodeficiency virus 1 inf	33055	163/14152	7.92E-05	0.001029848	0.000539831	K02187/K05743/K02159/K22544/K02580/K04456/K02649	7
25	Down	map05210	Colorectal cancer	32994	72/14152	9.30E-05	0.00114881	0.000602189	K02187/K04377/K02159/K04456/K02649	5
26	Down	map04931	Insulin resistance	32994	74/14152	0.000106034	0.001247159	0.000653742	K07293/K05695/K02580/K04456/K02649	5
27	Down	map04360	Axon guidance	33025	128/14152	0.000162873	0.00174912	0.000916862	K07293/K05110/K05108/K05463/K05743/K02649	6
28	Down	map05164	Influenza A	33025	128/14152	0.000162873	0.00174912	0.000916862	K02187/K02159/K02580/K04456/K02649/K12800	6
29	Down	map05152	Tuberculosis	33025	131/14152	0.000184847	0.001830369	0.000959452	K02187/K02159/K02580/K07369/K04456/K05398	6
30	Down	map05213	Endometrial cancer	32964	45/14152	0.000186714	0.001830369	0.000959452	K04377/K02159/K04456/K02649	4
31	Down	map05161	Hepatitis B	33025	132/14152	0.00019267	0.001830369	0.000959452	K02187/K04377/K02159/K02580/K04456/K02649	6
32	Down	map01524	Platinum drug resistance	32964	46/14152	0.000203508	0.001861722	0.000975887	K02187/K02159/K04456/K02649	4
33	Down	map00830	Retinol metabolism	32964	50/14152	0.000281697	0.002484972	0.001302585	K00515/K15734/K00699/K11150	4
34	Down	map05200	Pathways in cancer	33147	417/14152	0.000309384	0.002635097	0.001381278	K21283/K05635/K06245/K02187/K05637/K04377/K02159/K02580/K04456/K02649	10
35	Down	map04350	TGF-beta signaling pathway	32994	96/14152	0.000360564	0.002919787	0.001530508	K21283/K04377/K04661/K23318/K22687	5
36	Down	map05168	Herpes simplex virus 1 infection	33025	149/14152	0.00037057	0.002919787	0.001530508	K07293/K02187/K02159/K02580/K04456/K02649	6
37	Down	map04668	TNF signaling pathway	32994	97/14152	0.000378272	0.002919787	0.001530508	K02187/K09444/K02580/K04456/K02649	5
38	Down	map05211	Renal cell carcinoma	32964	56/14152	0.000436404	0.003266418	0.001712207	K07293/K04353/K04456/K02649	4
39	Down	map04917	Prolactin signaling pathway	32964	57/14152	0.000467076	0.003296221	0.001727829	K09444/K02580/K04456/K02649	4
40	Down	map05221	Acute myeloid leukemia	32964	57/14152	0.000467076	0.003296221	0.001727829	K04377/K02580/K04456/K02649	4
41	Down	map04361	Axon regeneration	32964	59/14152	0.00053298	0.003656838	0.00191686	K05110/K09442/K11593/K06704	4
42	Down	map05231	Choline metabolism in cancer	32964	61/14152	0.000605245	0.004040419	0.002117927	K00994/K15377/K04456/K02649	4
43	Down	map05205	Proteoglycans in cancer	33025	167/14152	0.000678142	0.004333301	0.002271451	K07293/K02187/K04377/K04456/K02649/K04437	6
44	Down	map05212	Pancreatic cancer	32964	63/14152	0.000684205	0.004333301	0.002271451	K02159/K02580/K04456/K02649	4
45	Down	map05167	Kaposi sarcoma-associated herpesv	33025	169/14152	0.000721808	0.004375527	0.002293585	K02187/K04377/K02159/K02580/K04456/K02649	6
46	Down	map04666	Fc gamma R-mediated phagocytosis	32964	64/14152	0.000726302	0.004375527	0.002293585	K05743/K04456/K02649/K15909	4
47	Down	map05169	Epstein-Barr virus infection	33025	172/14152	0.000791356	0.004653929	0.002439519	K02187/K04377/K02159/K02580/K04456/K02649	6
48	Down	map05162	Measles	32994	117/14152	0.000890358	0.004903057	0.002570109	K02187/K02159/K02580/K04456/K02649	5
49	Down	map04010	MAPK signaling pathway	33055	243/14152	0.000899031	0.004903057	0.002570109	K02187/K04377/K04464/K02580/K04353/K04456/K04437	7
50	Down	map04211	Longevity regulating pathway	32964	68/14152	0.00091312	0.004903057	0.002570109	K02159/K02580/K04456/K02649	4
51	Down	map04623	Cytosolic DNA-sensing pathway	32964	68/14152	0.00091312	0.004903057	0.002570109	K02187/K22544/K02580/K12800	4
52	Down	map04014	Ras signaling pathway	33025	182/14152	0.001061225	0.005529274	0.002898362	K07293/K02580/K04353/K17637/K04456/K02649	6
53	Down	map05135	Yersinia infection	32994	122/14152	0.001074515	0.005529274	0.002898362	K05743/K02580/K04456/K02649/K12800	5
54	Down	map05235	PD-L1 expression and PD-1 checkpo	32964	73/14152	0.001191299	0.00600512	0.003147793	K07293/K02580/K04456/K02649	4
55	Down	map04932	Non-alcoholic fatty liver disease	32994	127/14152	0.001285557	0.006273226	0.00328833	K02187/K02159/K02580/K04456/K02649	5
56	Down	map04670	Leukocyte transendothelial migratic	32964	75/14152	0.001317555	0.006273226	0.00328833	K07293/K04353/K02649/K21421	4
57	Down	map05166	Human T-cell leukemia virus 1 infect	33025	191/14152	0.001360069	0.006273226	0.00328833	K04377/K02159/K02580/K04456/K02649/K12076	6
58	Down	map05207	Chemical carcinogenesis - receptor	32994	129/14152	0.001378018	0.006273226	0.00328833	K04377/K02580/K04456/K02649/K00699	5
59	Down	map04066	HIF-1 signaling pathway	32964	76/14152	0.001384063	0.006273226	0.00328833	K02580/K04456/K02649/K21421	4
60	Down	map05163	Human cytomegalovirus infection	33025	192/14152	0.001396872	0.006273226	0.00328833	K02187/K04377/K02159/K02580/K04456/K02649	6
61	Down	map04148	Efferocytosis	32994	131/14152	0.001475302	0.006507135	0.003410942	K14802/K07293/K02187/K17253/K06704	5
62	Down	map05133	Pertussis	32964	79/14152	0.001597619	0.006923016	0.003628941	K02187/K09444/K02580/K12800	4
63	Down	map04620	Toll-like receptor signaling pathway	32964	80/14152	0.001673621	0.007127318	0.003736033	K02580/K04456/K05398/K02649	4
64	Down	map04071	Sphingolipid signaling pathway	32964	83/14152	0.001916667	0.008024012	0.004206066	K02159/K02580/K04456/K02649	4
65	Down	map04910	Insulin signaling pathway	32964	88/14152	0.002374365	0.009774469	0.005123629	K05695/K04456/K02649/K15909	4
66	Down	map04613	Neutrophil extracellular trap format	32964	90/14152	0.002576822	0.010434019	0.005469356	K02580/K04456/K02649/K21421	4
67	Down	map05120	Epithelial cell signaling in Helicobac	32964	91/14152	0.002682366	0.010686202	0.005601546	K07293/K02187/K02580/K06704	4
68	Down	map05130	Pathogenic Escherichia coli infectio	32994	152/14152	0.002826471	0.01108156	0.005808787	K07293/K02187/K02159/K02580/K12800	5
69	Down	map04217	Necroptosis	32964	96/14152	0.003254842	0.012520397	0.006563004	K11974/K02159/K12800/K21421	4
70	Down	map05416	Viral myocarditis	32933	47/14152	0.003294841	0.012520397	0.006563004	K06265/K02187/K05637	3
71	Down	map05418	Fluid shear stress and atherosclero:	32964	99/14152	0.003635554	0.013605784	0.007131947	K04464/K02580/K04456/K02649	4
72	Down	map05230	Central carbon metabolism in cance	32933	49/14152	0.003709654	0.013675887	0.007168694	K04377/K04456/K02649	3
73	Down	map05218	Melanoma	32933	50/14152	0.003928459	0.014269551	0.007479884	K02159/K04456/K02649	3
74	Down	map04936	Alcoholic liver disease	32964	105/14152	0.004485596	0.015827746	0.008296667	K02187/K00232/K02580/K04456	4
75	Down	map05224	Breast cancer	32964	105/14152	0.004485596	0.015827746	0.008296667	K04377/K02159/K04456/K02649	4
76	Down	map04391	Hippo signaling pathway - fly	32933	53/14152	0.00463146	0.016110093	0.008444668	K16669/K04377/K12076	3
77	Down	map04024	cAMP signaling pathway	32994	172/14152	0.004787711	0.016110093	0.008444668	K00232/K02580/K04353/K04456/K02649	5
78	Down	map04920	Adipocytokine signaling pathway	32933	54/14152	0.00488158	0.016110093	0.008444668	K07293/K02580/K04456	3
79	Down	map05214	Glioma	32933	54/14152	0.00488158	0.016110093	0.008444668	K02159/K04456/K02649	3
80	Down	map04072	Phospholipase D signaling pathway	32964	108/14152	0.004956952	0.016110093	0.008444668	K07293/K04456/K02649/K04606	4
81	Down	map05226	Gastric cancer	32964	108/14152	0.004956952	0.016110093	0.008444668	K04377/K02159/K04456/K02649	4
82	Down	map05203	Viral carcinogenesis	32994	174/14152	0.005026205	0.016123021	0.008451445	K02187/K02159/K02580/K02649/K12076	5
83	Down	map04550	Signaling pathways regulating plurip	32964	114/14152	0.00599722	0.018991196	0.009954899	K04377/K04456/K02649/K22687	4
84	Down	map05225	Hepatocellular carcinoma	32964	116/14152	0.006373906	0.019928541	0.010446241	K04377/K02159/K04456/K02649	4
85	Down	map05223	Non-small cell lung cancer	32933	60/14152	0.006553056	0.020028388	0.010498579	K02159/K04456/K02649	3
86	Down	map04218	Cellular senescence	32964	117/14152	0.006568014	0.020028388	0.010498579	K04377/K02580/K04456/K02649	4
87	Down	map04973	Carbohydrate digestion and absorpt	32905	22/14152	0.008505782	0.025621076	0.013430183	K04456/K02649	2
88	Down	map01521	EGFR tyrosine kinase inhibitor resist	32933	67/14152	0.008886198	0.026320993	0.013797068	K02159/K04456/K02649	3
89	Down	map05022	Pathways of neurodegeneration - mu	33055	368/14152	0.008951269	0.026320993	0.013797068	K02187/K02159/K08844/K02580/K04533/K04575/K21421	7
90	Down	map04012	ErbB signaling pathway	32933	69/14152	0.009630983	0.027986504	0.014670104	K04377/K04456/K02649	3
91	Down	map05020	Prion disease	32994	209/14152	0.010681136	0.030677215	0.016080535	K05635/K02187/K02159/K02649/K21421	5
92	Down	map04977	Vitamin digestion and absorption	32905	25/14152	0.010910995	0.030977194	0.016237779	K14616/K14592	2
93	Down	map01523	Antifolate resistance	32905	26/14152	0.011771772	0.033041222	0.017319712	K02580/K01307	2
94	Down	map05010	Alzheimer disease	33025	300/14152	0.012153767	0.033730117	0.01768082	K02187/K02580/K04456/K02649/K06704/K21421	6

95	Down	map05412	Arrhythmogenic right ventricular car	32933	76/14152	0.01251846	0.034356217	0.018009012	K06265/K05637/K12641	3
96	Down	map01522	Endocrine resistance	32933	77/14152	0.012967064	0.035087068	0.018392113	K02159/K04456/K02649	3
97	Down	map04630	JAK-STAT signaling pathway	32964	143/14152	0.013068868	0.035087068	0.018392113	K07293/K04377/K04456/K02649	4
98	Down	map04621	NOD-like receptor signaling pathwa	32964	145/14152	0.01369255	0.036366234	0.019062633	K11974/K02580/K12800/K21421	4
99	Down	map04657	IL-17 signaling pathway	32933	80/14152	0.014367673	0.037753355	0.019789741	K02187/K04464/K02580	3
100	Down	map05215	Prostate cancer	32933	81/14152	0.014852888	0.038617508	0.020242717	K02580/K04456/K02649	3
101	Down	map04520	Adherens junction	32933	83/14152	0.015850959	0.040783197	0.021377938	K05705/K05695/K04353	3
102	Down	map05142	Chagas disease	32933	85/14152	0.016886006	0.042998388	0.022539108	K02580/K04456/K02649	3
103	Down	map04820	Cytoskeleton in muscle cells	32964	155/14152	0.017094468	0.043085036	0.022584528	K06265/K05637/K12641/K04659	4
104	Down	map00983	Drug metabolism - other enzymes	32905	32/14152	0.017528731	0.043733298	0.022924337	K00699/K13421	2
105	Down	map05410	Hypertrophic cardiomyopathy	32933	87/14152	0.017958152	0.044356635	0.023251081	K06265/K05637/K12641	3
106	Down	map05415	Diabetic cardiomyopathy	32964	160/14152	0.018976942	0.046408958	0.024326878	K02580/K04456/K02649/K21421	4
107	Down	map04611	Platelet activation	32933	90/14152	0.019636137	0.046940802	0.024605662	K04353/K04456/K02649	3
108	Down	map05216	Thyroid cancer	32905	34/14152	0.019664161	0.046940802	0.024605662	K04377/K02159	2
109	Down	map04062	Chemokine signaling pathway	32964	162/14152	0.019764548	0.046940802	0.024605662	K02580/K04353/K04456/K02649	4
110	Down	map05206	MicroRNAs in cancer	32994	245/14152	0.019960259	0.046954134	0.024612651	K02187/K04377/K04464/K02580/K02649	5
111	Down	map04919	Thyroid hormone signaling pathway	32933	91/14152	0.020214107	0.047102682	0.024690518	K04377/K04456/K02649	3
112	Down	map04650	Natural killer cell mediated cytotoxi	32933	94/14152	0.022004022	0.050794331	0.026625624	K07293/K02187/K02649	3
113	Down	map05414	Dilated cardiomyopathy	32933	95/14152	0.022619341	0.05173127	0.027116754	K06265/K05637/K12641	3
114	Down	map04926	Relaxin signaling pathway	32933	96/14152	0.023244002	0.052672188	0.027609969	K02580/K04456/K02649	3
115	Down	map05208	Chemical carcinogenesis - reactive	32964	175/14152	0.025376254	0.056122168	0.029418396	K07293/K02580/K04456/K02649	4
116	Down	map04370	VEGF signaling pathway	32905	39/14152	0.025448108	0.056122168	0.029418396	K04456/K02649	2
117	Down	map05030	Cocaine addiction	32905	39/14152	0.025448108	0.056122168	0.029418396	K02580/K04606	2
118	Down	map04215	Apoptosis - multiple species	32905	40/14152	0.026678382	0.058314694	0.030567685	K02187/K02159	2
119	Down	map04068	FoxO signaling pathway	32933	103/14152	0.02787803	0.060402399	0.031662029	K10305/K04456/K02649	3
120	Down	map04380	Osteoclast differentiation	32933	104/14152	0.028577314	0.061379101	0.032174001	K02580/K04456/K02649	3
121	Down	map01240	Biosynthesis of cofactors	33025	375/14152	0.032223309	0.068613425	0.035966125	K00515/K15734/K01307/K00699/K13421/K00966	6
122	Down	map04664	Fc epsilon RI signaling pathway	32905	47/14152	0.035934238	0.075218279	0.03942829	K04456/K02649	2
123	Down	map04923	Regulation of lipolysis in adipocytes	32905	47/14152	0.035934238	0.075218279	0.03942829	K04456/K02649	2
124	Down	map04390	Hippo signaling pathway	32933	115/14152	0.036880081	0.076549412	0.04012605	K21283/K04377/K12076	3
125	Down	map05217	Basal cell carcinoma	32905	48/14152	0.037344489	0.076867406	0.040292738	K21283/K02159	2
126	Down	map04929	GnRH secretion	32905	51/14152	0.041700087	0.085123317	0.044620362	K04456/K02649	2
127	Down	map00513	Various types of N-glycan biosynthes	32905	55/14152	0.047787193	0.095189006	0.049896645	K00738/K00753	2
128	Down	map04070	Phosphatidylinositol signaling syster	32905	55/14152	0.047787193	0.095189006	0.049896645	K02649/K15909	2
129	Down	map04721	Synaptic vesicle cycle	32905	55/14152	0.047787193	0.095189006	0.049896645	K15015/K15293	2

Table S5. Meta-analysis using Combat-Seq to analyze Round 1 and Round 2, which included a second set of LSD animals.

	baseMean	log2FoldChange	lfcSE	stat	pvalue	padj		sdVbase	light	mech	metaSeq		names	
evm.TU.Scaffold_168_HRSCAF-201.174	17.26891738	3.805986835	0.678041415	5.613207029	1.99E-08	0.000398453	neuronal acetylcholine receptor subunit alpha-10-like [Stylophora pistillata]	7.20E-06	#N/A	0.078670639	0.018099815	evm.TU.Scaffold_168_HRSCAF-201.174	neuronal acetylcholine receptor subunit alpha-10-like [Stylophora pistillata]	ChrnaIE
evm.TU.Scaffold_40196_HRSCAF-40652.1137	427.5041636	3.572094214	0.680315078	5.250646843	1.52E-07	0.001520359	steroid 17-alpha-hydroxylase/17,20 lyase-like isoform X4 [Acropora millepora]	0.009776756	0.005401449	0.096144576	0.006965429	evm.TU.Scaffold_40196_HRSCAF-40652.1137	steroid 17-alpha-hydroxylase/17,20 lyase-like isoform X4 [Acropora millepora]	CYP17A1
evm.TU.Scaffold_40109_HRSCAF-40345.521	156.2302445	1.939642683	0.402734054	4.816187422	1.46E-06	0.005871228	uncharacterized protein LOC113671488 [Pocillopora damicornis]	#N/A	#N/A	0.00189248	#N/A	evm.TU.Scaffold_40109_HRSCAF-40345.521	uncharacterized protein LOC113671488 [Pocillopora damicornis]	NA
evm.TU.Scaffold_743_HRSCAF-861.617	70.73913706	3.107494419	0.643396869	4.829825213	1.37E-06	0.005871228	uncharacterized protein LOC114949530 [Acropora millepora]	#N/A	#N/A	#N/A	0.012312001	evm.TU.Scaffold_743_HRSCAF-861.617	uncharacterized protein LOC114949530 [Acropora millepora]	NA
evm.TU.Scaffold_11_HRSCAF-12.791	338.0125023	1.763214511	0.359340221	4.90681089	9.26E-07	0.005871228	uncharacterized protein LOC114973330 [Acropora millepora]	0.009776756	#N/A	#N/A	0.020494314	evm.TU.Scaffold_11_HRSCAF-12.791	uncharacterized protein LOC114973330 [Acropora millepora]	nA
evm.TU.Scaffold_40161_HRSCAF-40537.1182	306.7018107	1.661802379	0.351839922	4.723177434	2.32E-06	0.007763596	PREDICTED- uncharacterized protein LOC107351686 [Acropora digitifera]	0.020006595	#N/A	#N/A	0.027947119	evm.TU.Scaffold_40161_HRSCAF-40537.1182	PREDICTED- uncharacterized protein LOC107351686 [Acropora digitifera]	NA
evm.TU.Scaffold_168_HRSCAF-201.1383	2477.217225	1.808387983	0.415182422	4.355646785	1.33E-05	0.038024533	uncharacterized protein LOC111346558 [Stylophora pistillata]	#N/A	#N/A	#N/A	0.011650921	evm.TU.Scaffold_168_HRSCAF-201.1383	uncharacterized protein LOC111346558 [Stylophora pistillata]	NA
evm.TU.Scaffold_743_HRSCAF-861.1808	48.47716531	3.134780947	0.734515379	4.267822072	1.97E-05	0.047414209	hypothetical protein AC249_AIPGENE5294 [Exaiptasia pallida]	#N/A	#N/A	0.018343287	0.028793307	evm.TU.Scaffold_743_HRSCAF-861.1808	hypothetical protein AC249_AIPGENE5294 [Exaiptasia pallida]	Hypothetical
novel_gene_566_5d572bfb	12.3229496	2.470012134	0.581197467	4.249867343	2.14E-05	0.047414209	hypothetical protein CRYPA_887 [uncultured SUP05 cluster bacterium]	#N/A	#N/A	0.022546612	#N/A	novel_gene_566_5d572bfb	hypothetical protein CRYPA_887 [uncultured SUP05 cluster bacterium]	
evm.TU.Scaffold_40196_HRSCAF-40652.1138	872.9847023	2.303878198	0.544978592	4.227465503	2.36E-05	0.047414209	steroid 17-alpha-hydroxylase/17,20 lyase-like isoform X6 [Acropora millepora]	0.029630207	#N/A	#N/A	0.009440169	evm.TU.Scaffold_40196_HRSCAF-40652.1138	steroid 17-alpha-hydroxylase/17,20 lyase-like isoform X6 [Acropora millepora]	CYP17A1
evm.TU.Scaffold_168_HRSCAF-201.1168	181.7012366	1.279704184	0.306428749	4.176188386	2.96E-05	0.054064236	dopamine receptor 2-like [Orbicella faveolata]	#N/A	#N/A	#N/A	#N/A	evm.TU.Scaffold_168_HRSCAF-201.1168	dopamine receptor 2-like [Orbicella faveolata]	DRD2
evm.TU.Scaffold_11_HRSCAF-12.1766	10.95996823	2.797370787	0.680166255	4.112775027	3.91E-05	0.0653572	craniofacial development protein 2-like isoform X2 [Centruroides sculpturatus]	#N/A	#N/A	0.01929046	#N/A	evm.TU.Scaffold_11_HRSCAF-12.1766	craniofacial development protein 2-like isoform X2 [Centruroides sculpturatus]	CFDP2-like
evm.TU.Scaffold_40170_HRSCAF-40571.589	496.7209893	2.265314836	0.560205012	4.043724687	5.26E-05	0.070362371	hypothetical protein B5V51_7063 [Heliothis virescens]	#N/A	#N/A	#N/A	0.039322341	evm.TU.Scaffold_40170_HRSCAF-40571.589	phospholipase A1-like isoform X4 [Rhopilema esculentum]	PLA-like
evm.TU.Scaffold_40161_HRSCAF-40537.258	43.53277635	6.537320576	1.615563661	4.046464236	5.20E-05	0.070362371	NA	#N/A	0.0000802	0.0000378	0.044065496	evm.TU.Scaffold_40161_HRSCAF-40537.258	NA	NA
evm.TU.Scaffold_743_HRSCAF-861.616	84.30799704	5.400045578	1.335349235	4.043920075	5.26E-05	0.070362371	uncharacterized protein LOC111324678 [Stylophora pistillata]	#N/A	#N/A	#N/A	0.009329947	evm.TU.Scaffold_743_HRSCAF-861.616	uncharacterized protein LOC111324678 [Stylophora pistillata]	NA
evm.TU.Scaffold_117_HRSCAF-135.264	184.0863374	1.001399732	0.252665272	3.963345352	7.39E-05	0.092669822	PREDICTED- protein SYS1 homolog [Hydra vulgaris]	#N/A	#N/A	#N/A	#N/A	evm.TU.Scaffold_117_HRSCAF-135.264	protein SYS1 homolog [Rhopilema esculentum]	SYS1
evm.TU.Scaffold_2593_HRSCAF-2754.1129	42.24922034	6.208849203	1.586645402	3.913192698	9.11E-05	0.098507879	NA	#N/A	#N/A	#N/A	#N/A	evm.TU.Scaffold_2593_HRSCAF-2754.1129	E3 SUMO-protein ligase ZBED1-like [Rhopilema esculentum]	ZBED1
evm.TU.Scaffold_11_HRSCAF-12.320	217.7097235	2.027778743	0.521685646	3.886974387	0.000101501	0.098507879	PREDICTED- choline/ethanolaminephosphotransferase 1 isoform X5 [Nasonia vitri	#N/A	0.018407876	0.006605745	0.018099815	evm.TU.Scaffold_11_HRSCAF-12.320	cholinephosphotransferase 1-like isoform X2 [Rhopilema esculentum]	CHPT1-like
evm.TU.Scaffold_2593_HRSCAF-2754.964	170.7361897	1.557970406	0.399889236	3.896004857	9.78E-05	0.098507879	PREDICTED- uncharacterized protein LOC101240914 [Hydra vulgaris]	#N/A	#N/A	#N/A	0.044065496	evm.TU.Scaffold_2593_HRSCAF-2754.964	uncharacterized protein	
evm.TU.Scaffold_40109_HRSCAF-40345.786	2834.682115	1.495188754	0.383361649	3.90020431	9.61E-05	0.098507879	transmembrane protease serine 9-like [Stylophora pistillata]	#N/A	#N/A	0.028566016	0.010833189	evm.TU.Scaffold_40109_HRSCAF-40345.786	serine protease hepsin-like [Rhopilema esculentum]	HPN
evm.TU.Scaffold_73_HRSCAF-87.412	10.5376805	5.000154979	1.287655682	3.883145977	0.000103114	0.098507879	uncharacterized protein LOC113667399 [Pocillopora damicornis]	#N/A	#N/A	#N/A	#N/A	evm.TU.Scaffold_73_HRSCAF-87.412	uncharacterized protein LOC113667399 [Pocillopora damicornis]	NA
evm.TU.Scaffold_40138_HRSCAF-40465.1946	54.28041705	2.096919814	0.543059072	3.861310712	0.00011278	0.099730846	NA	#N/A	#N/A	0.030666588	#N/A	evm.TU.Scaffold_40138_HRSCAF-40465.1946	NA	
evm.TU.Scaffold_40170_HRSCAF-40571.69	945.3428216	1.204340538	0.312170073	3.857962829	0.000114336	0.099730846	predicted protein [Nematostella vectensis]	0.086475133	0.018018222	#N/A	0.022170581	evm.TU.Scaffold_40170_HRSCAF-40571.69	predicted protein [Nematostella vectensis]	NA
